# Disease-modifying therapy in progressive multiple sclerosis: a systematic review and network meta-analysis of randomized controlled trials

**DOI:** 10.3389/fneur.2024.1295770

**Published:** 2024-03-11

**Authors:** Xin Wu, Shixin Wang, Tao Xue, Xin Tan, Jiaxuan Li, Zhouqing Chen, Zhong Wang

**Affiliations:** ^1^Department of Neurosurgery and Brain and Nerve Research Laboratory, The First Affiliated Hospital of Soochow University, Suzhou, Jiangsu, China; ^2^Department of Neurosurgery, Beijing Tiantan Hospital, Capital Medical University, Beijing, China; ^3^Department of Neurology, The Affiliated Suzhou Hospital of Nanjing Medical University, Suzhou Municipal Hospital, Suzhou, Jiangsu, China

**Keywords:** multiple sclerosis, disease-modifying therapy, randomized controlled, ocrelizumab, natalizumab, rituximab

## Abstract

**Background:**

Currently, disease-modifying therapies (DMTs) for progressive multiple sclerosis (PMS) are widely used in clinical practice. At the same time, there are a variety of drug options for DMTs, but the effect of the drugs that can better relieve symptoms and improve the prognosis are still inconclusive.

**Objectives:**

This systematic review aimed to evaluate the efficacy and safety of DMTs for PMS and to identify the best among these drugs.

**Methods:**

MEDLINE, EMBASE, the Cochrane Library, and clinicaltrials.gov were systematically searched to identify relevant studies published before 30 January, 2023. We assessed the certainty of the evidence using the confidence in the network meta-analysis (CINeMA) framework. We estimated the summary risk ratio (RR) for dichotomous outcomes and mean differences (MD) for continuous outcomes with 95% credible intervals (CrIs).

**Results:**

We included 18 randomized controlled trials (RCTs) involving 9,234 patients in the study. DMT can effectively control the disease progression of MS. Among them, mitoxantrone, siponimod, and ocrelizumab are superior to other drug options in delaying disease progression (high certainty). Mitoxantrone was the best (with high certainty) for mitigating deterioration (progression of disability). Ocrelizumab performed best on the pre- and post-treatment Timed 25-Foot Walk test (T25FW; low certainty), as did all other agents (RR range: 1.12–1.05). In the 9-Hole Peg Test (9HPT), natalizumab performed the best (high certainty), as did all other agents (RR range: 1.59–1.09). In terms of imaging, IFN-beta-1b performed better on the new T2 hypointense lesion on contrast, before and after treatment (high certainty), while siponimod performed best on the change from baseline in the total volume of lesions on T2-weighted image contrast before and after treatment (high certainty), and sWASO had the highest area under the curve (SUCRA) value (100%). In terms of adverse events (AEs), rituximab (RR 1.01), and laquinimod (RR 1.02) were more effective than the placebo (high certainty). In terms of serious adverse events (SAEs), natalizumab (RR 1.09), and ocrelizumab (RR 1.07) were safer than placebo (high certainty).

**Conclusion:**

DMTs can effectively control disease progression and reduce disease deterioration during the treatment of PMS.

**Systematic review registration:**

https://inplasy.com/?s=202320071, identifier: 202320071.

## Highlights

- DMTs can effectively control disease progression and reduce disease deterioration during the treatment of PMS.
- Ocrelizumab, Siponimod, and IFN-beta-1b have positive effects on delaying PMS disease progression.
- Rituximab and natalizumab are less effective in controlling disease progression.
- Mitoxantrone can effectively alleviate the progression of PMS, but there are more serious adverse reactions.

## Introduction

Multiple sclerosis (MS) is a classic inflammatory disease of the central nervous system (CNS) that manifests as a chronic, inflammatory, demyelinating disease that causes primary demyelinating plaques or neurodegeneration mainly in the white and gray matter of the brain and spinal cord (1). More than 2.5 million people worldwide are affected by MS, which is now recognized as the leading cause of non-traumatic neurological disability in adolescents (2). More than 80% of patients with MS enter remission soon after the onset of the active phase, and the disease can relapse and enter remission multiple times. When the disease is no longer in remission but continues to progress, it is called secondary progressive multiple sclerosis (SPMS). Approximately 10–20% of patients do not appear to be in remission but show continuous progression. This is referred to as primary progressive multiple sclerosis (PPMS) (3). Progressive multiple sclerosis (PMS) is characterized by progressive brain atrophy and cortical demyelination (4–6) in PPMS and SPMS. The effect of current treatments remain poor, this is because the longer the duration of PMS, the worse the recovery and higher the mortality (7).

For MS, anti-inflammatory or immunosuppressive therapy can significantly benefit patients by reducing the severity and frequency of new demyelinating episodes (8, 9). However, it does not effectively delay the disease progression of PMS (10); therefore more appropriate treatment is required. Since the advent of interferon, disease-modifying therapies (DMTs) have been applied to the treatment of MS patients. The gradual recognition of its effect has caused the evolution of this PMS treatment (11). At present, the mainstream treatment for PMS is DMTs, which can delay the progression of the disease and reduce the deterioration of the disease by oral or injectable DMT-related drugs (12). Currently, more than 10 drugs are included in DMT therapy, including ocrelizumab, natalizumab, rituximab (RTX), laquinimod, siponimod, fingolimod, interferon-beta-1b (IFN-beta-1b), interferon-beta-1a (IFN-beta-1a), glatiramer acetate (GA), mitoxantrone, and dimethyl fumarate (DF) (13). Additionally, new drugs are in the pipeline. However, there is still debate over the best drug, positive results for patients, and the high price (6, 14).

The primary objective of this meta-analysis was to compare the efficacy and safety of various agents in DMT therapy of PMS. We pooled the data from previous randomized controlled trials and conducted a systematic review and network meta-analysis (NMA) to investigate the efficacy and safety of different drugs in DMT for the treatment of PMS.

## Methods

### Study protocol

Before the project started, we drafted a research protocol following the Cochrane Collaboration format (15). The protocol for this systematic review was also prospectively registered in PROSPERO (INPLASY202320071).

### Eligibility criteria

Inclusion criteria: (1) study type: randomized control trial (RCT); (2) language restriction: available in English; (3) subjects: patients ≥18 years diagnosed with progressive MS, whether with a primary progressive course, or a secondary progressive course who met the 2017 McDonald diagnostic criteria (16); (4) interventions: DMT, including ocrelizumab, natalizumab, rituximab, laquinimod, siponimod, fingolimod, IFN-beta-1b, IFN-beta-1a, glatiramer acetate, mitoxantrone, dimethyl fumarate; (5) control: placebo; (6) outcomes: clinical outcomes included the expanded disability status scale (EDSS) and the number of patients with confirmed disease progression (CDP), in which CDP was defined as an increase in the EDSS of at least 1.0 point from baseline in 12 weeks; patients' evaluated outcomes included the timed 25-foot walk (T25FW) and the 9-hole peg test (9HPT); magnetic resonance imaging (MRI) outcomes including change in the volume of lesions on T2 and the number of patients with new or newly enlarged lesions in T2; safety outcomes included adverse events (AEs) and serious adverse events (SAEs), among them, those with the risk of death are serious adverse reactions. The included RCTs were not required to include all the outcomes mentioned above.

The exclusion criteria were as follows: (1) study type: retrospective studies, cohort studies, case reviews, and case reports; and (2) patients diagnosed with relapsing MS.

### Information sources and search strategy

MEDLINE, EMBASE, the Cochrane Library, and ClinicalTrials.gov were systematically searched to identify relevant studies published before 30 January, 2023. The following search strategy was employed: (disease modifying therapy [Title/Abstract]) AND (multiple sclerosis [Title/Abstract]) for MEDLINE; “disease modifying therapy”/exp AND “multiple sclerosis”/exp for EMBASE; “disease modifying therapy” in Title Abstract Keyword AND “multiple sclerosis” in Title Abstract Keyword for Cochrane Library; “disease modifying therapy | multiple sclerosis” for ClinicalTrials.gov. The detailed search strategy can be found in the Supplementary Table 1.

Additionally, the reference lists of RCTs, relevant systematic reviews, and meta-analyses were screened independently and manually to ensure a more comprehensive search.

### Study selection and data collection

According to the eligibility criteria mentioned above, two reviewers (SXW and XW) independently reviewed all titles, abstracts, and full-text articles searched from the four databases and reference lists of RCTs and relevant systematic reviews or meta-analyses. Discrepancies between the two authors were resolved by discussion or, if necessary, by a third author (XT) who did not participate in the data collection. We excluded duplicates and research articles in which the full text was not available. XT made the final decision on disputed data.

After selection and evaluation, all data from the included RCTs were extracted as follows: basic information and outcome events included for each RCT, inclusion and exclusion criteria, study design. All efficacy and safety outcomes are shown in the Supplementary Table 2.

### Risk of bias and quality of evidence

The risk of a bias plot was evaluated using Review Manager software (version 5.3). The uniform criteria of the Cochrane Collaboration were used to assess the risk of bias for RCTs (17, 18), which included selection bias, performance bias, detection bias, attrition bias, reporting bias, and other potential biases. Each bias criterion was classified as “low,” “high,” or “unclear.” The assessment was performed independently by SXW and XW. Disagreements were resolved by consulting a third author (JQL).

The certainty of direct and indirect evidence in network meta-analyses was assessed using the confidence in network meta-analysis framework (CINeMA) (18) according to the recommendations of the Grading of Recommendations, Assessment, Development, and Evaluation (i.e., “GRADE”) working group (19). Based on an assessment of the overall risk of bias (randomization, blinding, allocation concealment, and selective reporting), imprecision [95% confidence interval (CI) and sample size], inconsistency and indirectness (study population), and risk of publication bias (funding sources), XW and TX independently classified the overall quality of evidence as “high,” “moderate,” “low,” or “very low.” Disagreements were resolved by consultation with a third author (XT).

### Summary measures and synthesis of results

Network meta-analysis was performed for each outcome using R 3.5.2 software and gemtc R package (20). The Markov chain Monte Carlo methods involved four chains with over-dispersed initial values and Gibbs sampling based on 50,000 iterations after a burn-in phase of 20,000 iterations. We estimated summary risk ratio (RR) for dichotomous outcomes and mean differences (MD) for continuous outcomes with their 95% credible intervals (CrI) [CI for Bayesian framework and confidence interval (CI) for frequentist setting]. The chi-square *q*-test and *I*^2^ statistic were also used to evaluate heterogeneity between trials in the network meta-analysis. We analyzed inconsistencies between direct and indirect sources of evidence to determine consistency. We examined the goodness of fit of the consistency and inconsistency models and estimated the difference between the direct and indirect estimates for one of the three comparisons in each closed loop produced by the three partial evaluation procedures, all of which are compared with each other (21).

The surface under the curve ranking area (SUCRA) was created to rank the performance of different DMT treatments and placebos for each outcome. For each outcome, a larger SUCRA value indicated a better rank for the intervention. The ranking probabilities were calculated as cumulative probabilities, with each intervention being ranked. Two-tailed tests were performed for all analyses, and a *P*-value < 0.05 was considered statistically significant.

## Results

**Figure 1:**
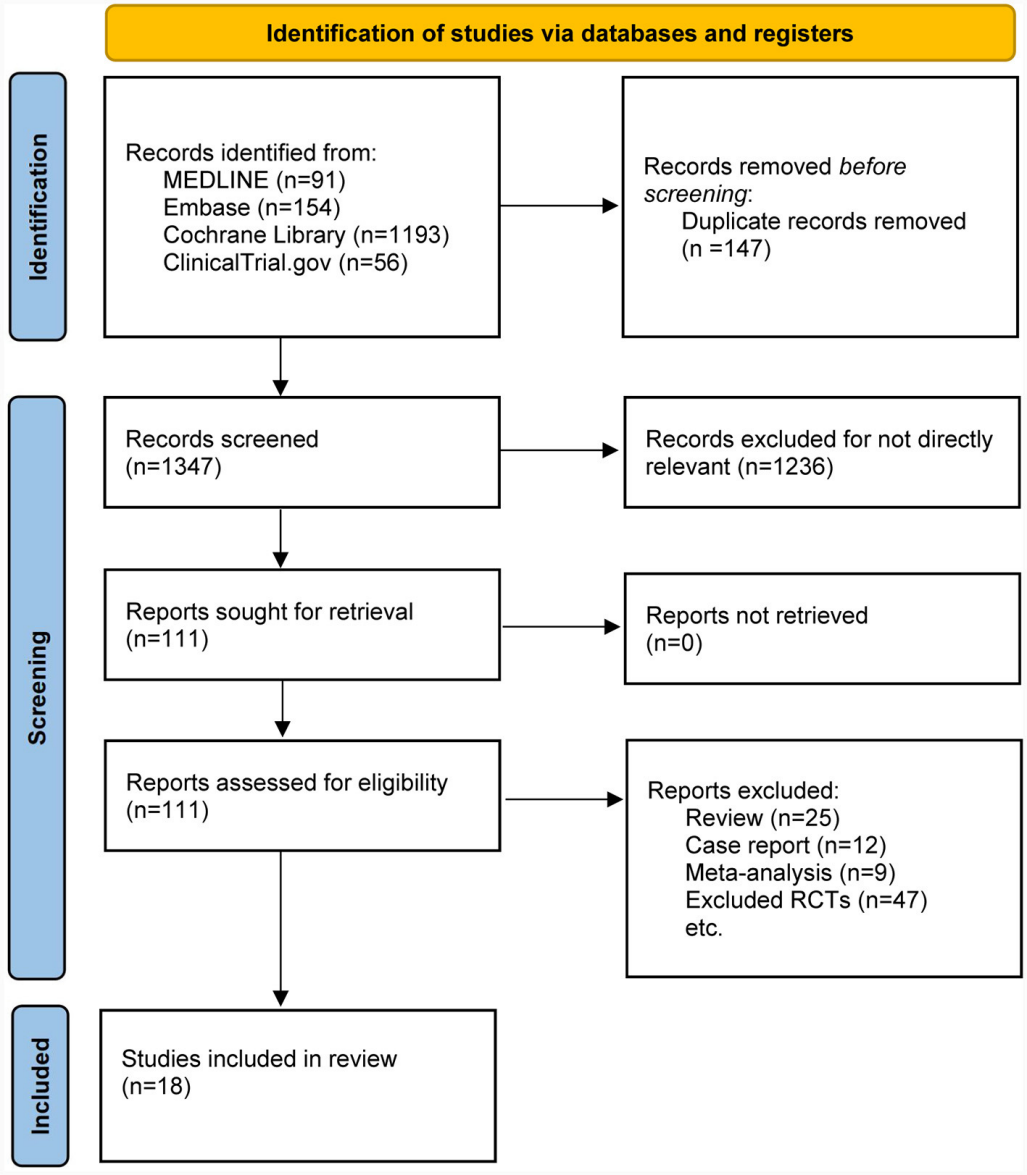
The study search, selection, and inclusion process.

**Figure 2:**
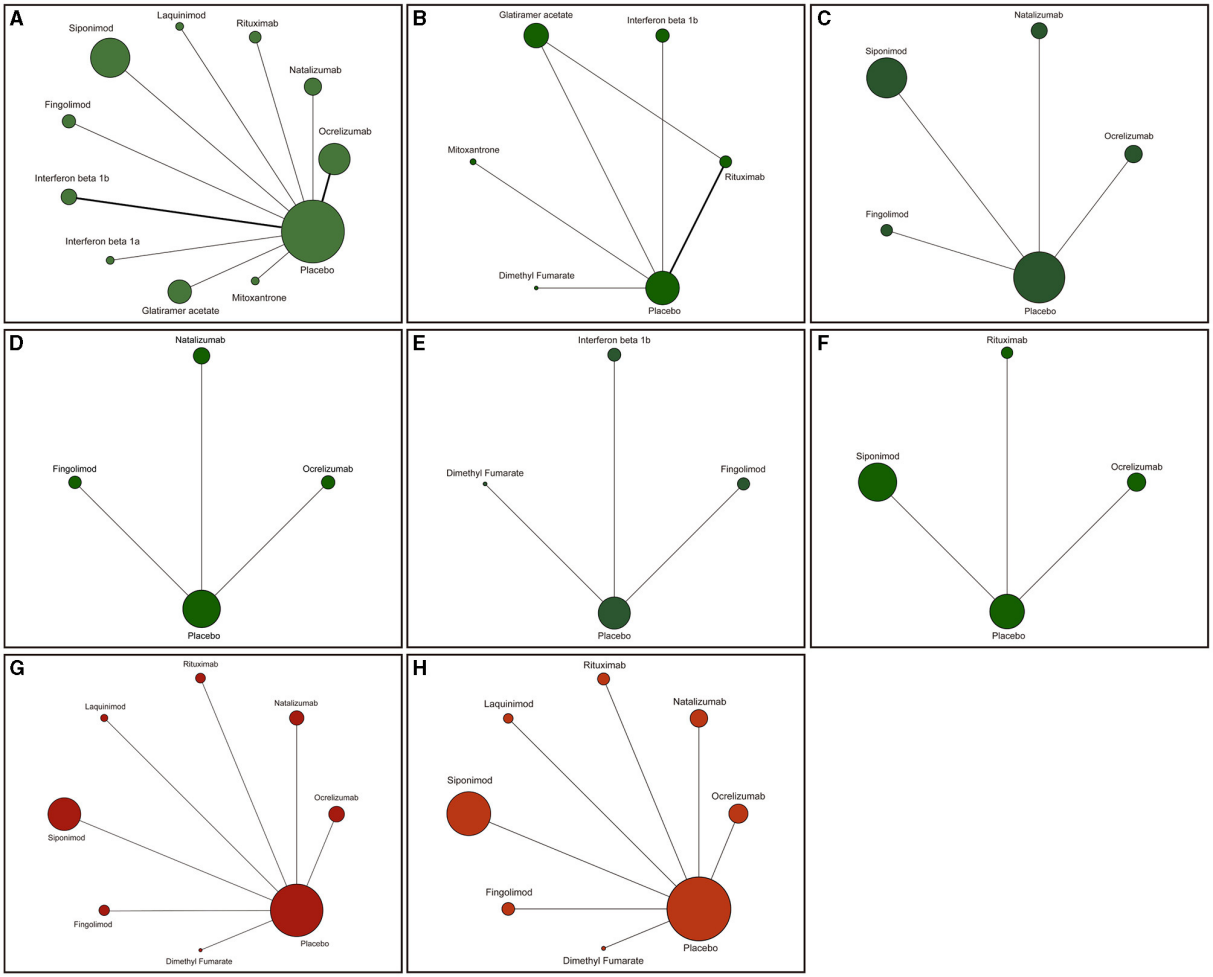
Network of randomized controlled trials (RCTs) comparing different disease-modifying therapies (DMTs) for progressive multiple sclerosis (PMS). The size of the circles represents the number of participants for each intervention and the width of lines represent the number of trials compared between treatments. Green colors represent efficacy outcomes and red colors represent safety outcomes. **(A)** Confirmed disability progression (CDP). **(B)** Expanded disability status scale (EDSS). **(C)** Confirmed worsening of at least 20% from baseline in the timed 25-foot walk test (T25FW). **(D)** Increase of 20% or more from baseline (on either hand) on the nine-hole peg test (9HPT). **(E)** New or newly enlarged lesions in T2. **(F)** Change in the volume of lesions on T2. **(G)** Adverse events (AEs). **(H)** Serious adverse events (SAEs). Values in bold indicate a significant difference.

PubMed, Embase, the Cochrane Library, and clinicaltrials.gov provided 1,494 titles and abstracts. A total of 1,383 articles were excluded owing to duplication and/or irrelevance after a quick review, and 111 full articles were assessed for eligibility. Among them, 129 articles were excluded due to inapplicable publication types or participants, including 25 reviews, 12 case reports, nine meta-analyses, and 47 RCTs that did not meet our inclusion criteria. The selection process is summarized in the flow diagram in Figure 1. In order to compare the scientific results more accurately, the included studies are generally a single treatment process carried out by certain drugs, and generally do not combine with other treatment measures. Finally, 18 RCTs were included in the network meta-analysis. The main characteristics of the 18 RCTs are presented in Table 1. The network relationships between various interventions are shown in Figure 2. The size of each circle represents the number of participants for each intervention, and the width of each line represents the number of trials compared between treatments.

**Table 1:** Characteristics of the included randomized controlled trials for patients with progressive multiple sclerosis and their outcome events.

**References**	**Countries**	**Centers**	**Study period**	**Treatment group, (no. of participants)**	**MS type**	**Mean age ±SD (year)**	**Male (%)**	**Baseline EDSS**	**Course of disease (year)**	**Outcome**
Cheshmavar et al. (22)	Iran	1	12 months	Rituximab 1,000 mg (37) vs. Glatiramer-acetate 40 mg (36)	SPMS	RTX: 40.92 ± 8.12	RTX: 24.3(9/37)	RTX: 3.05 ± 1.01	RTX: 11.41 ± 6.45	b,e
						GA: 45.72 ± 7.64	GA: 36.1(13/36)	GA: 3.28 ± 1.32	GA: 17.39 ± 7.53	
Højsgaard Chow et al. (23)	Danish	1	48 weeks	Dimethyl fumarate 240 mg (27) vs. PLA (27)	PPMS	DF: 55.7 ± 5.5	DF: 63.0 (17/27)	DF: 4.48 ± 0.50	DF: 14.3 ± 9.4	b,e,g,h
						PLA: 54.0 ± 6.6	PLA: 59.0 (16/27)	PLA: 4.19 ± 0.63	PLA: 13.8 ± 9.7	
Wolinsky et al. (24)	America	182	>120 weeks	Ocrelizumab 600 mg (367) vs. PLA (160)	PPMS	Ocrelizumab: 44.8 ± 7.8	Ocrelizumab: 53.0 (193/367)	Ocrelizumab: 4.6 ± 1.2	NR	a,c,d
						PLA: 45.6 ± 7.7	PLA: 49.0 (78/160)	PLA: 4.7 ± 1.2		
Giovannoni et al. (25)	UK	85	48 weeks	Laquinimod 0.6 mg (139) vs. Laquinimod 1.5 mg (95) vs. PLA (140)	PPMS	Laquinimod 0.6 mg: 46.1 ± 6.7	Laquinimod 0.6 mg: 59 (82/139)	Laquinimod 0.6 mg: 4.5 ± 1.0	Laquinimod 0.6 mg: 8.3 ± 6.3	a,g,h
						Laquinimod 1.5 mg: 46.1 ± 7.2	Laquinimod 1.5 mg: 50 (53/95)	Laquinimod 1.5 mg: 4.4 ± 1.0	Laquinimod 1.5 mg: 8.5 ± 5.6	
						PLA: 26.6 ± 7.2	PLA: 52 (73/140)	PLA: 4.5 ± 0.9	PLA: 7.4 ± 5.2	
Kappos et al. (26)	Switzerland	292	3 years	Siponimod 2 mg (1,105) vs. PLA (546)	SPMS	Siponimod: 48.0 ± 7.8	Siponimod: 39.0 (436/1,105)	Siponimod: 5.4 ± 1.1	Siponimod: 17.1 ± 8.4	a,c,f,g,h
						PLA: 48.1 ± 7.9	PLA: 41.0 (223/546)	PLA: 5.4 ± 1.0	PLA: 16.2 ± 8.2	
Kapoor et al. (27)	UK	163	2 years	Natalizumab 300 mg (440) vs. PLA (449)	SPMS	Natalizumab: 47.3 ± 7.4	Natalizumab: 38.0 (169/440)	Natalizumab: 5.99 ± 0.25	Natalizumab: 16.8 ± 7.6	a,c,d,g,h
						PLA: 47.2 ± 7.8	PLA: 37.0 (168/449)	PLA: 5.99 ± 0.25	PLA: 16.2 ± 7.8	
Montalban et al. (28)	America	184	>120 weeks	Ocrelizumab 600 mg (488) vs. PLA (244)	PPMS	Ocrelizumab: 44.7 ± 7.9	Ocrelizumab: 51.4 (251/488)	Ocrelizumab: 4.7 ± 1.2	Ocrelizumab: 6.7 ± 4.0	a,c,g,h
						PLA: 44.4 ± 8.3	PLA: 49.2 (120/244)	PLA: 4.7 ± 1.2	PLA: 6.1 ± 3.6	
Lublin et al. (29)	America	148	36 months-5 years	Fingolimod 0.5 mg (336) vs. PLA (487)	PPMS	Fingolimod: 48.5 ± 8.6	Fingolimod: 51 (173/336)	Fingolimod: 4.70 ± 1.03	Fingolimod: 5.8 ± 2.5	a,c,d,e,g,h
						PLA: 48.5 ± 8.3	PLA: 52 (252/487)	PLA: 4.66 ± 1.03	PLA: 5.9 ± 2.4	
Komori et al. (30)	America	1	2 years	Rituximab 200 mg (14) vs. PLA (9)	SPMS	RTX: 57.2 ± 7.5	RTX: 50.0 (7/14)	RTX: 5.88 ± 1.32	RTX: 26.4 ± 9.7	b
						PLA: 54.7 ± 8.0	PLA: 22.2 (2/9)	PLA: 6.17 ± 0.5	PLA: 25.8 ± 7.3	
Montalban et al. (31)	Spain	1	24 months	Interferon-beta 1b 8 MIU (36) vs. PLA (37)	PPMS	IFNb-1b: 48.8 ± 7.5	IFNb-1b: 61 (22/36)	IFNb-1b: 5.3 ± 1.2	IFNb-1b: 11.3 ± 6.4	a,f
						PLA: 48.6 ± 8.7	PLA: 40 (15/37)	PLA: 5.2 ± 1.2	PLA: 11.4 ± 6.8	
Hawker et al. (32)	Canada and America	60	122 weeks	Rituximab 1,000 mg (292) vs. PLA (147)	SPMS	RTX: 50.1 ± 9.0	RTX: 52.1 (152/292)	RTX: 4.8 ± 1.4	RTX: 9.2 ± 6.4	b,f,g,h
						PLA: 49.6 ± 8.7	PLA: 44.9 (66/147)	PLA: 4.7 ± 1.4	PLA: 9.6 ± 6.8	
Wolinsky et al. (33)	Canada	2	3 years	Glatiramer-acetate 20 mg (627) vs. PLA (316)	PPMS	GA: 50.4 ± 8.4	GA: 47.2 (296/627)	GA: 4.9 ± 1.2	RTX: 11.0 ± 7.3	a,b
						PLA: 50.2 ± 8.1	PLA: 51.9 (164/316)	PLA: 4.9 ± 1.2	PLA: 10.7 ± 7.7	
Andersen et al. (34)	Sweden	32	3 years	Interferon Beta-1a 22 μg (186) vs. PLA (178)	SPMS	IFNb-1a: 45.1	IFNb-1a: 40.0 (74/186)	IFNb-1a: 4.7	IFNb-1a: 14.2	a
						PLA: 46.4	PLA: 40.0 (71/178)	PLA: 5.0	PLA: 14.4	
Leary et al. (35)	UK	1	2 years	Interferon Beta-1a 30 μg (15) vs. Interferon Beta-1a 60 μg (15) vs. PLA (20)	PPMS	IFNb-1a 30 μg: 46.5 ± 8.3	IFNb-1a 30 μg: 66.7 (10/15)	IFNb-1a 30 μg: 5.41 ± 1.0	IFNb-1a 30 μg: 7.9 ± 4.3	d
						IFNb-1a 60 μg: 47.1 ± 9.8	IFNb-1a 60 μg: 46.7 (7/15)	IFNb-1a 60 μg: 5.07 ± 1.29	IFNb-1a 60 μg: 8.1 ± 5.2	
						PLA: 43.4 ± 7.8	PLA: 75.0 (15/20)	PLA: 4.5 ± 1.34	PLA: 8.0 ± 4.5	
Hartung et al. (36)	Germany	17	2 years	Mitoxantrone 5 mg/m^2^ (64) vs. Mitoxantrone 12 mg/m^2^ (60) vs. PLA (64)	SPMS	Mitoxantrone 5 mg/m^2^: 39.9 ± 8.1	Mitoxantrone 5 mg/m^2^: 39.0 (25/64)	Mitoxantrone 5 mg/m^2^: 4.64 ± 1.01	Mitoxantrone 5 mg/m^2^: 9.0 ± 6.2	a,b
						Mitoxantrone 12 mg/m^2^: 39.9 ± 6.9	Mitoxantrone 12 mg/m^2^: 53.0 (32/60)	Mitoxantrone 12 mg/m^2^: 4.45 ± 1.05	Mitoxantrone 12 mg/m^2^: 9.6 ± 6.9	
						PLA: 40.0 ± 7.9	PLA: 52.0 (33/64)	PLA: 4.69 ± 0.97	PLA: 10.3 ± 6.9	
Li et al. (37)	Switzerland	NR	3 years	Interferon Beta-1a 22 μg (209) vs. Interferon	SPMS	IFNb-1a 22 μg: 43.1 ± 7.2	IFNb-1a 22 μg: 38.3 (80/209)	IFNb-1a 22 μg: 5.5 ± 1.1	IFNb-1a 22 μg: 13.3 ± 7.4	a
				Beta-1a 44 μg (204) vs. PLA (205)		IFNb-1a 44 μg: 42.6 ± 7.3	IFNb-1a 44 μg: 32.8 (67/204)	IFNb-1a 44 μg: 5.3 ± 1.1	IFNb-1a 44 μg: 12.9 ± 6.9	
						PLA: 42.7 ± 6.8	PLA: 40.0 (82/205)	PLA: 5.4 ± 1.1	PLA: 13.7 ± 7.2	
Miller et al. (38)	Germany	32	3 years	Interferon Beta-1b 8 million IU (350) vs. PLA (345)	SPMS	NR	NR	NR	NR	e
Kappos et al. (26)	Switzerland	32	3 years	Interferon Beta-1b 8 million IU (360) vs. PLA (358)	SPMS	IFNb-1b: 41.1 ± 7.2	IFNb-1b: 41.9 (151/360)	IFNb-1b: 5.1 ± 1.2	IFNb-1b: 12.8 ± 6.6	a,b
						PLA: 40.9 ± 7.2	PLA: 35.8 (128/358)	PLA: 5.2 ± 1.2	PLA: 13.4 ± 7.5	

PLA, placebo; PPMS, primary progressive multiple sclerosis; SPMS, secondary-progressive multiple sclerosis; NR, not reported.

Outcome: a: Confirmed disability progression (CDP); b: Expanded disability status scale (EDSS); c: Confirmed worsening of at least 20% from baseline in the timed 25-foot walk test (T25FW); d: Increase of 20% or more from baseline (on either hand) on the nine-hole peg test (9HPT); e: New or enlarging T2 lesions; f: Change from baseline in the total volume of lesions on T2-weighted images (mm3); g: Adverse events (AEs); h: Serious adverse events (SAEs).

### The efficacy and safety of FDA-approved DMT drugs compared with placebo

Our efficacy outcomes included CDP, EDSS, T25FW, and 9HPT tests and two radiographic measures: new or enlarging T2 lesions and change from baseline in the total volume of lesions on T2-weighted images (mm3). For the EDSS test, both IFN-beta-1b and mitoxantrone were superior to placebo, with the MD (95% CrI) ranging between 0.17 (95% CrI 0.03–0.31) for IFN-beta-1b and 0.54 (95% CrI 0.26–0.82) for mitoxantrone. Ocrelizumab, siponimod, and IFN-beta-1b were all superior to placebo for CDP, with RR (95% CrI) ranging between 1.27 (95% CrI 1.08–1.48) for patients receiving ocrelizumab, 1.29 (95% CrI 1.07–1.56) in the siponimod group, and 0.17 (95% CrI 0.03–0.31) in the IFN-beta-1b group. For the T25FW test, there was no significant difference between the four drugs included in the comparison and the placebo groups. The RR (95% CrI) of patients in the ocrelizumab group ranged between 1.12 (95% CrI 0.99–1.27) and 1.01 (95% CrI 0.85–1.21) in the natalizumab group, 1.05 (95% CrI 0.93–1.19) in the siponimod group, and 1.04 (95% CrI 0.91–1.17) in the fingolimod group. For 9HPT test, the RR (95% CrI) of fingolimod was 1.09 (95% CrI 0.86–1.38), which was not statistically significant compared with placebo. The RR (95% CrI) ranged between 1.39 (95% CrI 1.10–1.77) for patients who received ocrelizumab and 1.59 (95% CrI 1.20–2.11) for those who received natalizumab, both superior to placebo. For new or enlarging T2 lesions, the RR (95% CrI) of IFN-beta-1b was 1.30 (95% CrI 1.19–1.43), which was better than that of placebo. There was no significant difference between the other drugs and the placebo for the change from baseline in the total volume of lesions on T2-weighted images, except that the MD (95% CrI) of patients in the rituximab group was 10.0 (95% CrI −81.31 to −101.31), which was not statistically significant compared with the placebo group. Patients who received ocrelizumab had an MD (95% CrI) ranging between −60.10 (95% CrI −62.71 to −57.49) and −613.10 (95% CrI −618.38 to −607.82) in the siponimod group, both of which were superior to the placebo group.

**Figure 3:**
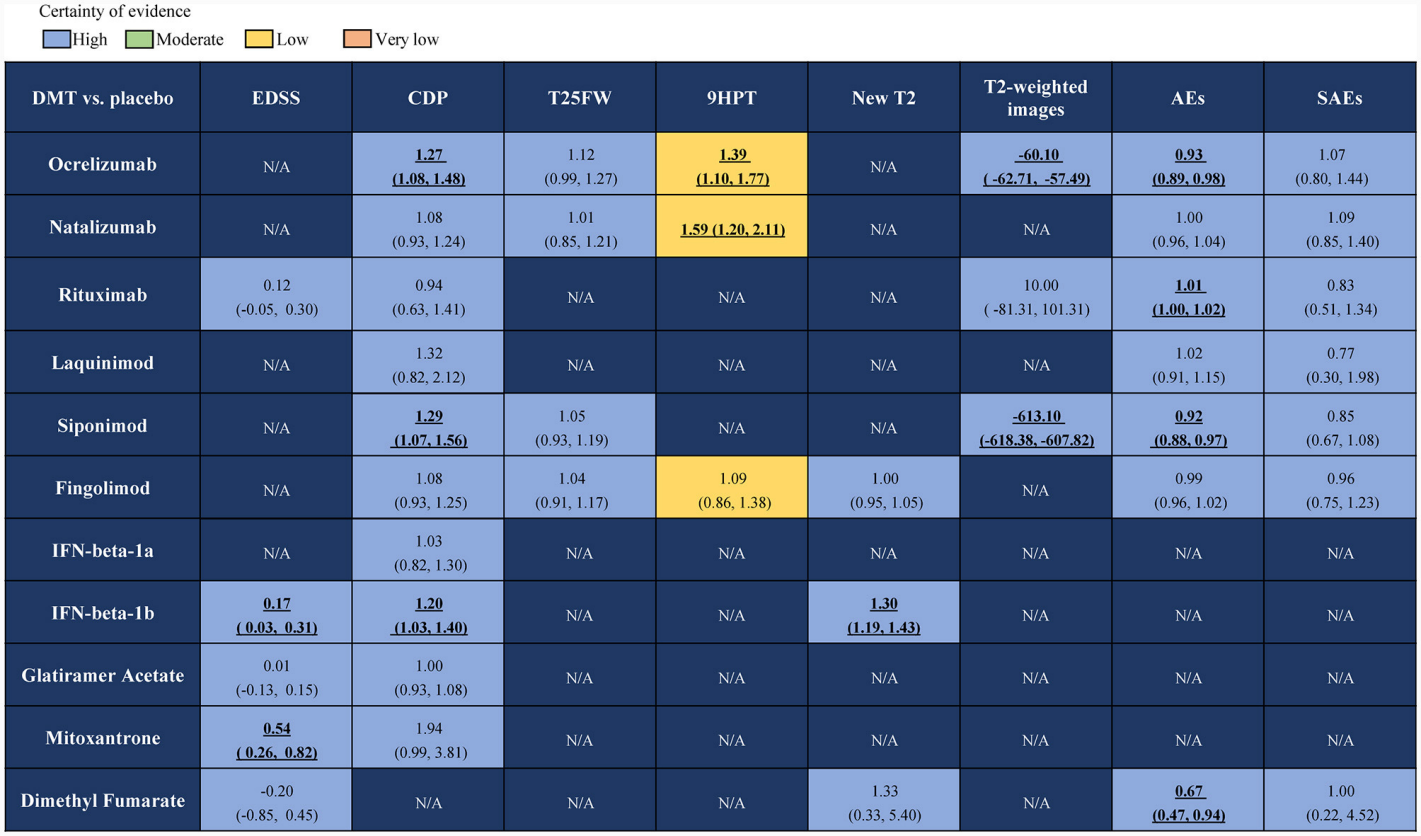
Network meta-analysis results of DMTs group compared with the placebo group for the treatment of PMS. DMTs, disease-modifying therapies; PMS, progressive multiple sclerosis; N/A, not available; CDP, confirmed disability progression; EDSS, expanded disability status scale; T25FW, the confirmed worsening of at least 20% from baseline in the timed 25-foot walk test; 9HPT, the increase of 20% or more from baseline (on either hand) on the nine-hole peg test; New T2, New or newly enlarged lesions in T2; T2-weighted images, Change from baseline in total volume of lesions on T2-weighted images (mm3); AEs, adverse events; SAEs, serious adverse events. Values in bold indicate significant difference.

In terms of safety, we pooled data from the seven included studies and found that RR (95% CrI) in patients with ocrelizumab ranging between 0.93 (95% CrI 0.89–0.98) and 1.01 (95% CrI 1.00–1.02) in Rituximab group, 0.92 (95% CrI 0.88–0.97) in siponimod group and 0.67 (95% CrI 0.47–0.94) in dimethyl fumarate group, which were better than the risk of adverse events in the placebo group. For SAEs, all drugs had a higher risk of serious adverse events than the placebo. Detailed results are presented in Figure 3.

### The efficacy and safety between each FDA-approved DMT drugs

**Figure 4:**
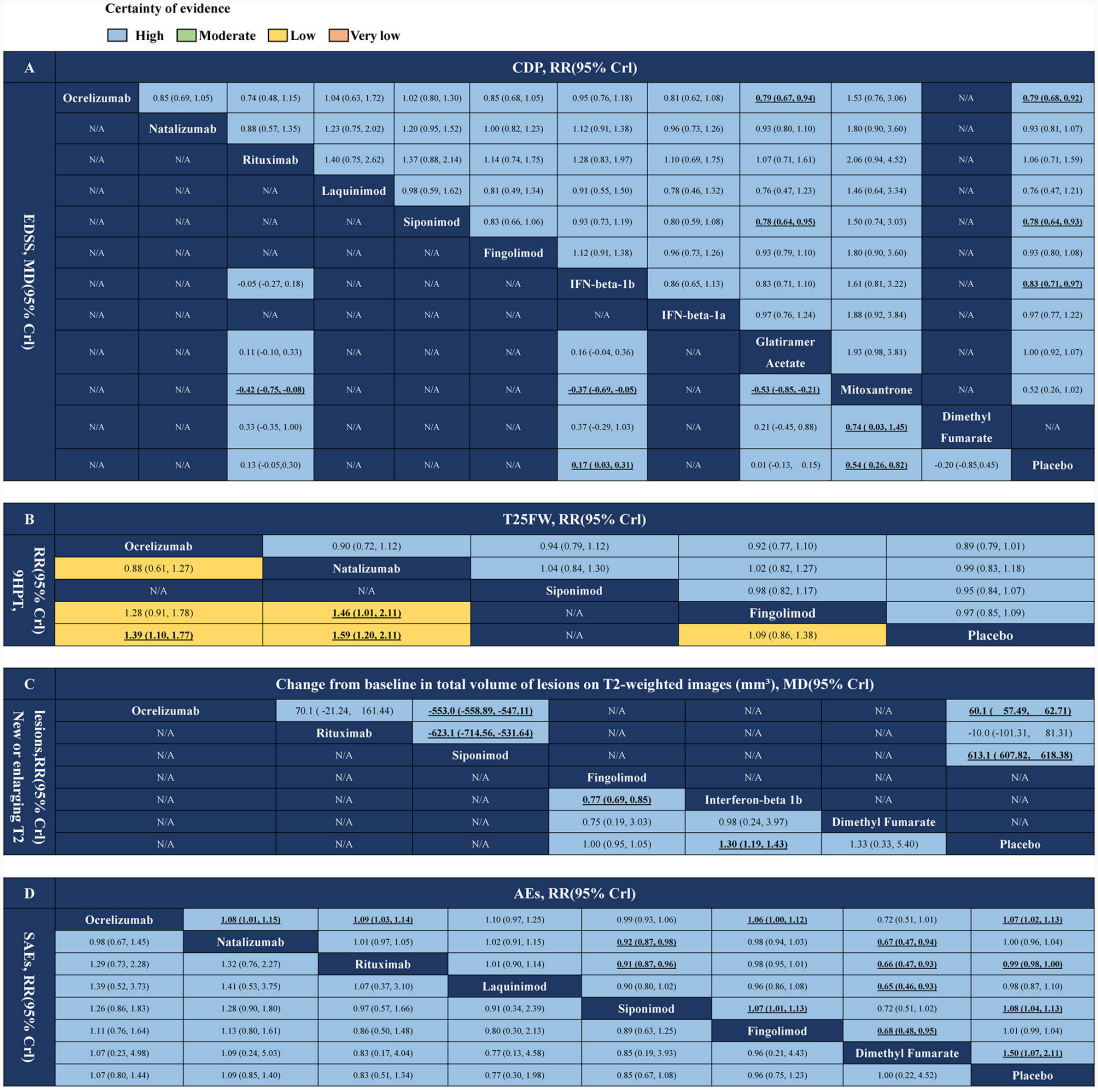
Network meta-analysis results of DMTs for PMS treatments. **(A)** CDP and EDSS. **(B)** T25FW and 9HPT. **(C)** New or newly enlarged lesions in T2 and changes in volume of lesions on T2. **(D)** Adverse events and serious adverse events. DMTs, disease-modifying therapies; PMS, progressive multiple sclerosis; N/A, not available; CDP, confirmed disability progression; EDSS, expanded disability status scale; T25FW, the confirmed worsening of at least 20% from baseline in the timed 25-foot walk test; 9HPT, the increase of 20% or more from baseline (on either hand) on the nine-hole peg test; AEs, adverse events; SAEs, serious adverse events. Values in bold indicate a significant difference.

The network estimates of all comparisons are shown in Figure 4. The color of each cell indicates the certainty of the evidence, according to GRADE. Further details of the GRADE evaluation are provided in Supplementary Table 3. The results showed that ocrelizumab and siponimod were superior (with high certainty) to glatiramer acetate in the CDP index evaluation. RR (95% CrI) were ranging between 0.79 (95% CrI 0.67–0.94) and 0.78 (95% CrI 0.64–0.95), respectively. There were no significant differences in other FDA-approved DMT drugs. In the index EDSS score, mitoxantrone was superior to either FDA-approved DMT drug (high certainty), and the other drug comparisons were not statistically significant (Figure 4A). In the index T25FW score, there was no statistically significant difference in drug comparisons. In the 9HPT index score, natalizumab was superior to fingolimod (low certainty), with RR (95% CrI) ranging between 1.46 (95% CrI 1.01–2.11), and there was no significant difference in other FDA-approved DMT drug comparisons (Figure 4B). In imaging index new or enlarging T2 lesions, IFN-beta-1b was superior to fingolimod (high certainty), with RR (95% CrI) ranging between 0.77 (95% CrI 0.69–0.85), and there was no significant difference in other FDA-approved DMT drug comparisons. Siponimod was significantly superior to ocrelizumab and rituximab (high certainty) in terms of change from baseline in total volume of lesions on T2-weighted images, MD (95% CrI) ranging between −553.0 (95% CrI −558.89 to −547.11) and −623.1 (95% CrI −714.56 to −531.64), respectively (Figure 4C). In terms of safety, rituximab, natalizumab, and fingolimod caused fewer adverse events (with high certainty) than ocrelizumab, siponimod, and dimethyl fumarate, and in terms of SAEs, there were no other significant differences between any of the FDA-approved DMT drug comparisons (Figure 4D).

### SUCRAs of FDA-approved DMT drugs and placebo

**Figure 5:**
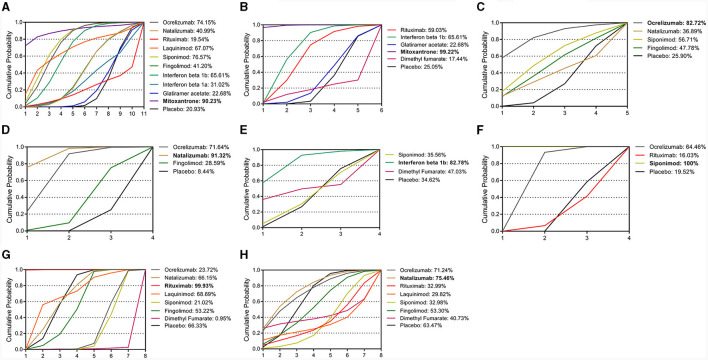
Cumulative probability of each intervention with specific ranks (1–5) for outcomes. A larger SUCRA value indicated a better rank for the intervention. **(A)** CDP. **(B)** EDSS. **(C)** T25FW. **(D)** 9HPT. **(E)** New or newly enlarged lesions in T2. **(F)** Change from baseline in total volume of lesions on T2-weighted images (mm3). **(G)** AEs. **(H)** SAEs. SUCRA, surface under curve ranking area; CDP, confirmed disability progression; EDSS, expanded disability status scale; T25FW, the confirmed worsening of at least 20% from baseline in the timed 25-foot walk test; 9HPT, the increase of 20% or more from baseline (on either hand) on the nine-hole peg test; AEs, adverse events; SAEs, serious adverse events. Values in bold indicate a significant difference.

As shown in Figure 5, the SUCRA values for six efficacy outcomes and two safety outcomes across 11 different drugs and a placebo showed that mitoxantrone had the highest SUCRA values for the CDP (90.2%) and EDSS (99.2%). Ocrelizumab showed the highest SUCRA value in the T25FW group (82.7%). Natalizumab showed the highest SUCRA value (91.3%) for 9HPT. In terms of imaging, new or enlarged T2 lesions had the highest SUCRA value for IFN-beta-1b (82.7%), and T2 volume had the highest SUCRA value for siponimod (100.0%). In terms of safety, rituximab had the highest SUCRA value (99.9%) among the adverse events, and natalizumab had the highest SUCRA value (75.4%) among the SAEs.

### Heterogeneity and inconsistency analysis of all outcomes

To analyse the heterogeneity among the selected studies in the network meta-analysis, we ran a heterogeneity analysis on each outcome we chose, comparing the difference between direct and indirect comparisons. Supplementary Figures 18, 19 depict the pairwise *I*^2^-values of the two efficacy outcomes. We used the node-splitting model to assess the differences between direct and indirect comparisons to determine the inconsistency of eight networks in which *I*^2^ was < 50%, including six efficacy and two safety outcomes. The purpose of this study was to assess the inconsistency between the direct and indirect evidence of a specific node (split node). We found no evident anomalies in the network model with indirect sources, as illustrated in Supplementary Figures 20–27. As a result, the inconsistent model results are reliable.

### Risk of bias in included studies

The risk of bias for all the enrolled studies is illustrated in Supplementary Figure 1. All the 18 included RCTs showed a low risk of bias in the random sequence generation. The risk of bias was high in only one trial for blinding of participants and personnel and blinding of outcome assessment. With incomplete outcome data, the risk of bias was unclear in 11 and high in one study. The risk of bias in selective reporting was unclear in six and high in one study. Aside from these items, an unclear risk of bias was observed in three RCTs.

## Discussion

The current analysis was based on 18 RCTs targeting pairwise comparisons between DMT and placebo treatments in patients with PMS. The results of the network meta-analysis found that, compared with the control group, DMT played a better role in all eight efficacy outcomes. Further network meta-analysis showed that mitoxantrone was superior to the other drugs in the EDSS test and CDP. Ocrelizumab showed the best performance in T25FW. Natalizumab showed the best performance in the 9HPT pre- and post-test comparisons; IFN-beta-1b and siponimod performed well in reducing the volume of T2-enhanced lesions and T2 lesions, respectively. Meanwhile, network meta-analysis showed that the use of DMT did not significantly increase the risk of AEs and SAEs.

Mitoxantrone was originally developed as a DNA intercalator for cancer treatments. In 1997, mitoxantrone was introduced as an induction drug for MS and then approved to reduce the deterioration of disability in patients with recurrent SPMS and RRMS (36, 39). It is used less frequently in current MS treatment due to the increased risk of cardiac toxicity and hematological malignancy in patients (40, 41). We performed a mesh analysis using a RCT with a cycle of 2 years, with a dose of 5–12 mg/m^2^, and the results showed that mitoxantrone performed best in delaying disease progression and EDSS tests, significantly better than the control group; however, the RCT analyses we included did not show evidence of drug-related serious adverse events or clinically significant cardiac dysfunction. At the same time, in a recent study involving 71 patients, mitoxantrone also showed more adverse events (42). Good efficacy is often accompanied by serious adverse events. Maintaining efficacy and improving prognosis are directions for future research.

Due to the uncertainty of the etiology of MS, in the 1980's, some studies began to investigate the effect of interferon (IFN) on MS based on the hypothesis of viral origin (43, 44). With the progress of experiments, the efficacy of interferon has gradually been confirmed (45). In 1993, the first approved IFN-beta-1b was used in clinical practice, after which other interferon preparations began to appear, including IFN-beta-1a and pegylated IFN- beta-1a (46). Through a meta-analysis of RCTs on the treatment of PMS with interferon, we found that interferon can delay disease progression in imaging and can reduce the number of T2 image enhancement lesions, especially IFN-beta-1b. IFN-beta-1a has a poor effect, but is better than the control group. If the dose of interferon is too low, it will have no effect on disease progression. However, the treatment cycle of interferon is longer, usually 3 years, which is also reflected in the studies we included. At present, pegylated IFN-beta-1a is mainly used in clinical practice, but there are few studies on PMS, and more studies are needed in the future to update and improve these results.

The clinical application of FDA-approved DMT drugs has gradually matured, particularly monoclonal antibody drugs, which are widely used in RRMS with good efficacy and prognosis. Currently, ocrelizumab, natalizumab, and rituximab are used for PMS. Ocrelizumab, a B-cell-depleting anti-CD 20 monoclonal antibody, was the first DMT approved for PPMS (11). Rituximab is an older monoclonal antibody approved for rheumatic and hematologic disorders that recognizes the same antigen on the CD 20 protein and is being tested for PMS because of its promising efficacy after its previous use in RRMS (32, 47). Natalizumab, a monoclonal antibody against human α-4 integrin, is an immunosuppressive agent that can be used to treat severe inflammatory bowel disease and relapsing multiple sclerosis (48). In our study, ocrelizumab showed the best performance in slowing disease progression and reducing disease exacerbation, with significantly better results than a placebo on three measures of efficacy and one measure of safety. Unexpectedly, rituximab did not show good efficacy in the treatment of PMS but showed a positive prognosis, which may be due to the small number of patients in two of the three studies included in the study, which affected the overall results. However, in general, the application of monoclonal antibody drugs, especially ocrelizumab, in patients with PMS has achieved good results, and we hope that more studies will improve this result.

Sphingosine-1-phosphate modulators (S1PM) can regulate G protein-coupled receptors, participate in many cell responses in the central nervous system, significantly improve the symptoms of MS patients, and have been widely used in DMT (49). Currently, S1PM used in clinical practice includes laquinimod, siponimod, fingolimod, ozanimod, and ponesimod (49), and there are also a large number of RCTs on MS studies. However, few studies have been conducted on PMS, only three of which involved laquinimod, siponimod, and fingolimod (25, 26, 29). Laquinimod can inhibit the entry of inflammatory factors, CD4+ cells, and macrophages into the central nervous tissue (spinal cord), change the balance of cytokines, and upregulate the cytokines Th2/Th3, IL-4, IL-10, and transforming growth factor β, which are similar to immunomodulatory drugs in nature (50–52). Under psychological and inflammatory pathological conditions, oral laquinimod can penetrate the central nervous system to exert neuroprotective effects (53). Fingolimod is a non-selective S1PM that inhibits the outflow of immune cells from lymph nodes (11, 54). It is the first S1PM approved for RRMS and has recently been used in patients with PMS, with promising results. Siponimod is a selective S1PM that protects the nervous system by preventing excitotoxic synaptic degeneration, enhancing cell survival pathways, and promoting myelin regeneration. It easily crosses the blood-brain barrier (BBB) and can exert good efficacy (55–58). In our study, a network analysis of these articles showed that siponimod was the most effective in delaying the progression of the disease, followed by laquinimod. At the same time, all three drugs were significantly better than the control group, regardless of the T25FW or 9HPT scores. S1PM showed good effects. Siponimod was more effective in reducing the volume of brain lesions on T2 imaging and was superior to the control group in reducing T2-enhanced lesions. The incidence of adverse events and serious adverse events was significantly higher in the Siponimod and Fingolimod groups than in the control group, which is similar to the results of the current study. The incidence of adverse events was lower in the laquinimod group than in the control group; however, the incidence of serious adverse events was significantly higher in the laquinimod group than in the control group. This finding may be attributable to the dose of laquinimod, which increased from 66 to 83% in the study when the dose of laquinimod decreased from 1.5 to 0.6 mg/Kg (25). In conclusion, for the treatment of PMS, S1PM can effectively delay the progression of the disease and reduce the volume of brain lesions. However, the poor prognosis is a disadvantage of this drug class, and future research is expected to compensate for this shortfall.

There is a class of DMT therapies called tolerance therapies, and glatiramer acetate belongs to this class. The specific mechanism of action of glatiramer acetate is not clear, but because of its similar effect with interferon, it has unique anti-inflammatory and immunomodulatory activities, and it is currently believed that it can play a role in the neuroprotective immune response (59, 60). Glatiramer acetate has been used in the treatment of MS patients for a long time, but its control effect has been poor. It has recently been used in PMS patients to broaden its application. In our results, glatiramer acetate did not show a significant reduction in disease progression or deterioration and did not perform well-compared with other drugs. Dimethyl fumarate was used as a treatment for psoriasis until 2008, when studies found its immunomodulatory effect in cells, listed it as a potential treatment for MS, and began to use it in clinical practice (61). Current clinical trials have shown that dimethyl fumarate has fewer gastrointestinal side effects and can achieve appropriate blood levels (62). This was also shown in our study, where dimethyl fumarate had a good safety profile with a lower incidence of adverse events than the placebo, but had no significant effect on the worsening condition of patients with PMS.

This meta-analysis has several limitations. First, after pooling the RCT data for the drugs included in the DMT, we were unable to reconcile the differences in treatment effects according to the different drug doses, which may have biased our findings. With an increase in drug dose, its efficacy tends to increase, and its safety will gradually decrease. Thus, this meta-analysis cannot provide clinicians with specific recommendations regarding PMS medication doses. Second, the target population of our included studies was people with PMS, both SPMS and PPMS; RRMS was excluded from this study. For different subtypes of MS, owing to the different pathogenesis and symptoms, the treatment methods and results are different, and the prognosis of patients is also different, which may cause certain deviations. Third, this study was based on limited data, and despite an extensive search, only 18 studies were selected for inclusion, with many DMT drugs having only one or no studies. At the same time, because the presentation of research data is different, much data cannot be integrated and compared uniformly, which will also have a certain impact on the overall analysis. However, the quality of the included studies was very high, and the authenticity and reliability of the data were worthy of recognition. Fourth, there was no consensus on the duration of treatment for PMS, and different studies varied in the duration of treatment for the included patients. Fifth, in order to get more accurate results, this study does not discuss that the application of joint treatment measures may have an impact on the analysis results, and the relevant contents need to be supplemented by more and more comprehensive RCTs in the future.

## Conclusion

We found that DMT can effectively control disease progression, reduce disease deterioration, and have a positive effect on the treatment of PMS, among which ocrelizumab, Siponimod, and IFN-beta-1b have positive effects on delaying disease progression. In addition, rituximab and natalizumab are effective in reducing the deterioration and prognosis of the disease but are less effective in controlling disease progression. Our findings suggest that DMT is a suitable treatment option for PMS. However, for different patients, individualized administration should be selected after evaluating treatment expectations, costs, and prognosis to achieve the optimal solution. Additional large-scale, high-quality studies are needed to further explore the efficacy and safety of these therapies.

## Data availability statement

The original contributions presented in the study are included in the article/Supplementary material, further inquiries can be directed to the corresponding authors.

## Author contributions

XW: Writing—original draft, Writing—review & editing. SW: Writing—original draft, Writing—review & editing. TX: Writing—review & editing. XT: Writing—review & editing. JL: Writing—review & editing. ZC: Writing—review & editing. ZW: Writing—review & editing.
