# Supplementary material for: Disease-modifying therapy in progressive multiple sclerosis: a systematic review and network meta-analysis of randomized controlled trials
Source: Front Neurol. 2024 Mar 11;15:1295770. doi: 10.3389/fneur.2024.1295770 (PMC10962394; doi:10.3389/fneur.2024.1295770)

Disease-Modifying Therapy in Progressive Multiple Sclerosis： A Systematic Review and Network Meta-Analysis of Randomized Controlled Trials

Xin Wu 1 #, Shixin Wang 2 #, Tao Xue 3#, Xin Tan 4, Jiaxuan Li 2, Zhouqing Chen 1 *, Zhong Wang 1*

1 Department of Neurosurgery & Brain and Nerve Research Laboratory, The First Affiliated Hospital of Soochow University, Suzhou, Jiangsu Province, China

2 Department of Neurosurgery & Brain and Nerve Research Laboratory, The First Affiliated Hospital of Soochow University, Suzhou, Jiangsu Province, China

3 Department of Neurosurgery, Beijing Tiantan Hospital, Capital Medical University, Beijing, China

4 Department of Neurology, The Affiliated Suzhou Hospital of Nanjing Medical University, Suzhou Municipal Hospital, Suzhou, Jiangsu Province, China

# Shixin Wang and Xin Wu contribute equally to this work.

***Correspondence:**

Zhong Wang, Department of Neurosurgery, The First Affiliated Hospital of Soochow University, 188 Shizi Street, Suzhou 215006, China. Email address: wangzhong761@163.com.

Zhouqing Chen, Department of Neurosurgery, The First Affiliated Hospital of Soochow University, 188 Shizi Street, Suzhou 215006, China. Email address: zqchen6@163.com.

Figure legends

Table S1: Detailed search strategy.

Table S2: Inclusion, exclusion criteria, study design and outcome assessments of the included studies.

Table S3: Detailed certainty of evidence for each outcome in league table.

Figure S1: Risk of bias

Figure S2: Convergence diagnostics of the network meta-analysis: EDSS.

Figure S3: Convergence diagnostics of the network meta-analysis: CDP.

Figure S4: Convergence diagnostics of the network meta-analysis: T25FW.

Figure S5: Convergence diagnostics of the network meta-analysis: 9HPT.

Figure S6: Convergence diagnostics of the network meta-analysis: New or enlarging T2 lesions.

Figure S7: Convergence diagnostics of the network meta-analysis: Change from baseline in total volume of lesions on T2-weighted images (mm^3).

Figure S8: Convergence diagnostics of the network meta-analysis: AEs.

Figure S9: Convergence diagnostics of the network meta-analysis: SAEs.

Figure S10: Trace and density of the network meta-analysis: EDSS.

Figure S11: Trace and density of the network meta-analysis: CDP.

Figure S12: Trace and density of the network meta-analysis: T25FW.

Figure S13: Trace and density of the network meta-analysis: 9HPT.

Figure S14: Trace and density of the network meta-analysis: New or enlarging T2 lesions.

Figure S15: Trace and density of the network meta-analysis: Change from baseline in total volume of lesions on T2-weighted images (mm^3).

Figure S16: Trace and density of the network meta-analysis: AEs.

Figure S17: Trace and density of the network meta-analysis: SAEs.

Figure S18: Forest plots for the heterogeneity: EDSS.

Figure S19: Forest plots for the heterogeneity: CDP.

Figure S20: Forest plots for the inconsistency: EDSS.

Figure S21: Forest plots for the inconsistency: CDP.

Figure S22: Forest plots for the inconsistency: T25FW.

Figure S23: Forest plots for the inconsistency: 9HPT.

Figure S24: Forest plots for the inconsistency: New or enlarging T2 lesions.

Figure S25: Forest plots for the inconsistency: Change from baseline in total volume of lesions on T2-weighted images (mm^3).

Figure S26: Forest plots for the inconsistency: AEs.

Figure S27: Forest plots for the inconsistency: SAEs.

Table S1: Full search strategy.

PubMed:

Search	Query	Results
#1	"Multiple Sclerosis, Chronic Progressive"[Mesh]	2507
#2	(((((((Chronic Progressive Multiple Sclerosis[Title/Abstract]) OR (Multiple Sclerosis, Progressive Relapsing[Title/Abstract])) OR (Multiple Sclerosis, Remittent Progressive[Title/Abstract])) OR (Remittent Progressive Multiple Sclerosis[Title/Abstract])) OR (Progressive Relapsing Multiple Sclerosis[Title/Abstract])) OR (Multiple Sclerosis, Secondary Progressive[Title/Abstract])) OR (Secondary Progressive Multiple Sclerosis[Title/Abstract])) OR (Multiple Sclerosis, Primary Progressive[Title/Abstract])) OR (Primary Progressive Multiple Sclerosis[Title/Abstract])	1748
#3	#1 OR #2	3426
#4	"teriflunomide" [Supplementary Concept]	492
#5	((((((((((((Z)-2-cyano-3-hydroxy-N-(4-(trifluoromethyl)phenyl)-2-butenamide[Title/Abstract]) OR ((2Z)-2-cyano-3-hydroxy-N-(4-(trifluoromethyl)phenyl)-2-butenamide[Title/Abstract])) OR (2-cyano-3-hydroxy-N-(4-(trifluoromethyl)phenyl)-2-butenamide[Title/Abstract])) OR (2-hydroxyethylidene-cyanoacetic acid-4-trifluoromethyl anilide[Title/Abstract])) OR (RS 61980[Title/Abstract])) OR (Aubagio[Title/Abstract])) OR (HMR1726[Title/Abstract])) OR (HMR-1726[Title/Abstract])) OR (A 771726[Title/Abstract])) OR (A 1726[Title/Abstract])) OR (A771726[Title/Abstract])) OR (A-771726[Title/Abstract])) OR (A77 1726[Title/Abstract])	249
#6	#4 OR #5	604
#7	"Interferon-beta"[Mesh]	10329
#8	(((((((((((Interferon beta[Title/Abstract]) OR (Interferon, Fibroblast[Title/Abstract])) OR (Fibroblast Interferon[Title/Abstract])) OR (Interferon, beta[Title/Abstract])) OR (beta Interferon[Title/Abstract])) OR (beta-Interferon[Title/Abstract])) OR (Fiblaferon[Title/Abstract])) OR (beta-1 Interferon[Title/Abstract])) OR (beta 1 Interferon[Title/Abstract])) OR (Interferon-beta1[Title/Abstract])) OR (Interferon beta1[Title/Abstract])) OR (Interferon, beta-1[Title/Abstract])) OR (Interferon, beta 1[Title/Abstract])	10510
#9	#7 OR #8	15387
#10	"Alemtuzumab"[Mesh]	2243
#11	(((((((((((((((Campath 1H[Title/Abstract]) OR (Monoclonal Antibody Campath-1H[Title/Abstract])) OR (Antibody Campath-1H, Monoclonal[Title/Abstract])) OR (Campath-1H, Monoclonal Antibody[Title/Abstract])) OR (Monoclonal Antibody Campath 1H[Title/Abstract])) OR (Campath-1H[Title/Abstract])) OR (Campath1H[Title/Abstract])) OR (Campath 1M[Title/Abstract])) OR (Campath-1M[Title/Abstract])) OR (MabCambath[Title/Abstract])) OR (Lemtrada[Title/Abstract])) OR (Campath 1G[Title/Abstract])) OR (Campath-1-G[Title/Abstract])) OR (Campath 1 G[Title/Abstract])) OR (Campath1G[Title/Abstract])) OR (Campath-1G[Title/Abstract])) OR (Campath[Title/Abstract])	826
#12	#10 OR #11	2634
#13	"Mitoxantrone"[Mesh]	4395
#14	((((((((((((((((((((((((((Mitozantrone[Title/Abstract]) OR (DHAQ[Title/Abstract])) OR (NSC-279836[Title/Abstract])) OR (NSC 279836[Title/Abstract])) OR (NSC279836[Title/Abstract])) OR (NSC-287836[Title/Abstract])) OR (NSC 287836[Title/Abstract])) OR (NSC287836[Title/Abstract])) OR	723

	(NSC-299195[Title/Abstract])) OR (NSC 299195[Title/Abstract])) OR (NSC299195[Title/Abstract])) OR (NSC-301739[Title/Abstract])) OR (NSC 301739[Title/Abstract])) OR (NSC301739[Title/Abstract])) OR (NSC-301739D[Title/Abstract])) OR (NSC 301739D[Title/Abstract])) OR (NSC301739D[Title/Abstract])) OR (Mitroxone[Title/Abstract])) OR (Pralifan[Title/Abstract])) OR (CL-232325[Title/Abstract])) OR (CL 232325[Title/Abstract])) OR (CL232325[Title/Abstract])) OR (Mitoxantrone Acetate[Title/Abstract])) OR (Acetate, Mitoxantrone[Title/Abstract])) OR (Mitoxantrone Hydrochloride[Title/Abstract])) OR (Hydrochloride, Mitoxantrone[Title/Abstract])) OR (Novantrone[Title/Abstract])) OR (Ralenova[Title/Abstract])) OR (Novantron[Title/Abstract])) OR (Onkotrone[Title/Abstract])	
#15	#13 OR #14	4692
#16	"Natalizumab"[Mesh]	1861
#17	(Tysabri[Title/Abstract]) OR (Antegren[Title/Abstract])	195
#18	#16 OR #17	1937
#19	"Dimethyl Fumarate"[Mesh]	950
#20	(((((((((((((((((Fumarate, Dimethyl[Title/Abstract]) OR (2-butenedioic acid, (2E)-, dimethyl ester[Title/Abstract])) OR (Dimethylfumarate[Title/Abstract])) OR (BG 00012[Title/Abstract])) OR (00012, BG[Title/Abstract])) OR (BG00012[Title/Abstract])) OR (BG-00012[Title/Abstract])) OR (Tecfidera[Title/Abstract])) OR (FAG 201[Title/Abstract])) OR (201, FAG[Title/Abstract])) OR (FAG201[Title/Abstract])) OR (FAG-201[Title/Abstract])) OR (Fumaderm[Title/Abstract])) OR (BG 12 compound[Title/Abstract])) OR (12 compound, BG[Title/Abstract])) OR (compound, BG 12[Title/Abstract])) OR (BG12 compound[Title/Abstract])) OR (compound, BG12[Title/Abstract])) OR (BG-12 compound[Title/Abstract])	382
#21	#19 OR #20	1121
#22	"Fingolimod Hydrochloride"[Mesh]	2623
#23	((((((2-Amino-2-(2-(4-octylphenyl)ethyl)-1,3-propanediol hydrochloride[Title/Abstract]) OR (FTY-720[Title/Abstract])) OR (FTY 720[Title/Abstract])) OR (FTY720[Title/Abstract])) OR (Gilenya[Title/Abstract])) OR (Gilenia[Title/Abstract])) OR (Fingolimod[Title/Abstract])	3822
#24	#22 OR #23	4075
#25	"Glatiramer Acetate"[Mesh]	1486
#26	((((((Acetate, Glatiramer[Title/Abstract]) OR (Copaxone[Title/Abstract])) OR (Glatiramer[Title/Abstract])) OR (TV 5010[Title/Abstract])) OR (5010, TV[Title/Abstract])) OR (TV5010[Title/Abstract])) OR (TV-5010[Title/Abstract])	2027
#27	#25 OR #26	2382
#28	"laquinimod" [Supplementary Concept]	120
#29	((ABR 215062[Title/Abstract]) OR (ABR215062[Title/Abstract])) OR (ABR-215062[Title/Abstract])	8
#30	#28 OR #29	121
#31	"peginterferon beta-1a" [Supplementary Concept]	59
#32	(((polyethylene glycol-interferon-beta-1a[Title/Abstract]) OR (PEG IFN-beta-1a[Title/Abstract])) OR (polyethylene glycol-interferon beta-1a[Title/Abstract])) OR (Plegridy[Title/Abstract])	29
#33	#31 OR #32	76
#34	"ocrelizumab" [Supplementary Concept]	297
#35	(((((((Ocrevus[Title/Abstract]) OR (R 1594[Title/Abstract])) OR (R1594[Title/Abstract])) OR (R-1594[Title/Abstract])) OR (RG-1594[Title/Abstract])) OR (PR 070769[Title/Abstract]))	29

	OR (PR070769[Title/Abstract])) OR (PR-070769[Title/Abstract])	
#36	#34 OR #35	319
#37	"Rituximab"[Mesh]	17869
#38	((((((((CD20 Antibody, Rituximab[Title/Abstract]) OR (Rituximab CD20 Antibody[Title/Abstract])) OR (Mabthera[Title/Abstract])) OR (IDEC-C2B8 Antibody[Title/Abstract])) OR (IDEC C2B8 Antibody[Title/Abstract])) OR (IDEC-C2B8[Title/Abstract])) OR (IDEC C2B8[Title/Abstract])) OR (GP2013[Title/Abstract])) OR (Rituxan[Title/Abstract])	1017
#39	#37 OR #38	18231
#40	#6 OR #9 OR #12 OR #15 OR #18 OR #21 OR #24 OR #27 OR #30 OR #33 OR #36 OR #39	47735
#41	#3 OR #40	584
#42	Randomized controlled trial[Filter]	578680
#43	#41 OR #42	91

Embass:

Search	Query	Results
#1	'progressive multiple sclerosis'/exp	4406
#2	'chronic progressive multiple sclerosis':ti,ab	291
#3	'multiple sclerosis, chronic progressive':ti,ab	3
#4	'multiple sclerosis, progressive relapsing':ti,ab	0
#5	'multiple sclerosis, remittent progressive':ti,ab	1
#6	'remittent progressive multiple sclerosis':ti,ab	2
#7	'progressive relapsing multiple sclerosis':ti,ab	19
#8	'multiple sclerosis, secondary progressive':ti,ab	17
#9	'secondary progressive multiple sclerosis':ti,ab	1546
#10	'multiple sclerosis, primary progressive':ti,ab	14
#11	'primary progressive multiple sclerosis':ti,ab	1125
#12	#1 OR #2 OR #3 OR #4 OR #5 OR #6 OR #7 OR #8 OR #9 OR #10 OR #11	4420
#13	'teriflunomide'/exp	4,170
#14	'(z)-2-cyano-3-hydroxy-n-(4- (trifluoromethyl)phenyl)-2-butenamide':ti,ab	0
#15	'(2z)-2-cyano-3-hydroxy-n-(4-(trifluoromethyl)phenyl)-2-butenamide':ti,ab	0
#16	'2-cyano-3-hydroxy-n-(4-(trifluoromethyl)phenyl)-2-butenamide':ti,ab	2
#17	'2-hydroxyethylidene-cyanoaceticacid-4-trifluoromethyl anilide':ti,ab	1
#18	'rs 61980':ti,ab	2
#19	'aubagio':ti,ab	71
#20	'hmr1726':ti,ab	3
#21	'hmr-1726':ti,ab	0
#22	'a 771726':ti,ab	7
#23	'a 1726':ti,ab	10
#24	'a771726':ti,ab	112
#25	'a-771726':ti,ab	7
#26	'a77 1726':ti,ab	163
#27	#13 OR #14 OR #15 OR #16 OR #17 OR #18 OR #19 OR #20 OR #21 OR #22 OR #23 OR #24 OR #25 OR #26	4231
#28	'beta interferon'/exp	342,993

#29	'interferon beta':ab,ti	6,578
#30	'interferon, fibroblast':ab,ti	7
#31	'beta interferon':ab,ti	527
#32	'interferon, beta':ab,ti	6,578
#33	'beta interferon':ab,ti	1,728
#34	'beta-interferon':ab,ti	1,728
#35	'fiblaferon':ab,ti	14
#36	'beta-1 interferon':ab,ti	10
#37	'beta 1 interferon':ab,ti	10
#38	'interferon-beta1':ab,ti	45
#39	'interferon beta1':ab,ti	45
#40	'interferon, beta-1':ab,ti	155
#41	'interferon, beta 1':ab,ti	155
#42	#28 OR #29 OR #30 OR #31 OR #32 OR #33 OR #34 OR #35 OR #36 OR #37 OR #38 OR #39 OR #40 OR #41	344,107
#43	'alemtuzumab'/exp	18,626
#44	'campath 1h':ab,ti	628
#45	'monoclonal antibody campath-1h':ab,ti	40
#46	'antibody campath-1h, monoclonal':ab,ti	0
#47	'campath-1h, monoclonal antibody':ab,ti	6
#48	'monoclonal antibody campath 1h':ab,ti	40
#49	'campath-1h':ab,ti	629
#50	'campath1h':ab,ti	523
#51	'campath 1m':ab,ti	47
#52	'campath-1m':ab,ti	47
#53	'lemtrada':ab,ti	55
#54	'mabcambath':ab,ti	0
#55	'campath 1g':ab,ti	81
#56	'campath-1-g':ab,ti	7
#57	'campath 1 g':ab,ti	7
#58	'campath1g':ab,ti	58
#59	'campath-1g':ab,ti	81
#60	'campath':ab,ti	1448
#61	#43 OR #44 OR #45 OR #46 OR #47 OR #48 OR #49 OR #50 OR #51 OR #52 OR #53 OR #54 OR #55 OR #56 OR #57 OR #58 OR #59 OR #60	18889
#62	'mitoxantrone'/exp	25,064
#63	'mitozantrone':ab,ti	133
#64	'dhaq':ab,ti	118
#65	'nsc-279836':ab,ti	10
#66	'nsc 279836':ab,ti	10
#67	'nsc279836':ab,ti	3
#68	'nsc-287836':ab,ti	1
#69	'nsc 287836':ab,ti	1
#70	'nsc287836':ab,ti	0
#71	'nsc-299195':ab,ti	0
#72	'nsc 299195':ab,ti	0

#73	'nsc299195':ab,ti	0
#74	'nsc-301739':ab,ti	18
#75	'nsc 301739':ab,ti	18
#76	'nsc301739':ab,ti	3
#77	'nsc-301739d':ab,ti	2
#78	'nsc 301739d':ab,ti	2
#79	'nsc301739d':ab,ti	0
#80	'mitroxone':ab,ti	0
#81	'pralifan':ab,ti	0
#82	'cl-232325':ab,ti	0
#83	'cl 232325':ab,ti	0
#84	'cl232325':ab,ti	0
#85	'mitoxantrone acetate':ab,ti	0
#86	'acetate, mitoxantrone':ab,ti	29
#87	'mitoxantrone hydrochloride':ab,ti	70
#88	'hydrochloride, mitoxantrone':ab,ti	0
#89	'novantrone':ab,ti	178
#90	'ralenova':ab,ti	2
#91	'novantron':ab,ti	14
#92	'onkotrone':ab,ti	2
#93	#62 OR #63 OR #64 OR #65 OR #66 OR #67 OR #68 OR #69 OR #70 OR #71 OR #72 OR #73 OR #74 OR #75 OR #76 OR #77 OR #78 OR #79 OR #80 OR #81 OR #82 OR #83 OR #84 OR #85 OR #86 OR #87 OR #88 OR #89 OR #90 OR #91 OR #92	25152
#94	'natalizumab'/exp	12,594
#95	'tysabri':ab,ti	464
#96	'antegren':ab,ti	21
#97	#94 OR #95 OR #96	12,604
#98	'dimethyl fumarate'/exp	5,862
#99	'fumarate, dimethyl':ab,ti	13
#100	'2-butenedioic acid, (2e)-, dimethyl ester':ab,ti	0
#101	'dimethylfumarate':ab,ti	533
#102	'bg 00012':ab,ti	2
#103	'00012, bg':ab,ti	1
#104	'bg00012':ab,ti	14
#105	'bg-00012':ab,ti	2
#106	'tecfidera':ab,ti	168
#107	'fag 201':ab,ti	4
#108	'201, fag':ab,ti	0
#109	'fag201':ab,ti	4
#110	'fag-201':ab,ti	4
#111	'fumaderm':ab,ti	117
#112	'bg 12 compound':ab,ti	0
#113	'12 compound, bg':ab,ti	0
#114	'compound, bg 12':ab,ti	0
#115	'bg12 compound':ab,ti	0
#116	'compound, bg12':ab,ti	0

#117	'bg-12 compound':ab,ti	0
#118	#98 OR #99 OR #100 OR #101 OR #102 OR #103 OR #104 OR #105 OR #106 OR #107 OR #108 OR #109 OR #110 OR #111 OR #112 OR #113 OR #114 OR #115 OR #116 OR #117	6031
#119	'fingolimod'/exp	12,106
#120	'2-amino-2-(2-(4-octylphenyl)ethyl)-1,3-propanediol hydrochloride':ab,ti	11
#121	'fty-720':ab,ti	143
#122	'fty 720':ab,ti	143
#123	'fty720':ab,ti	2752
#124	'gilenya':ab,ti	376
#125	'gilenia':ab,ti	3
#126	'fingolimod':ab,ti	5667
#127	#119 OR #120 OR #121 OR #122 OR #123 OR #124 OR #125 OR #126	12541
#128	'glatiramer'/exp	10,279
#129	'acetate, glatiramer':ab,ti	3
#130	'copaxone':ab,ti	620
#131	'glatiramer':ab,ti	4,285
#132	'tv 5010':ab,ti	5
#133	'5010, tv':ab,ti	0
#134	'tv5010':ab,ti	5
#135	'tv-5010':ab,ti	5
#136	'glatiramer acetate':ab,ti	4092
#137	#128 OR #129 OR #130 OR #131 OR #132 OR #133 OR #134 OR #135 OR #136	10495
#138	'laquinimod'/exp	914
#139	'abr 215062':ab,ti	9
#140	'abr215062':ab,ti	9
#141	'abr-215062':ab,ti	9
#142	#138 OR #139 OR #140 OR #141	914
#143	'peginterferon beta'/exp	11
#144	'polyethylene glycol-interferon-beta-1a':ab,ti	5
#145	'peg ifn-beta-1a':ab,ti	15
#146	'polyethylene glycol-interferon beta-1a':ab,ti	5
#147	'plegridy':ab,ti	28
#148	#144 OR #145 OR #146 OR #147	48
#149	'ocrelizumab'/exp	3,314
#150	'ocrevus':ab,ti	24
#151	'r 1594':ab,ti	1
#152	'r1594':ab,ti	1
#153	'r-1594':ab,ti	1
#154	'rg-1594':ab,ti	0
#155	'pr 070769':ab,ti	0
#156	'pr070769':ab,ti	0
#157	'pr-070769':ab,ti	0
#158	#149 OR #150 OR #151 OR #152 OR #153 OR #154 OR #155 OR #156 OR #157	3315
#159	'rituximab'/exp	99,074
#160	'cd20 antibody, rituximab':ab,ti	879
#161	'rituximab cd20 antibody':ab,ti	5

#162	'mabthera':ab,ti	420
#163	'idec-c2b8 antibody':ab,ti	4
#164	'idec c2b8 antibody':ab,ti	4
#165	'idec-c2b8':ab,ti	61
#166	'idec c2b8':ab,ti	61
#167	'gp2013':ab,ti	34
#168	'rituxan':ab,ti	640
#169	#159 OR #160 OR #161 OR #162 OR #163 OR #164 OR #165 OR #166 OR #167 OR #168	99167
#170	#27 OR #42 OR #61 OR #93 OR #97 OR #118 OR #127 OR #137 OR #142 OR #148 OR #158 OR #169	490,245
#171	#12 AND #170	865
#172	'random':ab,ti OR 'control':ab,ti OR 'double-blind':ab,ti	4,373,014
#173	#171 AND #172	154

Cochrane:

Search	Query	Results
#1	(progressive multiple sclerosis):ti,ab,kw	3284
#2	(Multiple Sclerosis, Chronic Progressive):ti,ab,kw OR (Chronic Progressive Multiple Sclerosis):ti,ab,kw OR (Multiple Sclerosis, Progressive Relapsing):ti,ab,kw OR (Multiple Sclerosis, Remittent Progressive):ti,ab,kw OR (Remittent Progressive Multiple Sclerosis):ti,ab,kw	2303
#3	(Progressive Relapsing Multiple Sclerosis):ti,ab,kw OR (Multiple Sclerosis, Secondary Progressive):ti,ab,kw OR (Secondary Progressive Multiple Sclerosis):ti,ab,kw OR (Multiple Sclerosis, Primary Progressive):ti,ab,kw OR (Primary Progressive Multiple Sclerosis):ti,ab,kw	2612
#4	#1 OR #2 OR #3	3284
#5	(teriflunomide):ti,ab,kw OR (Aubagio):ti,ab,kw OR (HMR1726):ti,ab,kw OR (RS 61980):ti,ab,kw OR (HMR-1726):ti,ab,kw	368
#6	(A 771726):ti,ab,kw OR (A 1726):ti,ab,kw OR (A771726):ti,ab,kw OR (A-771726):ti,ab,kw OR (A77 1726):ti,ab,kw	352
#7	#5 OR #6	718
#8	MeSH descriptor: [Interferon-beta] explode all trees	791
#9	(Interferon beta):ti,ab,kw OR (Interferon, Fibroblast):ti,ab,kw OR (Fibroblast Interferon):ti,ab,kw OR (Interferon, beta):ti,ab,kw OR (beta Interferon):ti,ab,kw	2642
#10	(beta-Interferon):ti,ab,kw OR (Fiblaferon):ti,ab,kw OR (beta-1 Interferon):ti,ab,kw OR (beta 1 Interferon):ti,ab,kw OR (Interferon-beta1):ti,ab,kw	1688
#11	(Interferon beta1):ti,ab,kw OR (Interferon, beta-1):ti,ab,kw OR (Interferon, beta 1):ti,ab,kw	1581
#12	#8 OR #9 OR #10 OR #11	2664
#13	MeSH descriptor: [Alemtuzumab] explode all trees	154
#14	(Campath 1H):ti,ab,kw OR (Monoclonal Antibody Campath-1H):ti,ab,kw OR (Antibody Campath-1H, Monoclonal):ti,ab,kw OR (Campath-1H, Monoclonal Antibody):ti,ab,kw OR (Monoclonal Antibody Campath 1H):ti,ab,kw	68
#15	(Campath-1H):ti,ab,kw OR (Campath1H):ti,ab,kw OR (Campath 1M):ti,ab,kw OR (Campath-1M):ti,ab,kw OR (MabCambath):ti,ab,kw	69
#16	(Lemtrada):ti,ab,kw OR (Campath):ti,ab,kw OR (Campath1G):ti,ab,kw	163
#17	#13 OR #14 OR #15 OR #16	269

#18	MeSH descriptor: [Mitoxantrone] explode all trees	525
#19	(Mitozantrone):ti,ab,kw OR (DHAQ):ti,ab,kw OR (NSC287836):ti,ab,kw OR (NSC301739):ti,ab,kw OR (NSC279836):ti,ab,kw	60
#20	(NSC301739):ti,ab,kw OR (NSC301739D):ti,ab,kw OR (Mitroxone):ti,ab,kw OR (Pralifan):ti,ab,kw OR (CL232325):ti,ab,kw	0
#21	(Mitoxantrone Acetate):ti,ab,kw OR (Acetate, Mitoxantrone):ti,ab,kw OR (Mitoxantrone Hydrochloride):ti,ab,kw OR (Hydrochloride, Mitoxantrone):ti,ab,kw OR (Novantrone):ti,ab,kw	143
#22	(Ralenova):ti,ab,kw OR (Novantron):ti,ab,kw OR (Onkotrone):ti,ab,kw	60
#23	#18 OR #19 OR #20 OR #21 OR #22	654
#24	MeSH descriptor: [Natalizumab] explode all trees	97
#25	(Tysabri):ti,ab,kw OR (Antegren):ti,ab,kw	66
#26	#24 OR #25	153
#27	MeSH descriptor: [Dimethyl Fumarate] explode all trees	107
#28	(Fumarate, Dimethyl):ti,ab,kw OR (Dimethylfumarate):ti,ab,kw OR (BG00012):ti,ab,kw OR (Tecfidera):ti,ab,kw OR (FAG201):ti,ab,kw	501
#29	(Fumaderm):ti,ab,kw OR (BG12 compound):ti,ab,kw	30
#30	#27 OR #28 OR #29	510
#31	MeSH descriptor: [Fingolimod Hydrochloride] explode all trees	172
#32	(FTY720):ti,ab,kw OR (Gilenya):ti,ab,kw OR (Gilenia):ti,ab,kw	173
#33	#31 OR #32	268
#34	MeSH descriptor: [Glatiramer Acetate] explode all trees	185
#35	(Acetate, Glatiramer):ti,ab,kw OR (Copaxone):ti,ab,kw OR (Glatiramer):ti,ab,kw OR (TV5010):ti,ab,kw	731
#36	#34 OR #35	731
#37	(laquinimod):ti,ab,kw OR (ABR 215062):ti,ab,kw OR (ABR215062):ti,ab,kw OR (ABR-215062):ti,ab,kw	91
#38	(peginterferon beta):ti,ab,kw OR (polyethylene glycol-interferon-beta-1a):ti,ab,kw OR (PEG IFN-beta-1a):ti,ab,kw OR (polyethylene glycol-interferon beta-1a):ti,ab,kw OR (Plegridy):ti,ab,kw	155
#39	(ocrelizumab):ti,ab,kw OR (Ocrevus):ti,ab,kw OR (R1594):ti,ab,kw OR (RG-1594):ti,ab,kw OR (PR070769):ti,ab,kw	288
#40	MeSH descriptor: [Rituximab] explode all trees	1464
#41	(CD20 Antibody, Rituximab):ti,ab,kw OR (Rituximab CD20 Antibody):ti,ab,kw OR (Mabthera):ti,ab,kw OR (IDEC-C2B8 Antibody):ti,ab,kw OR (IDEC C2B8	1012
#42	(IDEC-C2B8):ti,ab,kw OR (IDEC C2B8):ti,ab,kw OR (GP2013):ti,ab,kw OR (Rituxan):ti,ab,kw	160
#43	#40 OR #41 OR #42	2262
#44	#7 OR #12 OR #17 OR #23 OR #26 OR #30 OR #33 OR #36 OR #37 OR #38 OR #39 OR #43	7797
#45	#4 AND #44	1193

Clinicaltrials.gov:

Search	Query	Results
#1	Status:All studies,condition or disease:Progressive Multiple Sclerosis, and other terms:Teriflunomide, Study type: interventional(Clinical Trial)	6

#2	Status:All studies,condition or disease:Progressive Multiple Sclerosis,and other terms:Interferon-beta, Study type: interventional(Clinical Trial)	5
#3	Status:All studies,condition or disease:Progressive Multiple Sclerosis,and other terms:Alemtuzumab, Study type: interventional(Clinical Trial)	3
#4	Status:All studies,condition or disease:Progressive Multiple Sclerosis,and other terms:Mitoxantrone, Study type: interventional(Clinical Trial)	2
#5	Status:All studies,condition or disease:Progressive Multiple Sclerosis,and other terms:Natalizumab, Study type: interventional(Clinical Trial)	5
#6	Status:All studies,condition or disease:Progressive Multiple Sclerosis,and other terms:Dimethyl Fumarate, Study type: interventional(Clinical Trial)	6
#7	Status:All studies,condition or disease:Progressive Multiple Sclerosis,and other terms:Fingolimod, Study type: interventional(Clinical Trial)	1
#8	Status:All studies,condition or disease:Progressive Multiple Sclerosis,and other terms:glatiramer acetate, Study type: interventional(Clinical Trial)	5
#9	Status:All studies,condition or disease:Progressive Multiple Sclerosis,and other terms:Laquinimod, Study type: interventional(Clinical Trial)	1
#10	Status:All studies,condition or disease:Progressive Multiple Sclerosis,and other terms:Peginterferon beta, Study type: interventional(Clinical Trial)	0
#11	Status:All studies,condition or disease:Progressive Multiple Sclerosis,and other terms:Ocrelizumab, Study type: interventional(Clinical Trial)	14
#12	Status:All studies,condition or disease:Progressive Multiple Sclerosis,and other terms:Rituximab, Study type: interventional(Clinical Trial)	8
**Total**		56

Table S2: Inclusion, exclusion criteria, study design and outcome assessments of the included studies

**Trails**	**Cheshmavar et al 2021**<br>**(Acta Neurologica Scandinavica)**
Inclusion Criteria	（1）a diagnosis of SPMS based on McDonald criteria in 2010;<br>（2）age between 18 and 55 years;<br>（3）expanded disability status scale (EDSS) between 0 and 5;<br>（4）diagnosis of SPMS for at least one year;<br>（5）annualized relapse rate (ARR) ≥1; and<br>（6）maintaining pregnancy prevention methods for women in reproductive ages.<br>We considered ARR ≥1 as an inclusion criterion.
Exclusion Criteria	（1）history of other demyelinating diseases of the central nervous system, autoimmune diseases, cardiac diseases (e.g. arrhythmia, angina pectoris), immunodeficiency syndromes, uncontrolled respiratory, renal, hepatic, endocrine, or gastrointestinal diseases, encephalopathy (infectious or metabolic), bone marrow transplant, whole body radiotherapy, or other treatments leading to reduction of lymphocytes, and brain and spinal cord malignancies;<br>（2）experiencing relapse within 30 days prior to intervention;<br>（3）systemic corticosteroid therapy, plasmapheresis or intravenous immunoglobulin during the last 30 days;<br>（4） active, chronic or recurrent infections (e.g. hepatitis B, hepatitis C, or syphilis);<br>（5） pregnancy or lactation;<br>（6）receiving live attenuated viral vaccines during the last 4 weeks;<br>（7）history of severe allergic reactions or anaphylaxis to monoclonal antibodies;<br>（8） history of alcohol or drug abuse during the last two years;<br>（9） not able to undergo MRI;<br>（10）white blood cell count <2500 or lymphocyte count <400.
Study Design	In RTX group patients received 1000mg intravenous RTX primarily and then every 6-months. In GA group patients received 40mg of GA 3-times/week subcutaneously.
Efficacy Outcomes	The primary outcome was comparison of EDSS between two groups. We also calculated proportional change in EDSS (defined as △-EDSS divided by the baseline EDSS) and compared it between groups.
Safety Outcomes	Included comparison of neuroimaging findings (number and location [supratentorial, infratentorial] of total and active plaques in the brain, number and location of active plaques in the cervical spine, presence of longitudinal extensive transverse myelitis [LETM], and multiple patchy lesions), ARR before and after intervention, and comparison of the number of reported side effects between two groups.

Trails	Chow et al 2021<br>(Neurology - Neuroimmunology Neuroinflammation)
Inclusion Criteria	（1）Age 18 to 65 years<br>（2）PPMS according to the McDonald (2010) and Lublin (2014) criteria<br>（3）Disease duration at least one year<br>（4）EDSS ≤ 6.5<br>（5）Written informed consent to study participation<br>（6）No other signs of significant disease judged by the investigator<br>（7）Eligible for randomization to active treatment or placebo as assessed by CSF NFL levels above 380ng/L<br>（8）Not eligible for randomization as assessed by CSF biomarker studies but accepts follow-up and open-label treatment per protocol<br>（9）Patients not eligible for randomization due to low NFL concentrations in CSF at screening can be followed up after 48 weeks, and are eligible for open-label treatment if they fulfil one of the following clinical criteria of disease progression:<br>a. 1 point increase in EDSS score from screening to week 48 if screening EDSS <6<br>b. 0.5 point increase in EDSS score from screening to week 48 if screening EDSS>5.5<br>c. 2 point increase in a physical functional system<br>d. Worsening in SDMT, 9HPT or T25FW >20% from screening to week 48
Exclusion Criteria	（1）Pregnancy or breast feeding<br>（2）Lack of effective contraception for women of child-bearing potential<br>（3）Relapse within 6 months of inclusion<br>（4）Methylprednisolone treatment within 3 months of inclusion<br>（5）Treatment with interferon-beta, glatiramer acetate, immunoglobulin G or other immunomodulatory treatment within 6 months of inclusion<br>（6）Treatment with mitoxantrone, cyclophosphamide, azathioprine or other immunosuppressive treatment within 6 months of inclusion<br>（7）Findings on the screening MRI judged to preclude participation by the treating physician<br>（8）Other diseases associated with immunodeficiency<br>（9）Other diseases judged to be relevant by the treating physician<br>（10）Anticoagulant therapy other than platelet inhibitors<br>（11）Active malignant disease in the previous 5 years<br>（12）Renal insufficiency or blood creatinine > 150 µmol/l<br>（13）Present or chronic infection with hepatitis B virus, hepatitis C virus, HIV (tested in the screening blood samples) or other infections found to be relevant by the treating physician.<br>（14）Psychiatric disorders or other disorders impairing the patient's ability to participate in the trial Contraindication to MRI<br>（15）Known allergy or hypersensitivity to dimethyl fumarate
Study Design	Fifty-four patients were randomly assigned 1:1 to receive twicedaily oral dimethyl fumarate 240 mg or placebo (Biogen, Cambridge, MA). From days 1 – 21, the study drug dose was titrated from 120 to 480 mg as a daily maintenance dose. Study drug and placebo capsules were identical and with identical packaging, labeling, expiration date, taste, and odor.
Efficacy Outcomes	The primary endpoint was change in CSF concentration of NFL from screening to week 48. Secondary endpoints were change in CSF concentrations ofmyelin basic protein (MBP), soluble B-cell maturation antigen (BCMA), chitinase 3-like 1 (CHI3L1), soluble CD27 (sCD27), soluble CD14 (sCD14), immunoglobulin G index, and albumin quotient; change in fractional anisotropy of normal-appearing white matter, magnetization transfer ratio of lesions, mean thalamic volume,

	difference in number of new and enlarged T2 lesions and percentage brain volume change; and change in EDSS, T25FW, 9HPT, and SDMT.
Safety Outcomes	Adverse events.

**Trails**	**Wolinsky et al 2020**<br>**(The Lancet Neurology)**
Inclusion Criteria	（1）Diagnosis of primary progressive multiple sclerosis (according to revised McDonald criteria)<br>（2）EDSS at screening from 3 to 6.5 points<br>（3）Disease duration from onset of MS symptoms less than (<) 15 years if EDSS greater than (>) 5.0; <10 years if EDSS greater than or equal to (>/=) 5.0<br>（4）Sexually active male and female participants of reproductive potential must use two methods of contraception throughout the study treatment phase and for 48 weeks after the last dose
Exclusion Criteria	（1）History of relapsing remitting MS, secondary progressive, or progressive relapsing MS at screening<br>（2）Inability to complete an MRI (contraindications for MRI)<br>（3）Known presence of other neurologic disorders<br>（4）Known active infection or history of or presence of recurrent or chronic infection<br>（5）History of cancer, including solid tumors and hematological malignancies (except for basal cell, in situ squamous cell carcinomas of the skin and in situ carcinoma of the cervix that have been excised and resolved)<br>（6）Previous treatment with B-cell targeted therapies (e.g. rituximab, ocrelizumab, atacicept, belimumab, or ofatumumab)<br>（7）Any previous treatment with lymphocyte trafficking blockers, with alemtuzumab, anti-cluster of differentiation 4 (CD4), cladribine, cyclophosphamide, mitoxantrone, azathioprine, mycophenolate mofetil, cyclosporine, methotrexate, total body irradiation, or bone marrow transplantation<br>（8）Any concomitant disease that may require chronic treatment with systemic corticosteroids or immunosuppressants during the course of the study
**Study Design**	At the start of the double-blind period, 732 patients were randomly assigned (2:1) to receive either 600 mg of ocrelizumab (administered as two 300 mg intravenous infusions 14 days apart; n=488) or placebo (n=244) every 24 weeks for at least 120 weeks, until a prespecified number of 253 patients had a 12-week-confirmed dis ability progression on the EDSS (ie, the primary outcome; an event-driven clinical trial design).
Efficacy Outcomes	The time to onset of CDP was defined as time from baseline to first disability progression, which is confirmed at next regularly scheduled visit >=12 weeks (>=84 days) after initial disability progression. Percent Change From Baseline in Timed 25-Foot Walk (T25-FW) at Week 120. Percent Change From Baseline in Total Volume of T2 Lesions at Week 120. Percent Change in Total Brain Volume From Week 24 to Week 120. Change in From Baseline Physical Component Summary Score (PCS) SF- 36 Health Survey (SF-36) at Week 120.
Safety Outcomes	Adverse events, discon tinuations for adverse events, serious infections, neo plasms, and deaths.

Trails	Giovannoni et al 2020 (Neurology)
Inclusion Criteria	（1）Patients must have a confirmed and documented PPMS diagnosis as defined by the 2010 Revised McDonald criteria<br>（2）Baseline magnetic resonance imaging (MRI) showing lesions consistent with PPMS in either or both brain and spinal cord<br>（3）Patients must have an Expanded Disability Status Scale (EDSS) score of 3 to 6.5, inclusive, at both screening and baseline visits<br>（4）Documented evidence of clinical disability progression in the 2 years prior to screening.<br>（5）Functional System Score (FSS) of > or equal 2 for the pyramidal system or gait impairment due to lower extremity dysfunction<br>（6）Patients must be between 25 to 55 years of age, inclusive<br>（7）Women of child-bearing potential must practice an acceptable method of birth control for 30 days before taking the study drug, and 2 acceptable methods of birth control during all study duration and until 30 days after the last dose of treatment is administered.<br>（8）Patients must sign and date a written informed consent prior to entering the study.<br>（9）Patients must be willing and able to comply with the protocol requirements for the duration of the study.
Exclusion Criteria	（1）Patients with history of any multiple sclerosis (MS) exacerbations or relapses, including any episodes of optic neuritis.<br>（2）Progressive neurological disorder other than PPMS.<br>（3）Any MRI record showing presence of cervical cord compression.<br>（4）Baseline MRI showing other findings (including lesions that are atypical for PPMS) that may explain the clinical signs and symptoms.<br>（5）Relevant history of vitamin B12 deficiency.<br>（6）Positive human T-lymphotropic virus Type I and II (HTLV-I/II) serology.<br>（7）Use of experimental or investigational drugs in a clinical study within 24 weeks prior to baseline. Use of a currently marketed drug in a clinical study within 24 weeks prior to baseline would not be exclusionary, provided no other exclusion criteria are met.<br>（8）Use of immunosuppressive agents, or cytotoxic agents, including cyclophosphamide and azathioprine within 48 weeks prior to baseline.<br>（9）Previous treatment with fingolimod (GILENYA®, Novartis), dimethyl fumarate (TECFIDERA®, Biogen Idec Inc), glatiramer acetate (COPAXONE®, Teva), interferon-β (either 1a or 1b), intravenous immunoglobulin, or plasmapheresis within 8 weeks prior to baseline.<br>（10）Use of teriflunomide (AUBAGIO®, Sanofi) within 2 years prior to baseline, except if active washout (with either cholestyramine or activated charcoal) was done 2 months or more prior to baseline.<br>（11）Prior use of monoclonal antibodies ever, except for:<br>a. natalizumab (TYSABRI®, Biogen Idec Inc), if given more than 24 weeks prior to baseline AND the patient is John Cunningham (JC) virus antibody test negative (as per medical history)<br>b. rituximab, ocrelizumab, or ofatumumab, if B cell count (CD19, as per medical history) is higher than 80 cells/µL<br>（12）Use of mitoxantrone (NOVANTRONE®, Immunex) within 5 years prior to screening. Use of mitoxantrone >5 years before screening is allowed in patients with normal ejection fraction and who did not exceed the total lifetime maximal dose.<br>（13）Previous use of laquinimod.<br>（14）Chronic (eg, more than 30 consecutive days or monthly dosing, with the intent of MS disease

	modification) systemic (intravenous, intramuscular or oral) corticosteroid treatment within 8 weeks prior to baseline.<br>（15）Previous use of cladribine or alemtuzumab (LEMTRADA®, Sanofi).<br>（16）Previous total body irradiation or total lymphoid irradiation.<br>（17）Previous stem cell treatment, cell-based treatment, or bone marrow transplantation of any kind.<br>（18）Patients who underwent endovascular treatment for chronic cerebrospinal venous insufficiency (CCSVI) within 12 weeks prior to baseline.<br>（19）Use of moderate/strong inhibitors of cytochrome P450 (CYP) 3A4 within 2 weeks prior to baseline.<br>（20）Use of inducers of CYP3A4 within 2 weeks prior to baseline.<br>（21）Pregnancy or breastfeeding.<br>（22）Serum levels ≥3× upper limit of the normal range (ULN) of either alanine aminotransferase (ALT) or aspartate aminotransferase (AST) at screening.<br>（23）Serum direct bilirubin which is ≥2×ULN at screening.<br>（24）Patients with a clinically significant or unstable medical or surgical condition that (in the opinion of the Investigator) would preclude safe and complete study participation, as determined by medical history, physical examinations, electrocardiogram (ECG), laboratory tests or chest X-ray.<br>（25）A known history of hypersensitivity to gadolinium (Gd).<br>（26）Glomerular filtration rate (GFR) < or equal 60 mL/min at screening visit.<br>（27）Inability to successfully undergo MRI scanning, including claustrophobia.<br>（28）Known drug hypersensitivity that would preclude administration of laquinimod, such as hypersensitivity to mannitol, meglumine or sodium stearyl fumarate.
Study Design	Eligible patients were randomized in a 1:1:1 ratio to receive oral laquinimod 0.6 mg or 1.5 mg or matching placebo once daily. The 1.5 mg arm was included as the maximal dose based on 2 studies that showed a laquinimod dose response up to 0.6 mg (based on MRI parameters) and a third multiple ascending dose study of up to 2.7 mg that showed no doselimiting adverse events (AEs) or laboratory findings.
Efficacy Outcomes	Percentage brain volume change (PBVC; primary endpoint) from baseline to week 48 was assessed by MRI. Secondary and exploratory endpoints included clinical and MRI measures. Efficacy endpoints were evaluated using a predefined, hierarchical statistical testing procedure.
Safety Outcomes	Adverse events, discon tinuations for adverse events, serious infections, neo plasms, and deaths.

Trails	Kappos et al 2018<br>(The Lancet Neurology)
Inclusion Criteria	（1）Prior history of relapsing remitting MS<br>（2）SPMS defined as progressive increase of disability over at least 6 months<br>（3）EDSS score of 3.0 to 6.5<br>（4）No relapse of corticosteroid treatment within 3 months
Exclusion Criteria	（1）Women of child bearing potential must use reliable forms of contraception.<br>（2）Diagnosis of Macular edema during screening period<br>（3）Any medically unstable condition determined by investigator.<br>（4）Unable to undergo MRI scans<br>（5）Hypersensitivity to any study drugs or drugs of similar class Other protocol defined<br>（6）inclusion/exclusion may apply.
Study Design	Using interactive response technology to assign numbers linked to treatment arms, patients (age 18－60 years) with SPMS and an Expanded Disability Status Scale score of 3・0－6・5 were randomly assigned (2:1) to once daily oral siponimod 2 mg or placebo for up to 3 years or until the occurrence of a prespecified number of confirmed disability progression (CDP) events.
Efficacy Outcomes	The primary endpoint was time to 3-month CDP. Secondary endpoints were: time to 6-month CDP; ARR; time to first relapse; proportion of relapse-free patients; change in score on the patient-reported 12-item Multiple Sclerosis Walking Scale; number of new or enlarging T2 lesions; number of T1 gadolinium-enhancing lesions; and percentage change in brain volume from baseline.
Safety Outcomes	Adverse events and Serious adverse events.

**Trails**	**Kapoor et al 2018**<br>**(The Lancet Neurology)**
Inclusion Criteria	（1）Ability to understand the purpose and risks of the study and provide signed and dated informed consent and authorization to use protected health information in accordance with national and local subject privacy regulations.<br>（2）SPMS defined as relapsing-remitting disease followed by progression of disability independent of or not explained by multiple sclerosis (MS) relapses for at least 2 years.<br>（3）EDSS score of 3.0 to 6.5, inclusive.<br>（4）Multiple Sclerosis Severity Score of 4 or higher.<br>（5）Documented confirmed evidence of disease progression independent of clinical relapses over the 1 year prior to enrollment as defined in the Study Reference Guide.
Exclusion Criteria	（1）Relapsing remitting multiple sclerosis (RRMS) or primary progressive MS as defined by the revised McDonald Committee criteria.<br>（2）Clinical relapse (within 3 months) prior to randomization.<br>（3）T25FW test of >30 seconds during the screening period.<br>（4）Any value below the lower limit of normal for blood levels of leukocytes, lymphocytes, or neutrophils.<br>（5）Considered by the Investigator to be immunocompromised based on medical history, physical examination, laboratory testing, or any other testing required by local guidelines, or due to prior immunosuppressive or immunomodulating treatment.<br>（6）Subjects for whom MRI is contraindicated (i.e., have pacemakers or other contraindicated implanted metal devices, are allergic to gadolinium, or have claustrophobia that cannot be medically managed).<br>（7）History of any clinically significant (as determined by the Investigator) cardiac, endocrinologic, hematologic, hepatic, immunologic, metabolic, urologic, pulmonary, neurologic (other than MS), dermatologic, psychiatric, and renal, or other major disease that would preclude participation in a clinical study.<br>（8）History of malignant disease, including solid tumors and hematologic malignancies (with the exception of basal cell and squamous cell carcinomas of the skin that have been completely excised and are considered cured).<br>（9）Known history of or positive test result for human immunodeficiency virus.<br>（10）Positive test result for hepatitis C virus (test for hepatitis C virus antibody or hepatitis B virus (test for hepatitis B surface antigen and/or hepatitis B core antibody).<br>（11）History of transplantation or any anti-rejection therapy.<br>（12）Presence of any infectious disease (e.g., cellulitis, abscess, pneumonia, septicemia) within 30 days prior to screening.<br>（13）History of progressive multifocal leukoencephalopathy or other opportunistic infections.
Study Design	In part 1, patients from 163 sites in 17 countries were randomly assigned (1:1) to receive 300 mg intravenous natalizumab or placebo every 4 weeks for 2 years. Patients were stratified by site and by EDSS score (3·0–5·5 vs 6·0–6·5). Patients completing part 1 could enrol in part 2, in which all patients received natalizumab every 4 weeks until the end of the study.
Efficacy Outcomes	The EDSS, Timed 25-Foot Walk (T25FW), and 9-Hole Peg Test (9HPT).
Safety Outcomes	The incidence of adverse events and serious adverse events.

Trails	Montalban et al 2016<br>(New England Journal of Medicine)
Inclusion Criteria	（1）Diagnosis of primary progressive multiple sclerosis (according to revised McDonald criteria)<br>（2）EDSS at screening from 3 to 6.5 points<br>（3）Disease duration from onset of MS symptoms less than (<) 15 years if EDSS greater than (>) 5.0; <10 years if EDSS greater than or equal to (>/=) 5.0<br>（4）Sexually active male and female participants of reproductive potential must use two methods of contraception throughout the study treatment phase and for 48 weeks after the last dose
Exclusion Criteria	（1）History of relapsing remitting MS, secondary progressive, or progressive relapsing MS at screening<br>（2）Inability to complete an MRI (contraindications for MRI)<br>（3）Known presence of other neurologic disorders<br>（4）Known active infection or history of or presence of recurrent or chronic infection<br>（5）History of cancer, including solid tumors and hematological malignancies (except for basal cell, in situ squamous cell carcinomas of the skin and in situ carcinoma of the cervix that have been excised and resolved)<br>（6）Previous treatment with B-cell targeted therapies (e.g. rituximab, ocrelizumab, atacicept, belimumab, or ofatumumab)<br>（7）Any previous treatment with lymphocyte trafficking blockers, with alemtuzumab, anti-cluster of differentiation 4 (CD4), cladribine, cyclophosphamide, mitoxantrone, azathioprine, mycophenolate mofetil, cyclosporine, methotrexate, total body irradiation, or bone marrow transplantation<br>（8）Any concomitant disease that may require chronic treatment with systemic corticosteroids or immunosuppressants during the course of the study
Study Design	Patients were randomly assigned in a 2:1 ratio to receive 600 mg of ocrelizumab by intravenous infusion (administered as two 300-mg infusions 14 days apart) or matching placebo every 24 weeks.
Efficacy Outcomes	The primary end point was the percentage of patients with disability progression confirmed at 12 weeks in a time-to-event analysis.secondary end points were tested in the following hierarchical order as long as each preceding end point reached a significance level of $P<0.05$: the percentage of patients with disability progression confirmed at 24 weeks in a time-to-event analysis, change in performance on the timed 25-foot walk from baseline to week 120, change in the total volume of brain lesions on T2 -weighted MRI from baseline to week 120, change in brain volume from week 24 to week 120, and change in the Physical Component Summary score of the Medical Outcomes Study 36-Item Short-Form Health Survey (SF-36), version 2, from baseline to week 120 (range, 0 to 100, with higher scores indicating better physical-health－related quality of life.
Safety Outcomes	The incidence of adverse events and serious adverse events.

Trails	Lublin et al 2016<br>(The Lancet Neurology)
Inclusion Criteria	（1）sign written informed consent prior to participating in the study.<br>（2）25 through 65 years of age inclusive.<br>（3）females of childbearing potential must:<br>a. have a negative pregnancy test at Baseline (prior to randomization).<br>b. use simultaneously two forms of effective contraception during the treatment and 3-months after discontinuation of study medication.<br>（4）diagnosis of primary progressive multiple sclerosis (according to the 2005 Revised McDonald criteria).<br>（5）time since first reported symptoms between 2 and 10 years.<br>（6）evidence of clinical disability progression in the 2 years prior to Screening.<br>（7）disability status at Screening:<br>a. EDSS score of 3.5-6.0 inclusive.<br>b. pyramidal functional system score of 2 or more.<br>c. 25'TWT less than 30 seconds.
Exclusion Criteria	（1）PPMS specific: History of relapses/attacks, Progressive neurological disorder other than PPMS, Pure cerebellar syndrome or pure visual progressive syndrome or pure, cognitive progressive syndrome, Presence of spinal cord compression at screening MRI, Relevant history of vitamin B12 deficit, Evidence of syphilis or borreliosis at Screening.<br>（2）Cardiovascular conditions: Myocardial infarction within the past 6 months or current unstable ischemic heart disease, History of angina pectoris due to coronary spasm or history of Raynaud's phenomenon, Severe cardiac failure or cardiac arrest, History of symptomatic bradycardia, Resting pulse <55 bpm pre-dose, History of sick sinus syndrome or sino-atrial heart block, History or presence of second and third degree AV block or an increase QT interval (QTc>440 ms), Arrythmia requiring treatment with class III antiarrythmic drugs, History of positive tilt test from workout of vasovagal syncope, Hypertension, not controlled with medication.<br>（3)Pulmonary: Severe respiratory disease or pulmonary fibrosis, TB, Abnormal X-ray, suggestive of active pulmonary disease, Abnormal PFT: <70% of predicted for FEV1 and FVC; <60% for DLCO, Patients receiving chronic (daily) therapies for asthma.<br>（4）Hepatic: Known history of alcohol abuse, chronic liver or biliary disease, Total or conjugated Brb >ULN, unless in context of Gilbert's syndrome, AP >1.5xULN; ALT/AST >2xULN;GT>3xULN.<br>（5）Other: History of chronic disease of the immune system other than MS, Malignancy (other than successfully treated SCC or BCC), Diabetes Mellitus, Macular Edema present at screening, HIV, Hepatitis C or B, other active infection, History of total lymphoid irradiation or bone marrow transplantation, Serum creatinine >1.7 mg/dl, WBC <3500 cells/mm3, Lymphocyte count <800 cells/mm3, History of substance abuse or any other factor that may interfere with subject ability to cooperate and comply with the study procedures, Unable to undergo MRI scans, Participation in any therapeutical clinical research study in the 6 months prior to randomization, Pregnant or lactating women.
Study Design	Patients with primary progressive multiple sclerosis recruited across 148 centres in 18 countries were randomly allocated (1:1) with computergenerated blocks to receive oral fi ngolimod or placebo for at least 36 months and a maximum of 5 years. Patients were initially assigned to ﬁ ngolimod 1・25 mg per day or placebo.
Efficacy Outcomes	Confirmed disability progression (CDP), Expanded Disability Status Scale (EDSS), the 25’ Timed-Walk Test (25’ TWT), and the Nine-Hole Peg Test (9-HPT).
Safety Outcomes	Adverse events.

Trails	Komori et al 2016<br>(Annals of Clinical and Translational Neurology)
Inclusion Criteria	（1）MS as defined by the modified McDonald s criteria (Polman, Reingold et al. 2005).<br>（2）SP-MS as documented by lack of MS relapse for the past 1 year and non-remitting/sustained (> 3 months) progression of disability.<br>（3）Age 18-65, inclusive, at the time of the first screening baseline visit.<br>（4）EDSS 3.0 to 7.0, inclusive, at the time of the first screening baseline visit.<br>（5）Able to provide informed consent.<br>（6）Willing to participate in all aspects of trial design and follow-up.<br>（7）Lack of CEL on all MRIs performed within the last 12 months or if patient has CEL, then documentation that they tried and failed or could not tolerate FDA approved disease modifying therapies (DMTh).<br>（8）Not receiving any DMTh (such as IFN-beta preparation, glatiramer acetate, corticosteroid, natalizumab, fingolimod, immunosuppressive agents or experimental therapeutics) for a period of at least 1 month before enrollment in the study, allowing for at least a 1-year<br>period off therapy prior to the first study dose.<br>（9）Agreeing to commit to the use of a reliable/accepted method of birth control (i.e. hormonal contraception (birth control pills, injected hormones, vaginal ring), intrauterine device, barrier methods with spermicide (diaphragm with spermicide, condom with spermicide) or they have undergone surgical sterilization (such as hysterectomy, tubal ligation, or vasectomy)) during enrollment in the study and through 12 months after the last dose of study drug.
Exclusion Criteria	（1）RR-MS or PP-MS<br>（2）Evidence of clearly documented MS relapse within the last 1 year<br>（3）Alternative diagnoses that can explain neurological disability and MRI findings<br>（4）Clinically significant medical disorders that, in the judgment of the investigators could cause CNS tissue damage, limit its repair, expose the patient to undue risk of harm or prevent the patient from completing the study (such as, but not limited to cerebrovascular disease, ischemic cardiomyopathy, clotting disorder, brittle diabetes, neurodegenerative disorder)<br>（5）Pregnant or breastfeeding female<br>（6）History or sign of congenital or acquired immunodeficiency or chronic infections, such as HIV/AIDS, Hepatitis A, B or C, HTLV-1 carrier and others that would expose patient to risks of pathogen reactivation associated with rituximab treatment<br>（7）Abnormal screening/baseline blood tests exceeding any of the limits defined below:<br>a. Serum alanine transaminase or aspartate transaminase levels which are greater than three times the upper limit of normal values.<br>b. Total white blood cell count < 3 000/mm(3)<br>c. Platelet count < 85 000/mm(3)<br>d. Serum creatinine level > 2.0 mg/dl and eGFR (glomerular filtration rate) < 60<br>e. Serological evidence of HIV, HTLV-1 or active hepatitis A, B or C<br>f. Positive pregnancy test<br>g. Positive CSF or serum quantitative PCR for JC virus on CSF collected from the baseline spinal tap (test will be performed by CLIA certified laboratory of Gene Major, NINDS)<br>h. Total serum IgG < 600mg/dl (nl 642-1730mg/dl) or total serum IgM < 30mg/dl (nl 34-342mg/dl) as these Ig deficiencies would suggest underlying abnormalities with B cell function/maturation

Study Design	After premedication with 100 mg IV methylprednisolone, 50 mg of diphenhydramine, 650 mg of acetaminophen, and 1 mg of lorazepam, 20 cc of CSF was withdrawn by lumbar puncture (LP) followed by injection of rituximab (25 mg; 1:1 dilution in normal saline [NS]) or placebo (only NS) over 2 min, followed by 8 cc of NS flush (Mo 0). Patients were observed for 4 h in Trendelenburg position (to facilitate the flow of CSF from lumbar cistern toward hemispheres) before intravenous rituximab (200 mg) or placebo infusion was initiated. The second intravenous dose of rituximab or placebo (Mo 0.5), the second (Mo 1.5) and third (Mo 12) intrathecal rituximab or placebo doses were administered using analogous procedures.
Efficacy Outcomes	The clinical evaluations were done every 6 months with EDSS,13 Scripps Neurological Rating Scale (NRS14), and MS functional Composite Scale (MSFC15). Neuroimaging evaluation was performed every 6 months with routine spin-echo and gradient-echo T1-weighted images were collected following intravenous administration of 0.1 mmol/kg gadopentetate dimeglumine as described.
Safety Outcomes	CSF collection, processing, and immunophenotyping. Measurement of rituximab concentration and other biomarkers. In vitro B-cell surface and intracellular rituximab saturation assay. In vitro NK cell cytotoxicity assay. In vitro B-cell cytotoxicity assay.

Trails	Montalban et al 2009<br>(Multiple Sclerosis Journal)
Inclusion Criteria	(1) age from 18 to 65 years;<br>(2) MS according to Schumacher criteria20 for at least 1 year;<br>(3) PPMS or transitional MS; in the case of PPMS, the diagnosis should be made using the Schumacher criteria20 with a clinical course showing slow or stepped up progression with signs and symptoms lasting at least 12 months;<br>(4) EDSS score of between 3.0 and 7.0;<br>(5) transitional MS was defined as those patients with a progressive course and a single relapse before or during progression;<br>(6) use of an adequate contraceptive method.
Exclusion Criteria	(1) other forms of MS;<br>(2) any other disease more likely to be the cause of the patient signs and symptoms not adequately excluded by means of spinal cord MR and other ancillary tests when deemed necessary;<br>(3) any other disabling or medically unstable condition which might interfere with clinical or MR evaluation and study compliance or data interpretation;<br>(4) pregnancy or nursing;<br>(5) previous treatment with immunosuppressive/ immunomodulatory drugs;<br>(6) intolerance to paracetamol and to non-steroidal anti-inflammatory agents;<br>(7) abuse of alcohol or other substances within 90 days prior to the examination visit;<br>(8) history of attempted suicide or current suicidal ideation.
Study Design	This was a double-blind, stratified, randomized, phase II pilot study with two parallel groups: IFNbeta-1b at doses of 8 MIU on alternate days or placebo. The double-blind treatment phase lasted for 24 months, followed by a 90-day follow-up period. Patients were stratified into PPMS or transitional MS and randomized (using a randomization list) into blocks of six, and for each block treatment was assigned in a one-to-one ratio. After randomization and from day 1 of the study, patients received 0.5 ml (4 MIU) of the study product on alternate days for two weeks (14 days). From day 15 of the study (8th dose), this dose was increased to 1.0 ml (8 MIU) of the product for the rest of the treatment phase in order to improve treatment tolerance. During the first six weeks of the study (day 1 to day 42), patients received concomitant treatment with oral prednisone, at doses of 60 mg/day for the first week, after which the daily dose was decreased by 10mg in each subsequent week.
Efficacy Outcomes	The primary efficacy variable was the time to neurological deterioration confirmed on two consecutive visits (3 months). Secondary efficacy variables were: time to neurological deterioration confirmed at 6 months; proportion of patients at 24 months with a neurological deterioration confi rmed at 3 and 6 months; mean EDSS change along the study; mean change in the 25-feet Timed Walk Test (TWT), Nine Hole Peg Test (NHPT), PASAT, and MSFC along the study; change during the study in the score of the following assessments: BRB-N tests, Global Evaluation of MS, SIP, and the fatigue severity scale. A number of MR exploratory secondary efficacy variables were also investigated: change in T1 and T2 lesion volume, number of active (new or enlarging) T2 lesions, proportion of patients/scans with active T2 lesions, and also atrophy and MTR parameters.
Safety Outcomes	Adverse events.

**Trails**	**Hawker et al 2009**<br>**(Annals of Neurology)**
Inclusion Criteria	Patients 18 to 65 years old with a PPMS diagnosis18; disease duration of $\geq$ 1 year; EDSS baseline between 2.0 and 6.5 points, inclusive; Functional Systems scale score of $\geq$ 2.0 for the pyramidal system or gait impairment due to lower extremity dysfunction; and presence of IgG oligoclonal bands or elevated CSF IgG index, or both, from CSF specimen obtained at screening or documented during the previous 24 months.
Exclusion Criteria	History of MS exacerbation or neuromyelitis optica; history of myelopathy or neurodegenerative central nervous system conditions; systemic autoimmune disorders; recurrent or chronic infections; recent treatment with immunomodulating or immunosuppressant therapies; and metabolic, hematologic, or immunologic laboratory abnormalities.
Study Design	Using 2:1 randomization, 439 PPMS patients received two 1,000mg intravenous rituximab or placebo infusions every 24 weeks, through 96 weeks (4 courses).
Efficacy Outcomes	The primary endpoint was time to confirmed disease progression (CDP), a prespecified increase in Expanded Disability Status Scale sustained for 12 weeks. Secondary endpoints were change from baseline to week 96 in T2 lesion volume and total brain volume on magnetic resonance imaging scans.
Safety Outcomes	Adverse events and death.

Trails	Wolinsky et al 2007<br>(Annals of Neurology)
Inclusion Criteria	Eligible patients were between 30 and 65 years of age with an entry EDSS14 score of 3.0 to 6.5 inclusive. The diagnosis of PPMS was confirmed by the principal investigator at each study site, and those with a history of any relapses were specifically excluded. All patients were required to have progressive neurological symptoms including evidence of myelopathy for at least 6 months before the screening visit, with objective evidence of pyramidal damage on neurological examination, including a Functional System (FS) score for the pyramidal system of 2 or greater. All patients were to have evidence of multilevel (disseminated) central nervous system disease based on objective evidence from neurological examination alone or supplemented by findings on MRI or visual- or auditory-evoked responses.
Exclusion Criteria	Patients were ineligible if they had lymphopenia level less than 3,000 cells/ml; had used an interferon-␤ drug, immunosuppressant, immunomodulating agent, corticosteroid, or investigational drug within 3 months of study entry; had any other known life-threatening, clinically significant, or uncontrolled illness; were allergic to gadolinium or had any condition that would preclude MRI; or if they were pregnant or lactating.
Study Design	An approved amendment to the original protocol allowed all reconsenting subjects who completed the 3-year, double-blind treatment period to continue in a blinded extension trial whereas remaining on their assigned treatment with GA 20mg or PBO (2:1 assignment ratio) until the last enrolled patient had completed 3 years of daily subcutaneous treatment (up to 53 months for the first patient enrolled).
Efficacy Outcomes	The primary end point was an intention-to-treat analysis of time to 1- (entry expanded disability status scale, 3.0 – 5.0) or 0.5-point expanded disability status scale change (entry expanded disability status scale, 5.5 – 6.5) sustained for 3 months. Secondary and exploratory end points included proportion of progression-free patients, changes from baseline in mean EDSS scores and mean MSFC scores, number and volume of brain lesions defined by FLAIR on MRI, number of gadopentetate dimeglumine (Gd) – enhanced lesions, volume of T1-hypointense lesions (black holes) as a percentage of FLAIR-defined lesion burden, and brain volume loss. Safety was assessed by adverse event reporting, vital signs, electrocardiograms, and laboratory tests.
Safety Outcomes	Adverse events and death.

**Trails**	**Andersen et al 2004**<br>**(J Neurol Neurosurg Psychiatry)**
Inclusion Criteria	Patients aged 18–65 years were eligible for inclusion in this trial if they had a diagnosis of clinically definite MS17 for at least 1 year, and which was classified as SPMS with an EDSS score below 7.0.
Exclusion Criteria	（1）interferon, immunosuppressant, or chronic steroid therapy within the previous 3 months,<br>（2）pregnancy or lactation,<br>（3）seizure within the previous 3 months, and 4) a history of severe depression.
Study Design	A total of 371 patients with clinically definite SPMS were randomised to receive either placebo or subcutaneous IFN beta-1a, 22 mg once weekly, for 3 years. Clinical assessments were performed every 6 months.
Efficacy Outcomes	The primary outcome was time to sustained disability, as defined by time to first confirmed 1.0 point increase on the Expanded Disability Status Scale (EDSS). Secondary outcomes included a sensitive disability measure and relapse rate.
Safety Outcomes	Adverse events.

Trails	Leary et al 2003<br>(NEUROLOGY)
Inclusion Criteria	（1）PPMS of at least 2 years’ duration,<br>（2）aged 18 to 60 years, and<br>（3） Expanded Disability Status Scale (EDSS)18 score of 2.0 to 7.0 inclusive.
Exclusion Criteria	（1）interferon, immunosuppressant, or chronic steroid therapy within the previous 3 months,<br>（2）pregnancy or lactation,<br>（3） seizure within the previous 3 months, and 4) a history of severe depression.
Study Design	The authors report a randomized, controlled trial restricted to primary progressive MS. Methods: Fifty subjects were randomized to weekly IM interferon beta-1a 30 µg, 60 µg, or placebo for 2 years.
Efficacy Outcomes	The primary endpoint was time to sustained progression in disability. Secondary outcomes included the timed 10-meter walk, nine-hole peg test, and on MRI, T2 and T1 brain lesion loads and brain and spinal cord atrophy. Results: The 30-µg dose of interferonbeta-1a was well tolerated, but the 60-µg dose caused severe flulike reactions and raised liver enzymes.
Safety Outcomes	Adverse events and death.

Trails	Hartung et al 2002<br>(The Lancet Neurology)
Inclusion Criteria	age 18 – 55 years; stepwise progression of disability between clinical relapses (progressive relapsing multiple sclerosis, also termed worsening relapsing – remitting multiple sclerosis) or gradual progression of disability with or without superimposed clinical relapses (secondary progressive multiple sclerosis); score on the Kurtzke EDSS9 of 3 • 0 – 6 • 0; worsening of 1 • 0 or more EDSS points during the 18 months before enrolment; no clinical relapse or treatment with glucocorticosteroids for at least 8 weeks before enrolment; no previous treatment with mitoxantrone, interferons, glatiramer acetate, cytotoxic drugs, or total-body lymphoid irradiation; left-ventricular ejection fraction greater than 50%; and values within the normal range for white-blood-cell count (more than $4*10^9$/L), neutrophil count (more than $2*10^9$/L), and platelet count (more than $100*10^9$/L).
Exclusion Criteria	
Study Design	194 patients with worsening relapsing – remitting or secondary progressive multiple sclerosis were assigned placebo or mitoxantrone (5 mg/m2 [exploratory group] or 12 mg/m2 intravenously) every 3 months for 24 months. Clinical assessments were made every 3 months for 24 months.
Efficacy Outcomes	change from baseline EDSS at 24 months, change from baseline ambulation index at 24 months, number of relapses treated with corticosteroids, time to first treated relapse, and change from baseline standardised neurological status at 24 months. Secondary endpoints included the proportions of patients with deterioration of at least 1 EDSS point, proportion of patients with such EDSS deterioration confirmed after 3 months and 6 months, time to first sustained EDSS deterioration, time to first relapse, number and annual rate of relapses, proportion of patients without relapse, number of days in hospital, use of wheelchair assistance, and quality of life assessed by the Stanford health assessment questionnaire.
Safety Outcomes	Adverse events.

Trails	Francis et al 2001<br>(NEUROLOGY)
Inclusion Criteria	Eligible patients had clinically definite SPMS, defined as progressive deterioration of disability for at least 6 months with an increase of at least 1 EDSS point over the last 2 years (or 0.5 point between EDSS score of 6.0 and 6.5), with or without superimposed exacerbations, following an initial RR course. At study entry, patients were between 18 and 55 years old, with EDSS scores from 3.0 to 6.5 and pyramidal functional score of at least 2.
Exclusion Criteria	Immunosuppressive or immunomodulatory treatments during the previous 3 to 12 months depending on the drug, prior treatment with interferon or total lymphoid irradiation, corticosteroid use or a disease exacerbation in the previous 8 weeks, severe concurrent illness, and pregnancy or lactation. Potentially fertile women were required to use effective contraception.
Study Design	A total of 618 patients received subcutaneous placebo or interferon beta-1a, 22 or 44µg three times weekly for 3 years. Patients were assessed every 3 months.
Efficacy Outcomes	The primary efficacy outcome was time to confirmed progression in disability, Secondary clinical outcomes (with corresponding regression methods) included proportion of patients progressing (logistic), exacerbation count (Poisson), time to first exacerbation (Cox PH), time between first and second exacerbations (Cox PH), number of moderate and severe exacerbations (Poisson), number of steroid courses for MS (Poisson), number of hospitalizations for MS (Poisson), and Integrated Disability Status Score (IDSS, defined by area under an EDSS time-curve adjusted for baseline19 ; analysis of variance on ranks).
Safety Outcomes	Adverse events.

Trails	Miller et al 1999<br>(Annals of Neurology)
Inclusion Criteria	SPMS was defined as having had a previous relapsing – remitting course, but subsequently entering a phase of gradual clinical progression, with or without superimposed relapses, for at least 6 months. Entry Expanded Disability Status Scale (EDSS) score was 3.0 to 6.5, inclusive, and patients were required to have had either two relapses or a 1-point increase in the EDSS during the 2 years before entering the study.
Exclusion Criteria	NR
Study Design	A subgroup of 125 patients also underwent monthly gadolinium-enhanced and proton density/T2-weighted brain MRI from months 0 to 6 and 18 to 24 to determine the effect of treatment on the frequency of new lesion activity, defined as new enhancing lesions and new/enlarging T2 lesions not enhancing with gadolinium. All patients were randomized to treatment with either placebo or IFN-beta 1b (Betaseron), 8 million IU, subcutaneously on alternate days.
Efficacy Outcomes	The primary outcome was time to a 1-point increase in the EDSS (or a 0.5 increase if the baseline EDSS was 6 or 6.5) confirmed at two consecutive assessments at least 3 months apart.
Safety Outcomes	Nothing.

Trails	Kappos et al 1998<br>(The Lancet Neurology)
Inclusion Criteria	Had a clinically or laboratory supported definite diagnosis of MS. Secondary progression was defined as a period of deterioration, independent of relapses, sustained for at least 6 months, and that followed a period of relapsing-remitting MS. Superimposed relapses were allowed. Patients were aged 18 – 55 years, with a baseline EDSS score of 3 •0 – 6 •5 inclusive and a recorded history of either two relapses or more or 1 • 0 point or more increase in EDSS in the previous 2 years. Immunosuppressive or immunomodulatory treatment and other putative treatments for MS were not permitted for defined periods before entry into the study.
Exclusion Criteria	intolerable adverse events or clinically relevant laboratory deviations, pregnancy, use of prohibited medication, or if the code was broken.
Study Design	Outpatients with SP-MS having scores of 3 •0 – 6 •5 on the Expanded Disability Status Scale (EDSS) received either 8 million IU interferon -1b every other day subcutaneously, or placebo, for up to 3 years.
Efficacy Outcomes	Functional system and EDSS scores. The functional-system scores measure unction within individual neurological systems including visual, pyramidal, cerebellar, brainstem, sensory, bowel and bladder, cerebral (mental), and other functions.
Safety Outcomes	Adverse events.

PLA: placebo; PPMS: primary progressive multiple sclerosis; SPMS: secondary-progressive multiple sclerosis; NR: not reported; EDSS (Expanded Disability Status Scale); CDP; 9HPT (9-Hole Peg Test, s); T25FW (Timed 25-Foot Walk, s); AEs (Adverse Events); SAEs (Serious Adverse Events).

Table S3: Detailed certainty of evidence for each outcome in league table.

Comparison	Number of studies	Within-study bias	Reporting bias	Indirectness	Imprecision	Heterogeneity	Incoherence	Confidence rating	Reason(s) for downgrading
**1. EDSS**									
C_Rituximab:I_Glatiramer-acetate	1	No concerns	Low risk	No concerns	No concerns	Major concerns	No concerns	High	[]
C_Rituximab:L_Placebo	2	No concerns	Low risk	No concerns	No concerns	Major concerns	No concerns	High	[]
G_Interferon-beta 1b:L_Placebo	1	No concerns	Low risk	No concerns	No concerns	Major concerns	No concerns	High	[]
I_Glatiramer-acetate:L_Placebo	1	No concerns	Low risk	No concerns	No concerns	Major concerns	No concerns	High	[]
J_Mitoxantrone:L_Placebo	1	No concerns	Low risk	No concerns	No concerns	Major concerns	No concerns	High	[]
K_Dimethyl Fumarate:L_Placebo	1	No concerns	Low risk	No concerns	Some concerns	Some concerns	No concerns	High	[]
C_Rituximab:G_Interferon-beta 1b	0	No concerns	Low risk	No concerns	No concerns	Major concerns	No concerns	High	[]
C_Rituximab:J_Mitoxantrone	0	No concerns	Low risk	No concerns	No concerns	Major concerns	No concerns	High	[]
C_Rituximab:K_Dimethyl Fumarate	0	No concerns	Low risk	No concerns	Some concerns	Some concerns	No concerns	High	[]
G_Interferon-beta 1b:I_Glatiramer-acetate	0	No concerns	Low risk	No concerns	No concerns	Major concerns	No concerns	High	[]
G_Interferon-beta 1b:J_Mitoxantrone	0	No concerns	Low risk	No concerns	No concerns	Major concerns	No concerns	High	[]
G_Interferon-beta 1b:K_Dimethyl Fumarate	0	No concerns	Low risk	No concerns	Some concerns	Some concerns	No concerns	High	[]
I_Glatiramer-acetate:J_Mitoxantrone	0	No concerns	Low risk	No concerns	No concerns	Major concerns	No concerns	High	[]
I_Glatiramer-acetate:K_Dimethyl Fumarate	0	No concerns	Low risk	No concerns	Some concerns	Some concerns	No concerns	High	[]
J_Mitoxantrone:K_Dimethyl Fumarate	0	No concerns	Low risk	No concerns	No concerns	Major concerns	No concerns	High	[]
C_Rituximab:I_Glatiramer-acetate	1	No concerns	Low risk	No concerns	No concerns	Major concerns	No concerns	High	[]
C_Rituximab:L_Placebo	2	No concerns	Low risk	No concerns	No concerns	Major concerns	No concerns	High	[]
G_Interferon-beta 1b:L_Placebo	1	No concerns	Low risk	No concerns	No concerns	Major concerns	No concerns	High	[]
I_Glatiramer-acetate:L_Placebo	1	No concerns	Low risk	No concerns	No concerns	Major concerns	No concerns	High	[]
J_Mitoxantrone:L_Placebo	1	No concerns	Low risk	No concerns	No concerns	Major concerns	No concerns	High	[]
K_Dimethyl Fumarate:L_Placebo	1	No concerns	Low risk	No concerns	Some concerns	Some concerns	No concerns	High	[]
C_Rituximab:G_Interferon-beta 1b	0	No concerns	Low risk	No concerns	No concerns	Major concerns	No concerns	High	[]
C_Rituximab:J_Mitoxantrone	0	No concerns	Low risk	No concerns	No concerns	Major concerns	No concerns	High	[]
C_Rituximab:K_Dimethyl Fumarate	0	No concerns	Low risk	No concerns	Some concerns	Some concerns	No concerns	High	[]
G_Interferon-beta 1b:I_Glatiramer-acetate	0	No concerns	Low risk	No concerns	No concerns	Major concerns	No concerns	High	[]
**2.CDP**									

A_Ocrelizumab:L_Placebo	2	No concerns	Low risk	No concerns	No concerns	Major concerns	Major concerns	High	[]
B_Natalizumab:L_Placebo	1	No concerns	Low risk	No concerns	No concerns	Major concerns	Major concerns	High	[]
C_Rituximab:L_Placebo	1	No concerns	Low risk	No concerns	Major concerns	No concerns	Major concerns	High	[]
D_Laquinimod:L_Placebo	1	No concerns	Low risk	No concerns	Some concerns	Some concerns	Major concerns	High	[]
E_Siponimod:L_Placebo	1	No concerns	Low risk	No concerns	No concerns	Major concerns	Major concerns	High	[]
F_Fingolimod:L_Placebo	1	No concerns	Low risk	No concerns	No concerns	Major concerns	Major concerns	High	[]
G_Interferon-beta 1b:L_Placebo	2	No concerns	Low risk	No concerns	No concerns	Major concerns	Major concerns	High	[]
H_Interferon-beta 1a:L_Placebo	1	No concerns	Low risk	No concerns	Some concerns	Some concerns	Major concerns	High	[]
I_Glatiramer acetate:L_Placebo	1	No concerns	Low risk	No concerns	No concerns	Major concerns	Major concerns	High	[]
J_Mitoxantrone:L_Placebo	1	No concerns	Low risk	No concerns	Some concerns	Some concerns	Major concerns	High	[]
A_Ocrelizumab:B_Natalizumab	0	No concerns	Low risk	No concerns	Some concerns	Some concerns	Major concerns	High	[]
A_Ocrelizumab:C_Rituximab	0	No concerns	Low risk	No concerns	Some concerns	Some concerns	Major concerns	High	[]
A_Ocrelizumab:D_Laquinimod	0	No concerns	Low risk	No concerns	Major concerns	No concerns	Major concerns	High	[]
A_Ocrelizumab:E_Siponimod	0	No concerns	Low risk	No concerns	Major concerns	No concerns	Major concerns	High	[]
A_Ocrelizumab:F_Fingolimod	0	No concerns	Low risk	No concerns	Some concerns	Some concerns	Major concerns	High	[]
A_Ocrelizumab:G_Interferon-beta 1b	0	No concerns	Low risk	No concerns	Some concerns	Some concerns	Major concerns	High	[]
A_Ocrelizumab:H_Interferon-beta 1a	0	No concerns	Low risk	No concerns	Some concerns	Some concerns	Major concerns	High	[]
A_Ocrelizumab:I_Glatiramer acetate	0	No concerns	Low risk	No concerns	No concerns	Major concerns	Major concerns	High	[]
A_Ocrelizumab:J_Mitoxantrone	0	No concerns	Low risk	No concerns	Major concerns	No concerns	Major concerns	High	[]
B_Natalizumab:C_Rituximab	0	No concerns	Low risk	No concerns	Major concerns	No concerns	Major concerns	High	[]
B_Natalizumab:D_Laquinimod	0	No concerns	Low risk	No concerns	Major concerns	No concerns	Major concerns	High	[]
B_Natalizumab:E_Siponimod	0	No concerns	Low risk	No concerns	Some concerns	Some concerns	Major concerns	High	[]
B_Natalizumab:F_Fingolimod	0	No concerns	Low risk	No concerns	No concerns	Major concerns	Major concerns	High	[]
B_Natalizumab:G_Interferon-beta 1b	0	No concerns	Low risk	No concerns	Some concerns	Some concerns	Major concerns	High	[]
B_Natalizumab:H_Interferon-beta 1a	0	No concerns	Low risk	No concerns	Major concerns	No concerns	Major concerns	High	[]
B_Natalizumab:I_Glatiramer acetate	0	No concerns	Low risk	No concerns	Some concerns	Some concerns	Major concerns	High	[]
B_Natalizumab:J_Mitoxantrone	0	No concerns	Low risk	No concerns	Some concerns	Some concerns	Major concerns	High	[]
C_Rituximab:D_Laquinimod	0	No concerns	Low risk	No concerns	Major concerns	No concerns	Major concerns	High	[]
C_Rituximab:E_Siponimod	0	No concerns	Low risk	No concerns	Some concerns	Some concerns	Major concerns	High	[]
C_Rituximab:F_Fingolimod	0	No concerns	Low risk	No concerns	Major concerns	No concerns	Major concerns	High	[]

C_Rituximab:G_Interferon-beta 1b	0	No concerns	Low risk	No concerns	Some concerns	Some concerns	Major concerns	High	[]
C_Rituximab:H_Interferon-beta 1a	0	No concerns	Low risk	No concerns	Major concerns	No concerns	Major concerns	High	[]
C_Rituximab:I_Glatiramer acetate	0	No concerns	Low risk	No concerns	Major concerns	No concerns	Major concerns	High	[]
C_Rituximab:J_Mitoxantrone	0	No concerns	Low risk	No concerns	Some concerns	Some concerns	Major concerns	High	[]
D_Laquinimod:E_Siponimod	0	No concerns	Low risk	No concerns	Major concerns	No concerns	Major concerns	High	[]
D_Laquinimod:F_Fingolimod	0	No concerns	Low risk	No concerns	Major concerns	No concerns	Major concerns	High	[]
D_Laquinimod:G_Interferon-beta 1b	0	No concerns	Low risk	No concerns	Major concerns	No concerns	Major concerns	High	[]
D_Laquinimod:H_Interferon-beta 1a	0	No concerns	Low risk	No concerns	Major concerns	No concerns	Major concerns	High	[]
D_Laquinimod:I_Glatiramer acetate	0	No concerns	Low risk	No concerns	Some concerns	Some concerns	Major concerns	High	[]
D_Laquinimod:J_Mitoxantrone	0	No concerns	Low risk	No concerns	Major concerns	No concerns	Major concerns	High	[]
E_Siponimod:F_Fingolimod	0	No concerns	Low risk	No concerns	Some concerns	Some concerns	Major concerns	High	[]
E_Siponimod:G_Interferon-beta 1b	0	No concerns	Low risk	No concerns	Some concerns	Some concerns	Major concerns	High	[]
E_Siponimod:H_Interferon-beta 1a	0	No concerns	Low risk	No concerns	Some concerns	Some concerns	Major concerns	High	[]
E_Siponimod:I_Glatiramer acetate	0	No concerns	Low risk	No concerns	No concerns	Major concerns	Major concerns	High	[]
E_Siponimod:J_Mitoxantrone	0	No concerns	Low risk	No concerns	Major concerns	No concerns	Major concerns	High	[]
F_Fingolimod:G_Interferon-beta 1b	0	No concerns	Low risk	No concerns	Some concerns	Some concerns	Major concerns	High	[]
F_Fingolimod:H_Interferon-beta 1a	0	No concerns	Low risk	No concerns	Major concerns	No concerns	Major concerns	High	[]
F_Fingolimod:I_Glatiramer acetate	0	No concerns	Low risk	No concerns	Some concerns	Some concerns	Major concerns	High	[]
F_Fingolimod:J_Mitoxantrone	0	No concerns	Low risk	No concerns	Some concerns	Some concerns	Major concerns	High	[]
G_Interferon-beta 1b:H_Interferon-beta 1a	0	No concerns	Low risk	No concerns	Some concerns	Some concerns	Major concerns	High	[]
G_Interferon-beta 1b:I_Glatiramer acetate	0	No concerns	Low risk	No concerns	No concerns	Major concerns	Major concerns	High	[]
G_Interferon-beta 1b:J_Mitoxantrone	0	No concerns	Low risk	No concerns	Some concerns	Some concerns	Major concerns	High	[]
H_Interferon-beta 1a:I_Glatiramer acetate	0	No concerns	Low risk	No concerns	Some concerns	Some concerns	Major concerns	High	[]
H_Interferon-beta 1a:J_Mitoxantrone	0	No concerns	Low risk	No concerns	Some concerns	Some concerns	Major concerns	High	[]
I_Glatiramer acetate:J_Mitoxantrone	0	No concerns	Low risk	No concerns	Some concerns	Some concerns	Major concerns	High	[]
**3. T25FW**									
A_Ocrelizumab:L_Placebo	1	No concerns	Low risk	No concerns	Some concerns	Some concerns	Major concerns	High	[]
B_Natalizumab:L_Placebo	1	No concerns	Low risk	No concerns	No concerns	Major concerns	Major concerns	High	[]
E_Siponimod:L_Placebo	1	No concerns	Low risk	No concerns	No concerns	Major concerns	Major concerns	High	[]
F_Fingolimod:L_Placebo	1	No concerns	Low risk	No concerns	No concerns	Major concerns	Major concerns	High	[]

A_Ocrelizumab:B_Natalizumab	0	No concerns	Low risk	No concerns	Some concerns	Some concerns	Major concerns	High	[]
A_Ocrelizumab:E_Siponimod	0	No concerns	Low risk	No concerns	Some concerns	Some concerns	Major concerns	High	[]
A_Ocrelizumab:F_Fingolimod	0	No concerns	Low risk	No concerns	Some concerns	Some concerns	Major concerns	High	[]
B_Natalizumab:E_Siponimod	0	No concerns	Low risk	No concerns	Some concerns	Some concerns	Major concerns	High	[]
B_Natalizumab:F_Fingolimod	0	No concerns	Low risk	No concerns	Some concerns	Some concerns	Major concerns	High	[]
E_Siponimod:F_Fingolimod	0	No concerns	Low risk	No concerns	No concerns	Major concerns	Major concerns	High	[]
**4. 9HPT**									
A_Ocrelizumab:L_Placebo	1	No concerns	Low risk	No concerns	No concerns	Major concerns	No concerns	Low	["Heterogeneity"]
B_Natalizumab:L_Placebo	1	No concerns	Low risk	No concerns	No concerns	Major concerns	No concerns	Low	["Heterogeneity"]
F_Fingolimod:L_Placebo	1	No concerns	Low risk	No concerns	Some concerns	Some concerns	No concerns	Low	["Imprecision","Heterogeneity"]
A_Ocrelizumab:B_Natalizumab	0	No concerns	Low risk	No concerns	Major concerns	No concerns	No concerns	Low	["Imprecision"]
A_Ocrelizumab:F_Fingolimod	0	No concerns	Low risk	No concerns	Some concerns	Some concerns	No concerns	Low	["Imprecision","Heterogeneity"]
B_Natalizumab:F_Fingolimod	0	No concerns	Low risk	No concerns	No concerns	Major concerns	No concerns	Low	["Heterogeneity"]
**5. New or enlarging T2 lesions**									
F_Fingolimod:L_Placebo	1	No concerns	Low risk	No concerns	No concerns	Major concerns	Major concerns	High	[]
G_Interferon-beta 1b:L_Placebo	1	Some concerns	Low risk	No concerns	No concerns	Major concerns	Major concerns	High	[]
K_Dimethyl Fumarate:L_Placebo	1	No concerns	Low risk	No concerns	Major concerns	No concerns	Major concerns	High	[]
F_Fingolimod:G_Interferon-beta 1b	0	No concerns	Low risk	No concerns	No concerns	Major concerns	Major concerns	High	[]
F_Fingolimod:K_Dimethyl Fumarate	0	No concerns	Low risk	No concerns	Major concerns	No concerns	Major concerns	High	[]
G_Interferon-beta 1b:K_Dimethyl Fumarate	0	No concerns	Low risk	No concerns	Major concerns	No concerns	Major concerns	High	[]
**6. Change from baseline in total volume of lesions on T2-weighted images (mm^3)**									
A_Ocrelizumab:L_Placebo	1	No concerns	Low risk	No concerns	--	--	--	High	[]
C_Rituximab:L_Placebo	1	No concerns	Low risk	No concerns	--	--	--	High	[]
E_Siponimod:L_Placebo	1	No concerns	Low risk	No concerns	--	--	--	High	[]
A_Ocrelizumab:C_Rituximab	0	No concerns	Low risk	No concerns	--	--	--	High	[]
A_Ocrelizumab:E_Siponimod	0	No concerns	Low risk	No concerns	--	--	--	High	[]
C_Rituximab:E_Siponimod	0	No concerns	Low risk	No concerns	--	--	--	High	[]
**7. AEs**									
A_Ocrelizumab:L_Placebo	1	No concerns	Low risk	No concerns	No concerns	Major concerns	Major concerns	High	[]
B_Natalizumab:L_Placebo	1	No concerns	Low risk	No concerns	No concerns	Major concerns	Major concerns	High	[]

C_Rituximab:L_Placebo	1	No concerns	Low risk	No concerns	No concerns	Major concerns	Major concerns	High	[]
D_Laquinimod:L_Placebo	1	No concerns	Low risk	No concerns	No concerns	Major concerns	Major concerns	High	[]
E_Siponimod:L_Placebo	1	No concerns	Low risk	No concerns	No concerns	Major concerns	Major concerns	High	[]
F_Fingolimod:L_Placebo	1	No concerns	Low risk	No concerns	No concerns	Major concerns	Major concerns	High	[]
K_Dimethyl Fumarate:L_Placebo	1	No concerns	Low risk	No concerns	No concerns	Major concerns	Major concerns	High	[]
A_Ocrelizumab:B_Natalizumab	0	No concerns	Low risk	No concerns	No concerns	Major concerns	Major concerns	High	[]
A_Ocrelizumab:C_Rituximab	0	No concerns	Low risk	No concerns	No concerns	Major concerns	Major concerns	High	[]
A_Ocrelizumab:D_Laquinimod	0	No concerns	Low risk	No concerns	No concerns	Major concerns	Major concerns	High	[]
A_Ocrelizumab:E_Siponimod	0	No concerns	Low risk	No concerns	No concerns	Major concerns	Major concerns	High	[]
A_Ocrelizumab:F_Fingolimod	0	No concerns	Low risk	No concerns	No concerns	Major concerns	Major concerns	High	[]
A_Ocrelizumab:K_Dimethyl Fumarate	0	No concerns	Low risk	No concerns	Some concerns	Some concerns	Major concerns	High	[]
B_Natalizumab:C_Rituximab	0	No concerns	Low risk	No concerns	No concerns	Major concerns	Major concerns	High	[]
B_Natalizumab:D_Laquinimod	0	No concerns	Low risk	No concerns	No concerns	Major concerns	Major concerns	High	[]
B_Natalizumab:E_Siponimod	0	No concerns	Low risk	No concerns	No concerns	Major concerns	Major concerns	High	[]
B_Natalizumab:F_Fingolimod	0	No concerns	Low risk	No concerns	No concerns	Major concerns	Major concerns	High	[]
B_Natalizumab:K_Dimethyl Fumarate	0	No concerns	Low risk	No concerns	No concerns	Major concerns	Major concerns	High	[]
C_Rituximab:D_Laquinimod	0	No concerns	Low risk	No concerns	No concerns	Major concerns	Major concerns	High	[]
C_Rituximab:E_Siponimod	0	No concerns	Low risk	No concerns	No concerns	Major concerns	Major concerns	High	[]
C_Rituximab:F_Fingolimod	0	No concerns	Low risk	No concerns	No concerns	Major concerns	Major concerns	High	[]
C_Rituximab:K_Dimethyl Fumarate	0	No concerns	Low risk	No concerns	No concerns	Major concerns	Major concerns	High	[]
D_Laquinimod:E_Siponimod	0	No concerns	Low risk	No concerns	Some concerns	Some concerns	Major concerns	High	[]
D_Laquinimod:F_Fingolimod	0	No concerns	Low risk	No concerns	No concerns	Major concerns	Major concerns	High	[]
D_Laquinimod:K_Dimethyl Fumarate	0	No concerns	Low risk	No concerns	No concerns	Major concerns	Major concerns	High	[]
E_Siponimod:F_Fingolimod	0	No concerns	Low risk	No concerns	No concerns	Major concerns	Major concerns	High	[]
E_Siponimod:K_Dimethyl Fumarate	0	No concerns	Low risk	No concerns	Some concerns	Some concerns	Major concerns	High	[]
F_Fingolimod:K_Dimethyl Fumarate	0	No concerns	Low risk	No concerns	No concerns	Major concerns	Major concerns	High	[]
**8. SAEs**									
A_Ocrelizumab:L_Placebo	1	No concerns	Low risk	No concerns	Major concerns	No concerns	Major concerns	High	[]
B_Natalizumab:L_Placebo	1	No concerns	Low risk	No concerns	Some concerns	Some concerns	Major concerns	High	[]
C_Rituximab:L_Placebo	1	No concerns	Low risk	No concerns	Major concerns	No concerns	Major concerns	High	[]

D_Laquinimod:L_Placebo	1	No concerns	Low risk	No concerns	Major concerns	No concerns	Major concerns	High	[]
E_Siponimod:L_Placebo	1	No concerns	Low risk	No concerns	Some concerns	Some concerns	Major concerns	High	[]
F_Fingolimod:L_Placebo	1	No concerns	Low risk	No concerns	Some concerns	Some concerns	Major concerns	High	[]
K_Dimethyl Fumarate:L_Placebo	1	No concerns	Low risk	No concerns	Major concerns	No concerns	Major concerns	High	[]
A_Ocrelizumab:B_Natalizumab	0	No concerns	Low risk	No concerns	Major concerns	No concerns	Major concerns	High	[]
A_Ocrelizumab:C_Rituximab	0	No concerns	Low risk	No concerns	Major concerns	No concerns	Major concerns	High	[]
A_Ocrelizumab:D_Laquinimod	0	No concerns	Low risk	No concerns	Major concerns	No concerns	Major concerns	High	[]
A_Ocrelizumab:E_Siponimod	0	No concerns	Low risk	No concerns	Some concerns	Some concerns	Major concerns	High	[]
A_Ocrelizumab:F_Fingolimod	0	No concerns	Low risk	No concerns	Major concerns	No concerns	Major concerns	High	[]
A_Ocrelizumab:K_Dimethyl Fumarate	0	No concerns	Low risk	No concerns	Major concerns	No concerns	Major concerns	High	[]
B_Natalizumab:C_Rituximab	0	No concerns	Low risk	No concerns	Major concerns	No concerns	Major concerns	High	[]
B_Natalizumab:D_Laquinimod	0	No concerns	Low risk	No concerns	Major concerns	No concerns	Major concerns	High	[]
B_Natalizumab:E_Siponimod	0	No concerns	Low risk	No concerns	Some concerns	Some concerns	Major concerns	High	[]
B_Natalizumab:F_Fingolimod	0	No concerns	Low risk	No concerns	Major concerns	No concerns	Major concerns	High	[]
B_Natalizumab:K_Dimethyl Fumarate	0	No concerns	Low risk	No concerns	Major concerns	No concerns	Major concerns	High	[]
C_Rituximab:D_Laquinimod	0	No concerns	Low risk	No concerns	Major concerns	No concerns	Major concerns	High	[]
C_Rituximab:E_Siponimod	0	No concerns	Low risk	No concerns	Major concerns	No concerns	Major concerns	High	[]
C_Rituximab:F_Fingolimod	0	No concerns	Low risk	No concerns	Major concerns	No concerns	Major concerns	High	[]
C_Rituximab:K_Dimethyl Fumarate	0	No concerns	Low risk	No concerns	Major concerns	No concerns	Major concerns	High	[]
D_Laquinimod:E_Siponimod	0	No concerns	Low risk	No concerns	Major concerns	No concerns	Major concerns	High	[]
D_Laquinimod:F_Fingolimod	0	No concerns	Low risk	No concerns	Major concerns	No concerns	Major concerns	High	[]
D_Laquinimod:K_Dimethyl Fumarate	0	No concerns	Low risk	No concerns	Major concerns	No concerns	Major concerns	High	[]
E_Siponimod:F_Fingolimod	0	No concerns	Low risk	No concerns	Some concerns	Some concerns	Major concerns	High	[]
E_Siponimod:K_Dimethyl Fumarate	0	No concerns	Low risk	No concerns	Major concerns	No concerns	Major concerns	High	[]
F_Fingolimod:K_Dimethyl Fumarate	0	No concerns	Low risk	No concerns	Major concerns	No concerns	Major concerns	High	[]

Figure S1: Risk of bias.

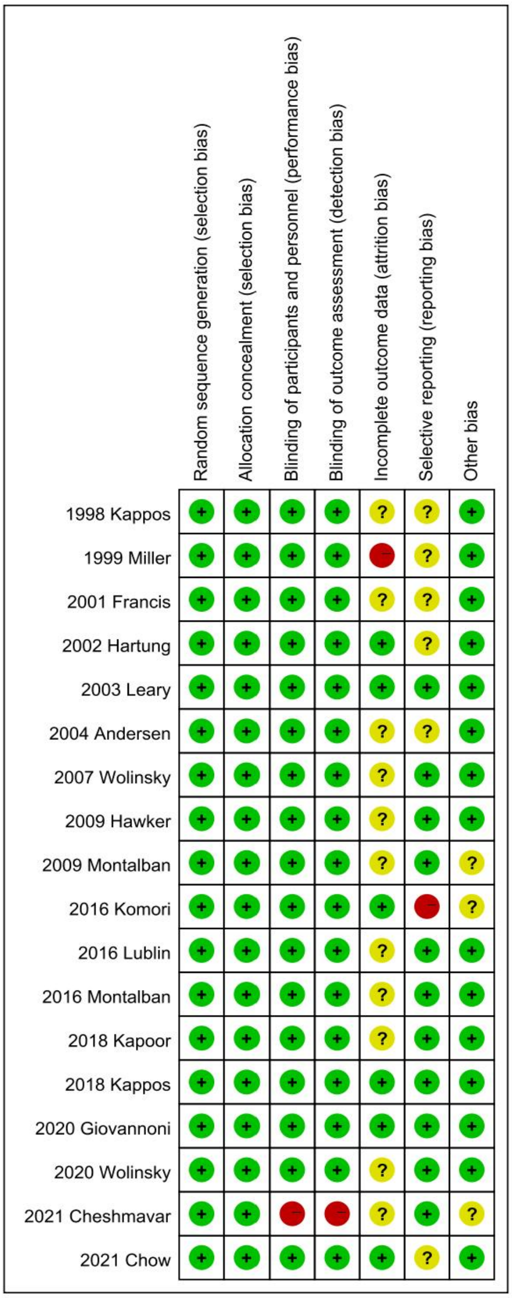

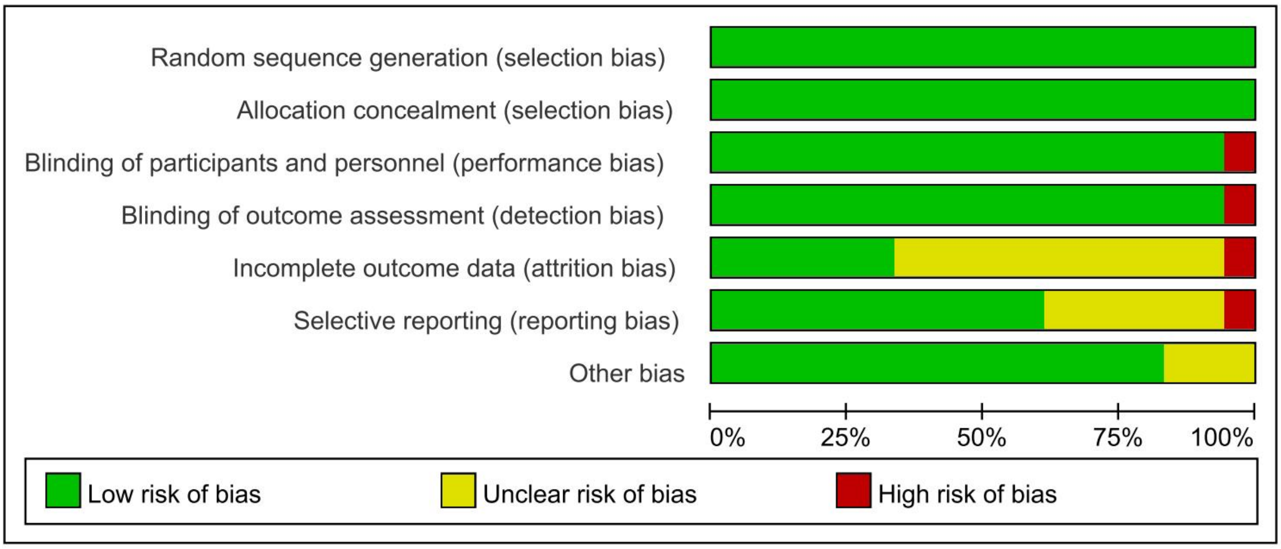

Figure S2: Convergence diagnostics of the network meta-analysis: EDSS.

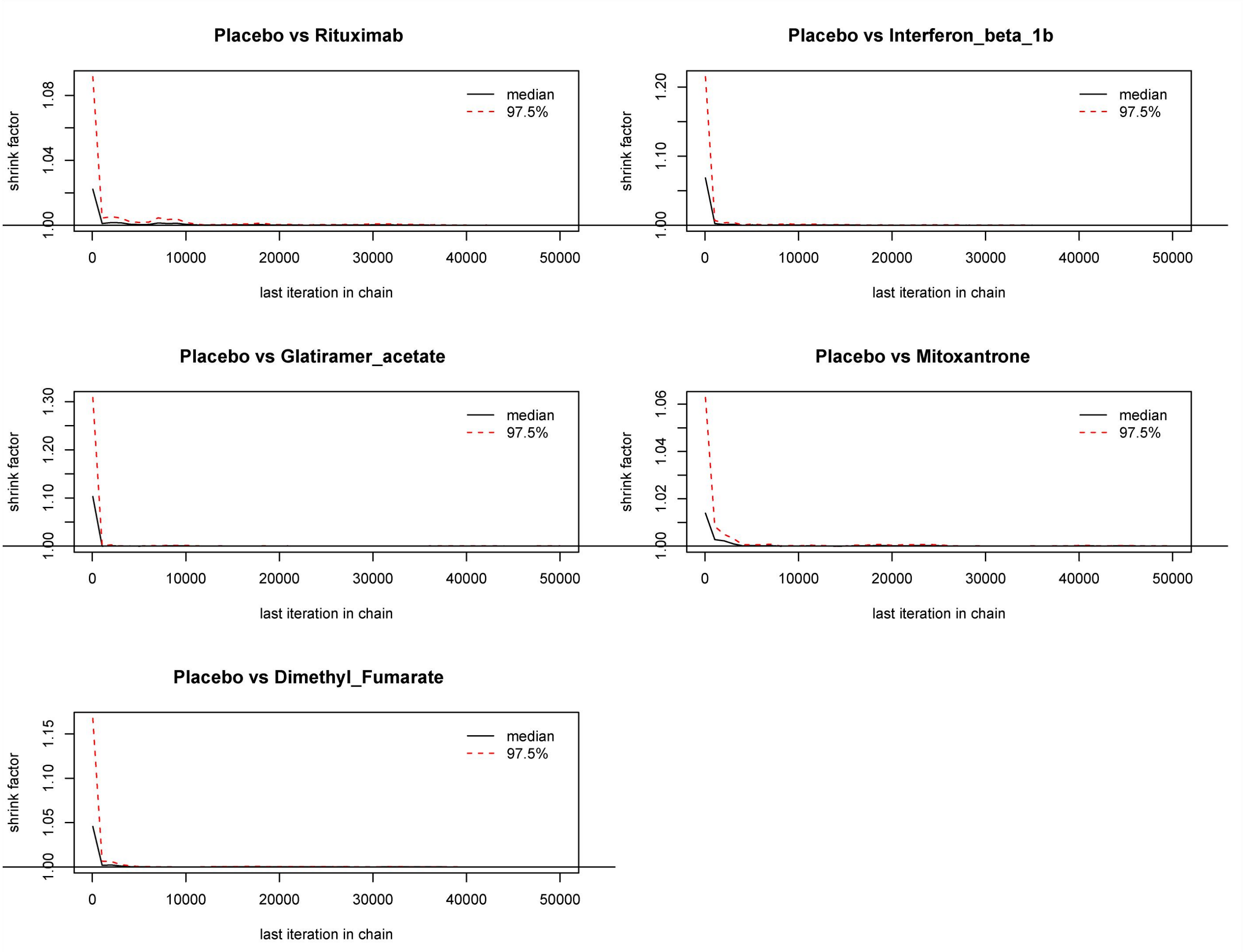

Figure S3: Convergence diagnostics of the network meta-analysis: CDP.

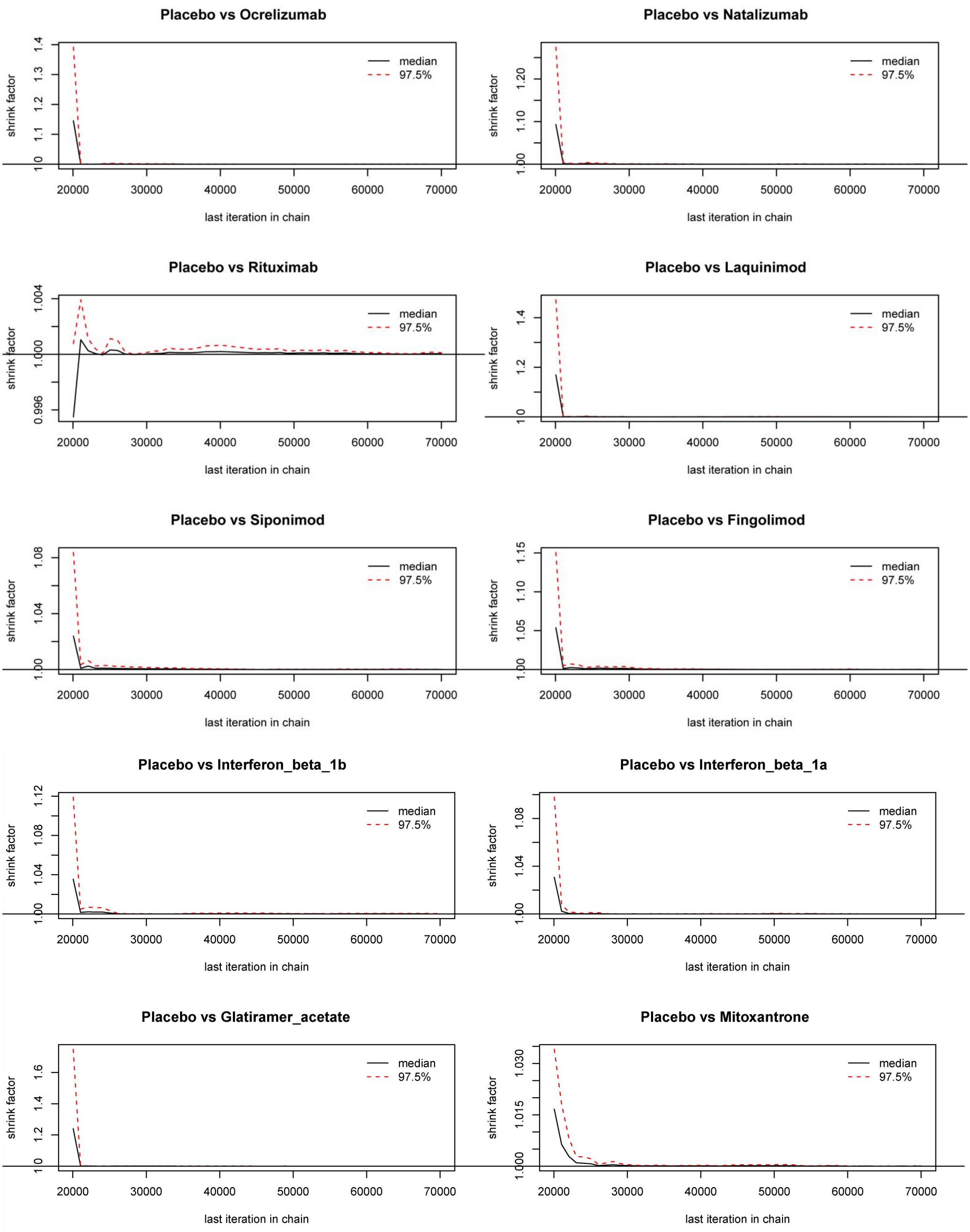

Figure S4: Convergence diagnostics of the network meta-analysis: T25W.

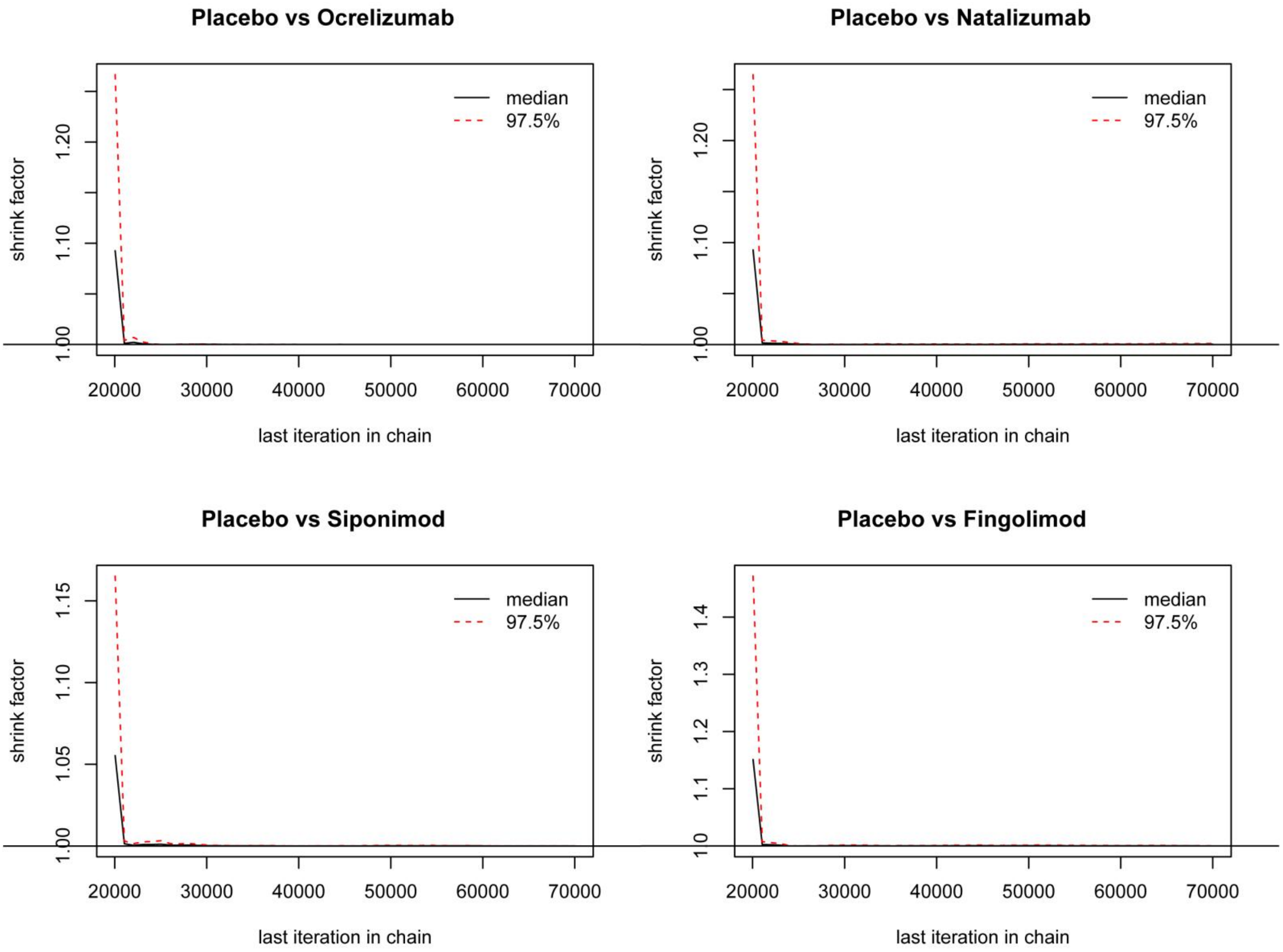

Figure S5: Convergence diagnostics of the network meta-analysis: 9HPT.

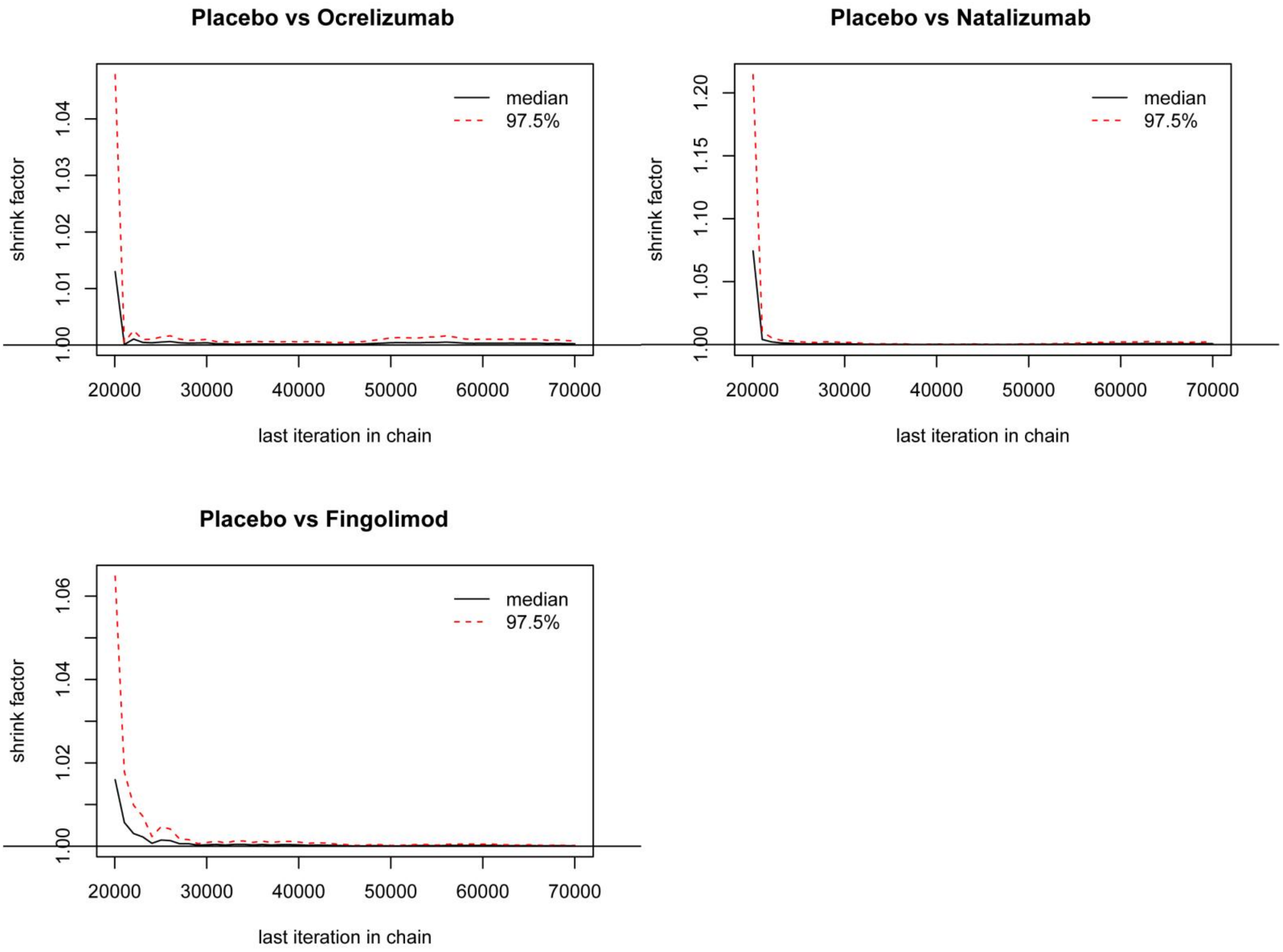

Figure S6: Convergence diagnostics of the network meta-analysis: New or enlarging T2 lesions.

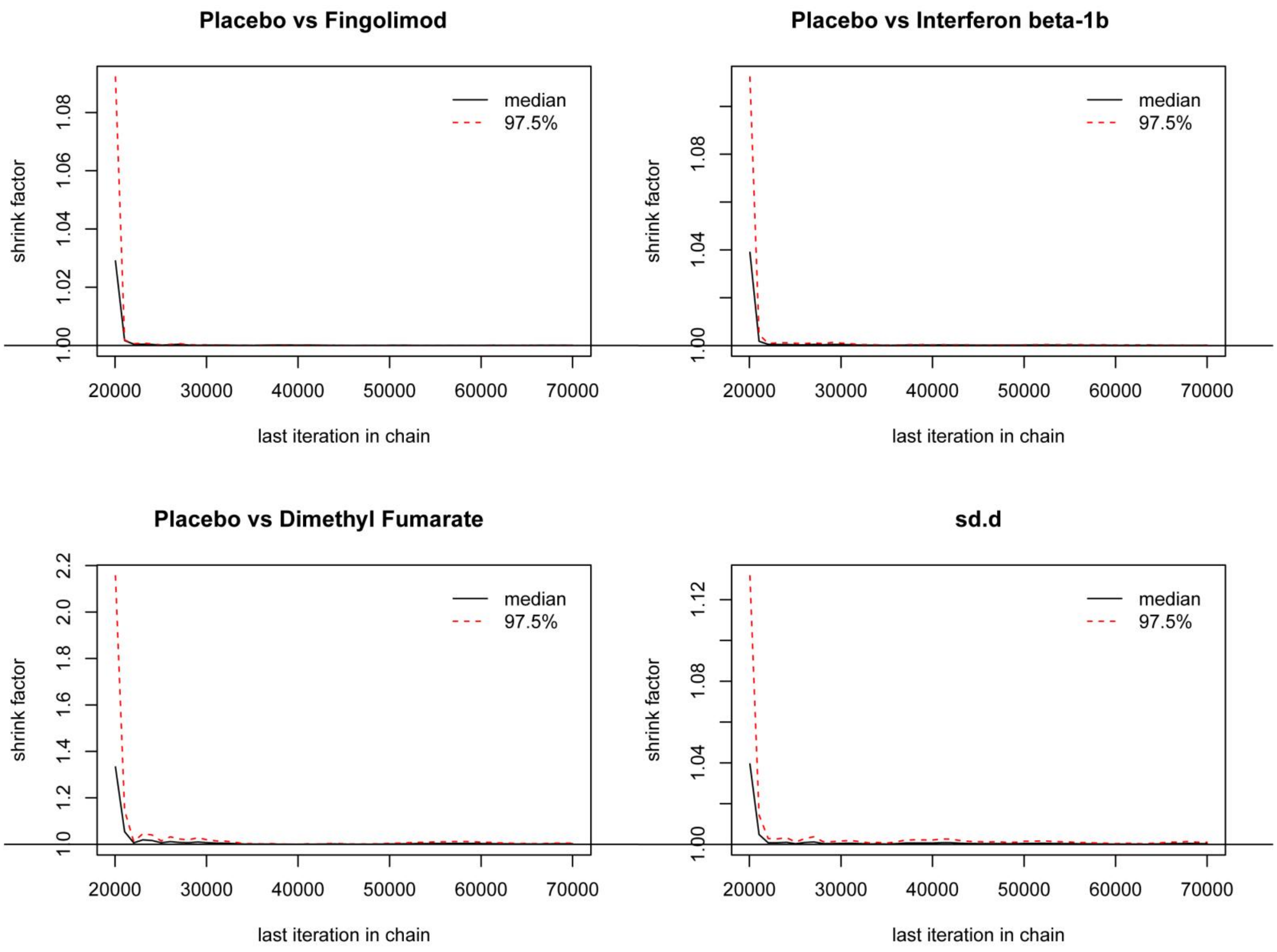

Figure S7: Convergence diagnostics of the network meta-analysis: Change from baseline in total volume of lesions on T2-weighted images (mm³).

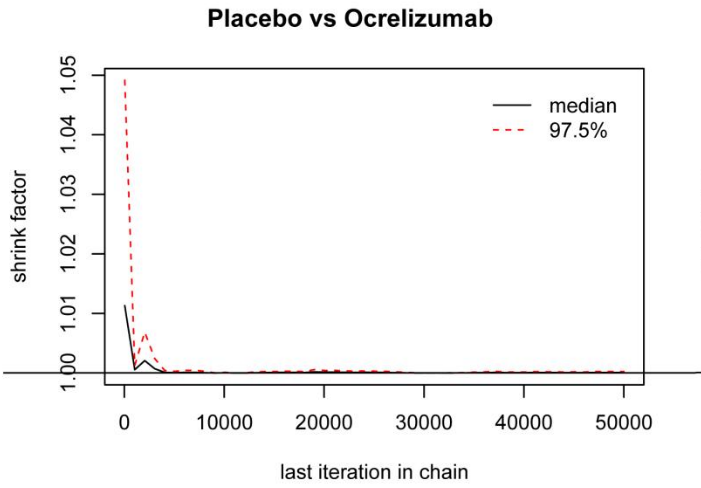

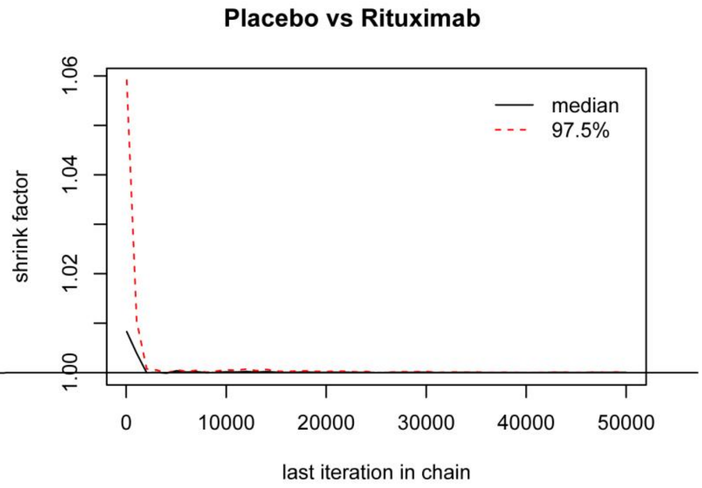

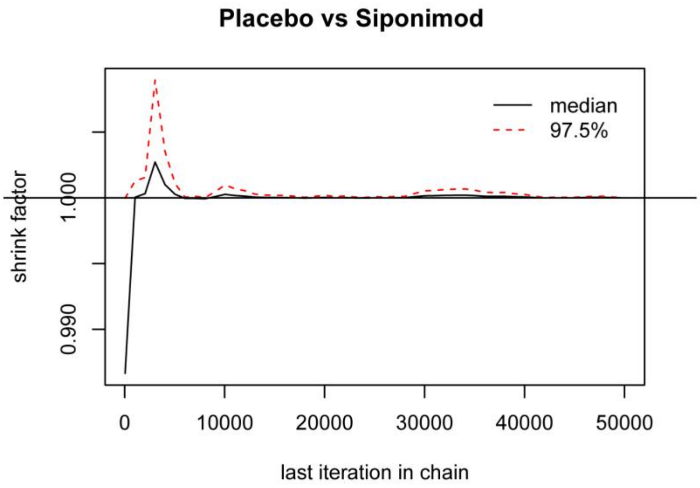

Figure S8: Convergence diagnostics of the network meta-analysis: AEs.

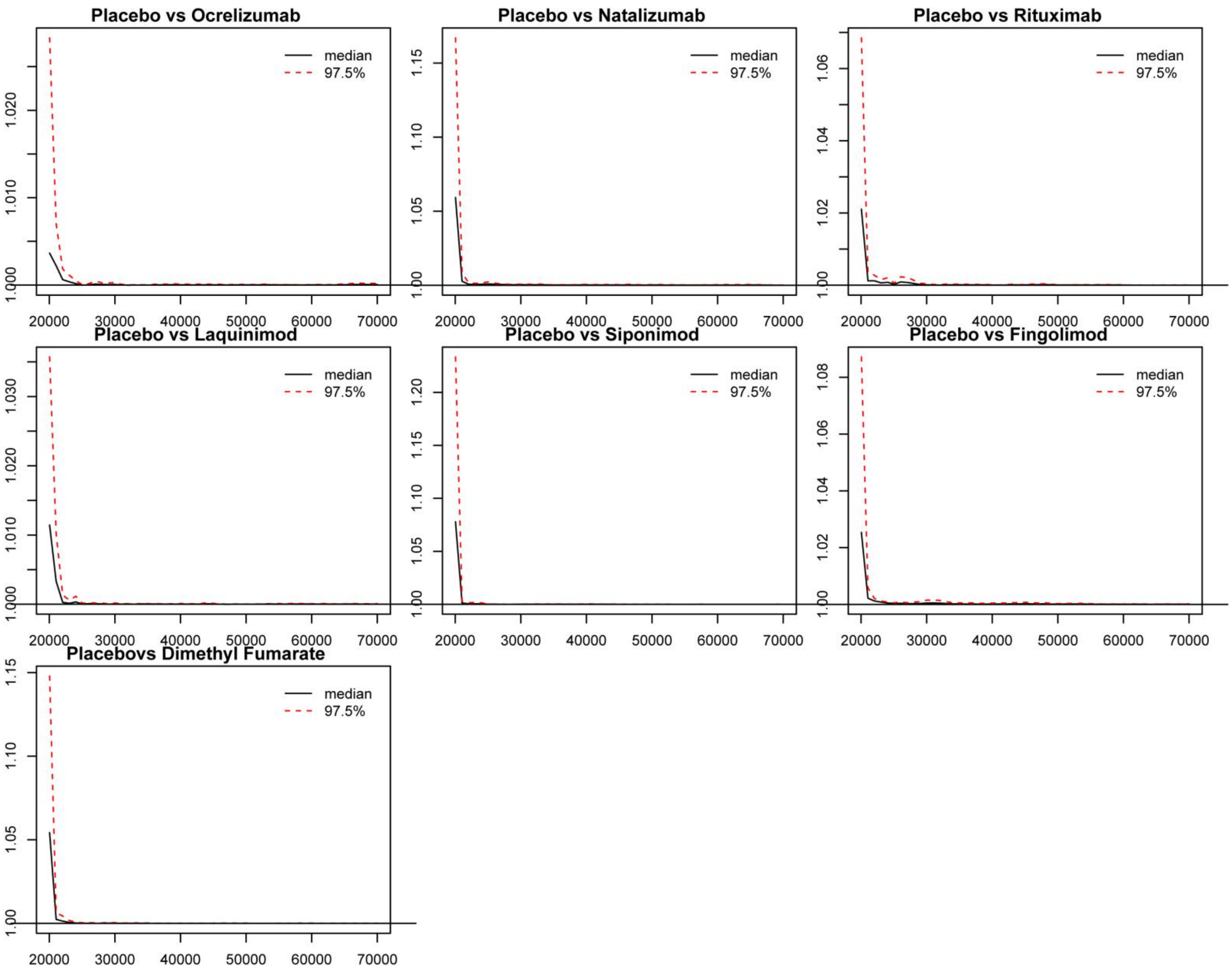

Figure S9: Convergence diagnostics of the network meta-analysis: SAEs.

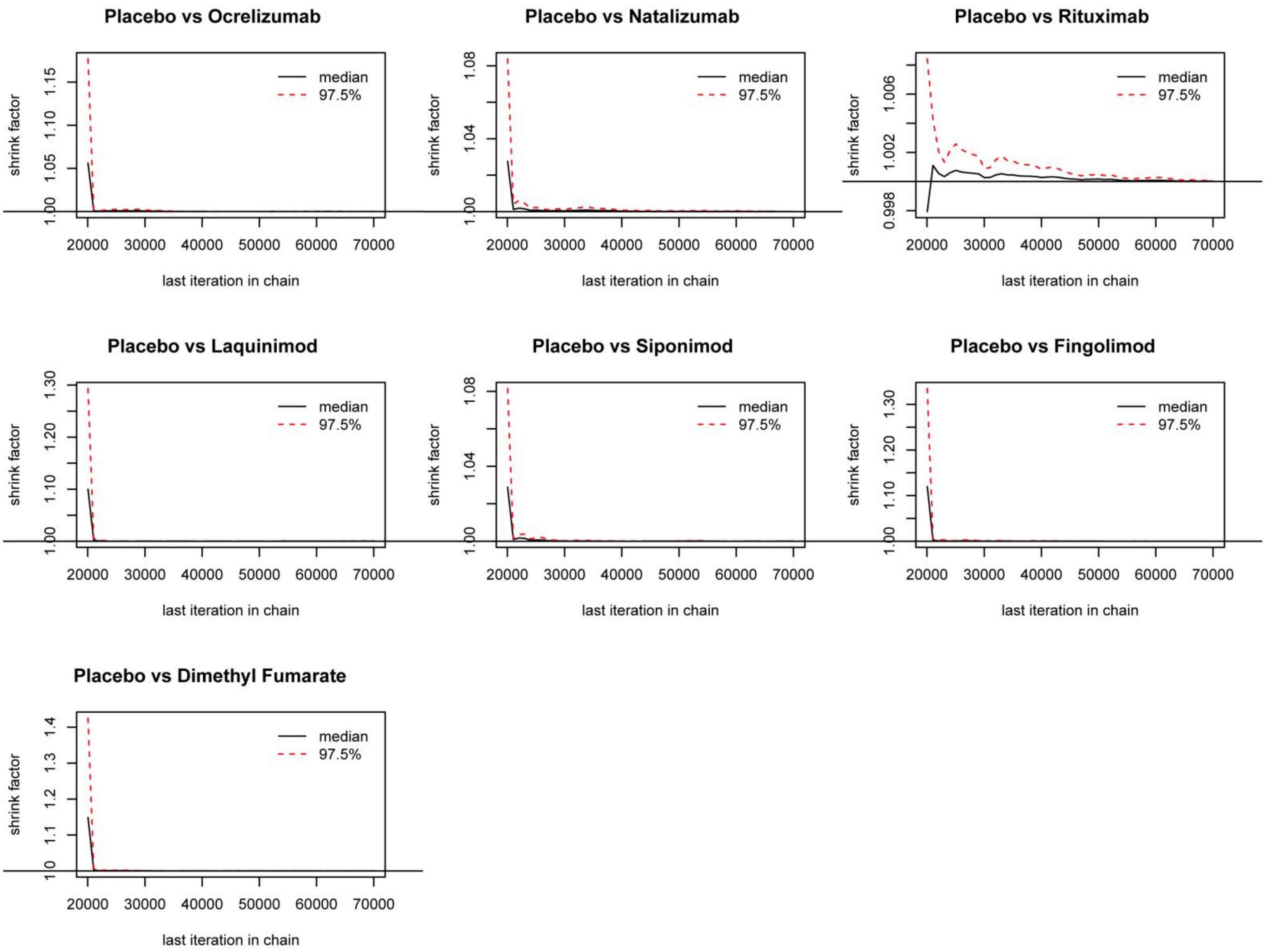

Figure S10: Trace and density of the network meta-analysis: EDSS.

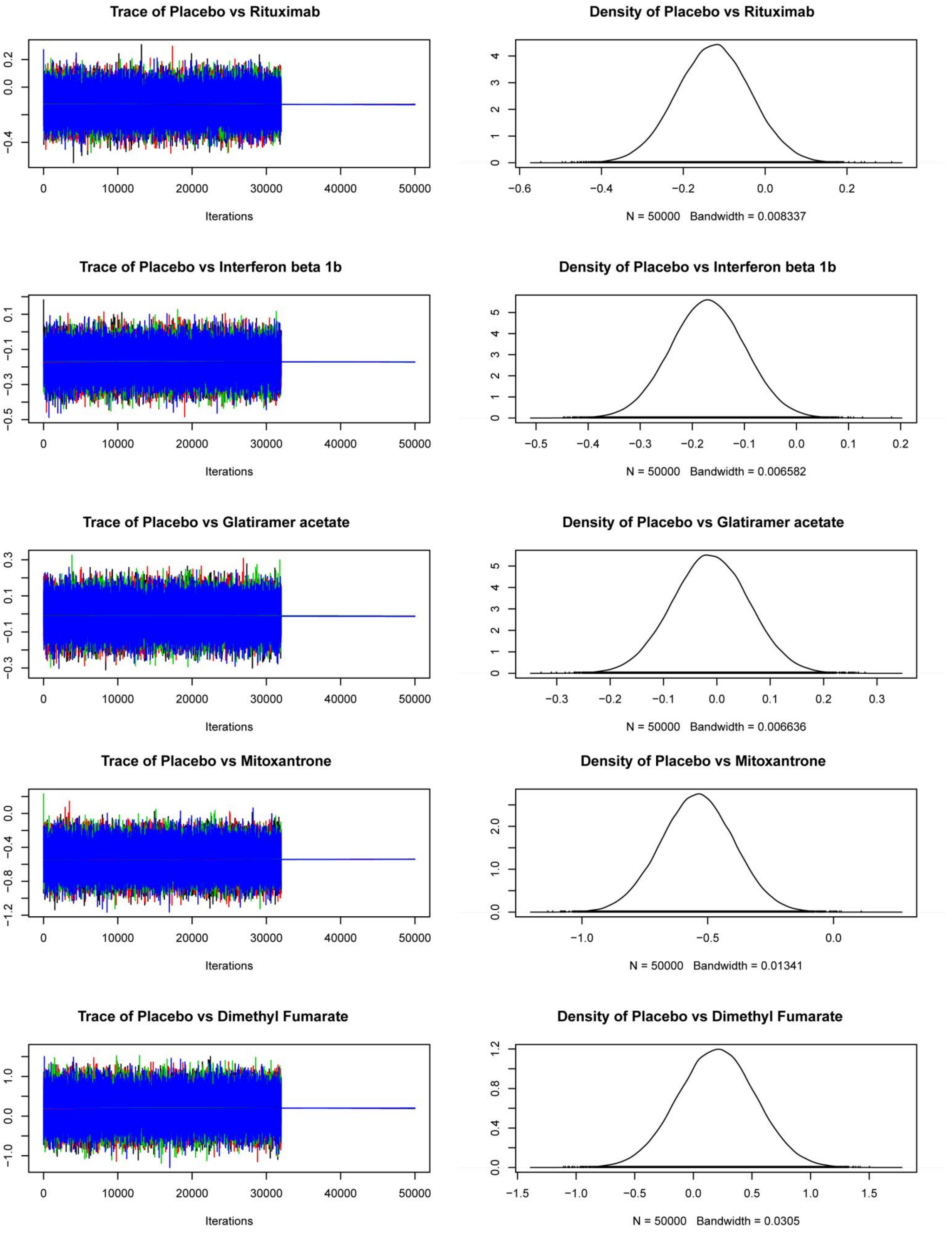

Figure S11: Trace and density of the network meta-analysis: CDP.

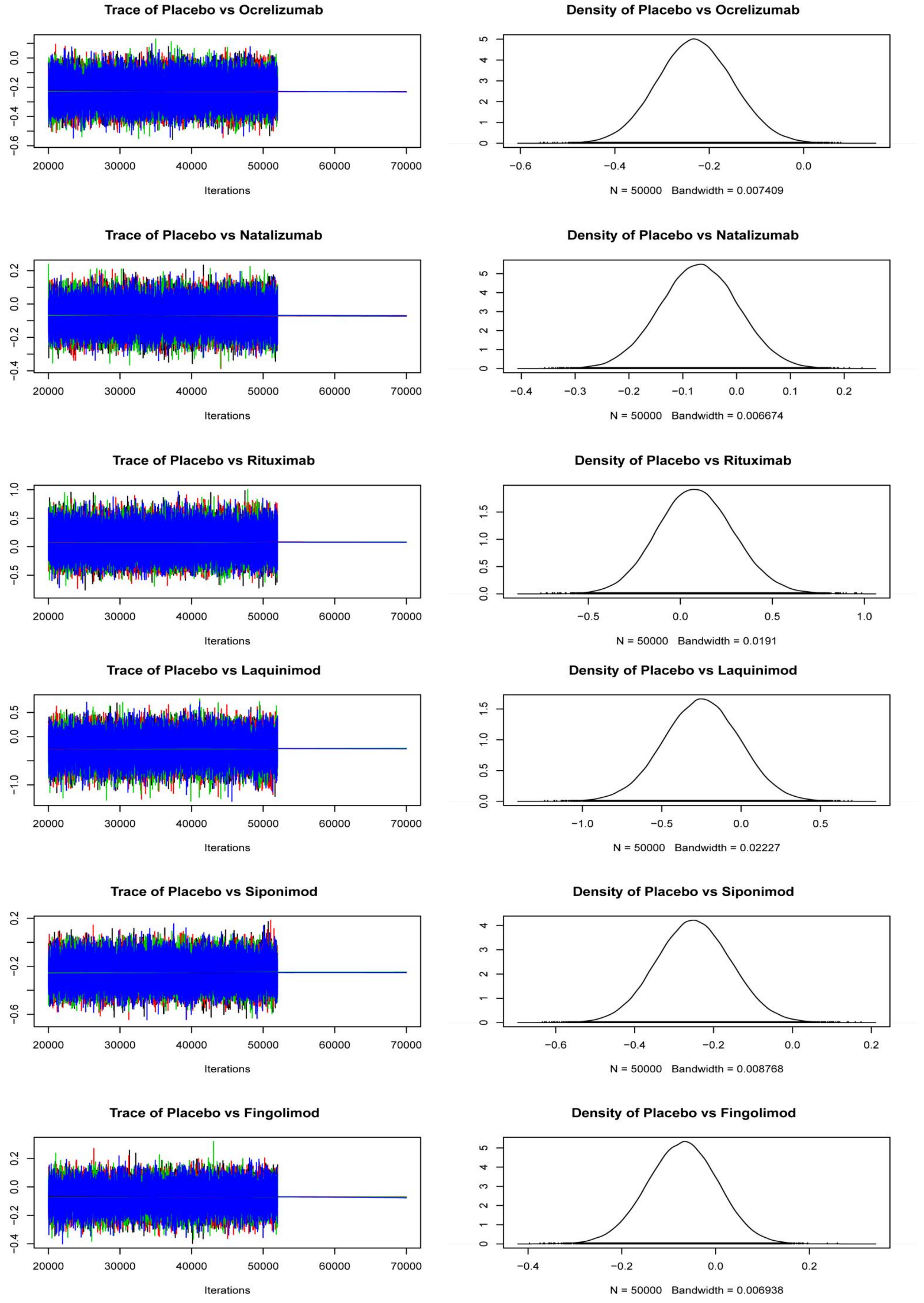

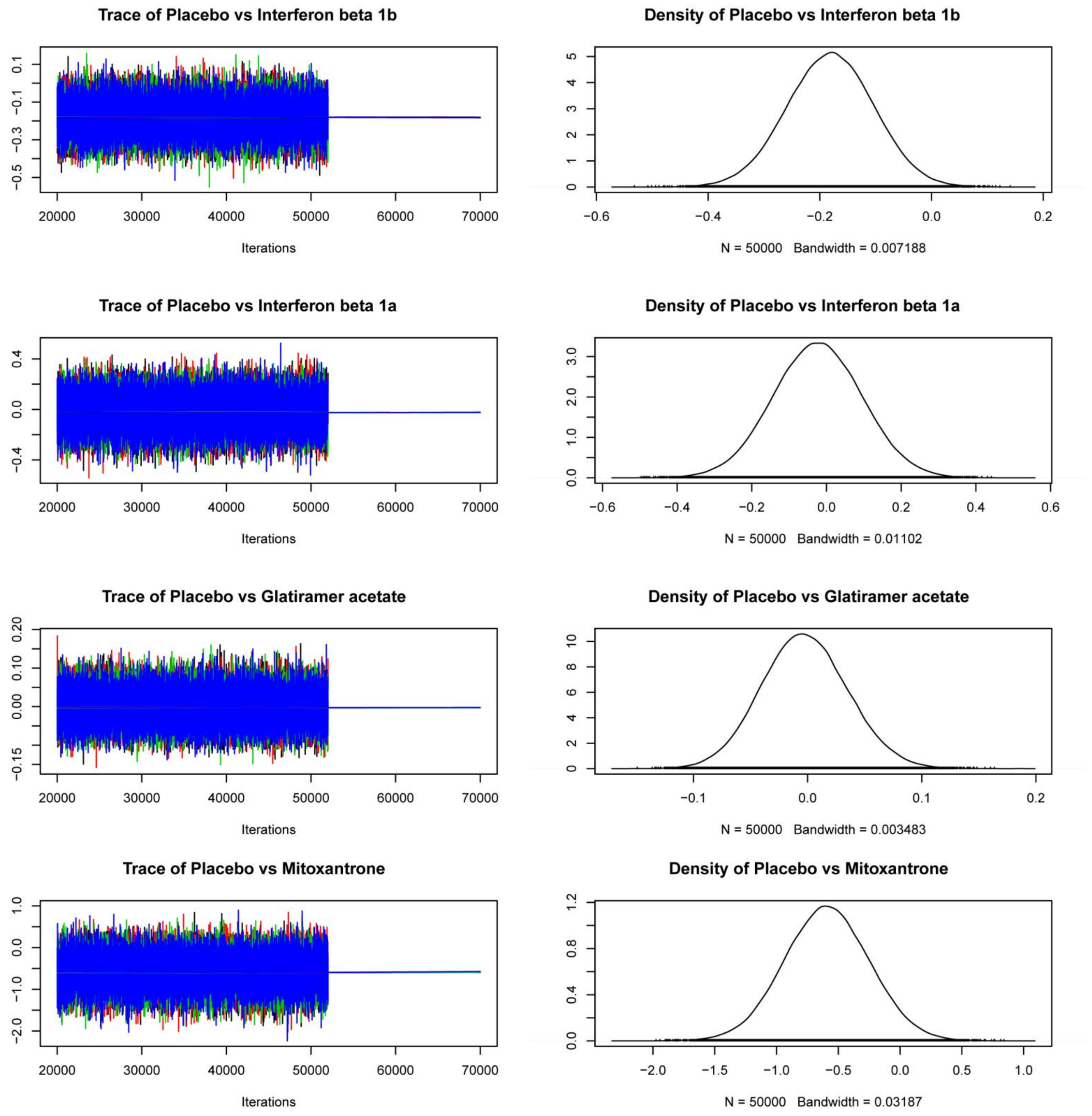
Trace of Placebo vs Interferon beta 1b
Iterations
Density of Placebo vs Interferon beta 1b
N = 50000 Bandwidth = 0.007188
Trace of Placebo vs Interferon beta 1a
Iterations
Density of Placebo vs Interferon beta 1a
N = 50000 Bandwidth = 0.01102
Trace of Placebo vs Glatiramer acetate
Iterations
Density of Placebo vs Glatiramer acetate
N = 50000 Bandwidth = 0.003483
Trace of Placebo vs Mitoxantrone
Iterations
Density of Placebo vs Mitoxantrone
N = 50000 Bandwidth = 0.03187

Figure S12: Trace and density of the network meta-analysis: T25FW.

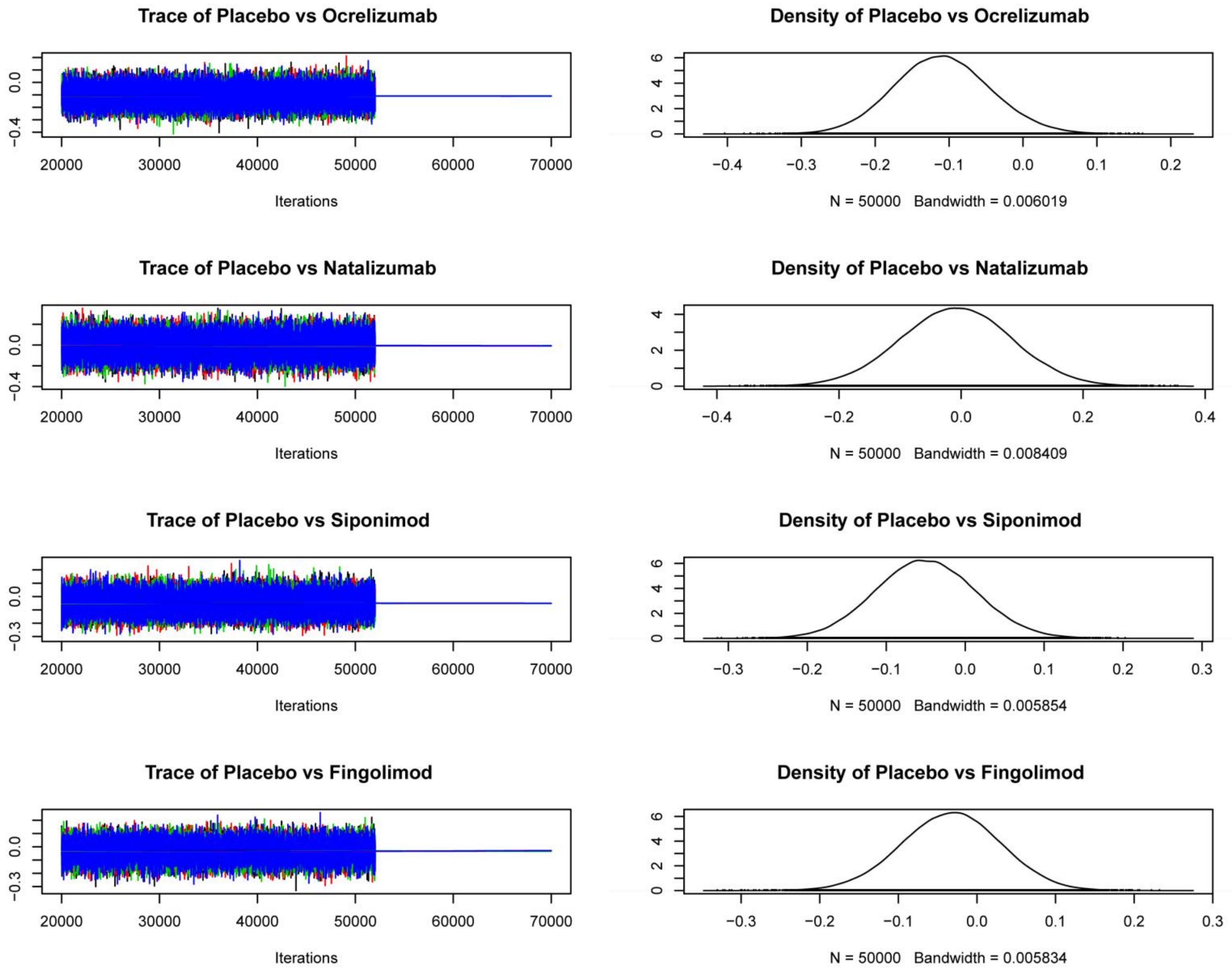

Figure S13: Trace and density of the network meta-analysis: 9HPT.

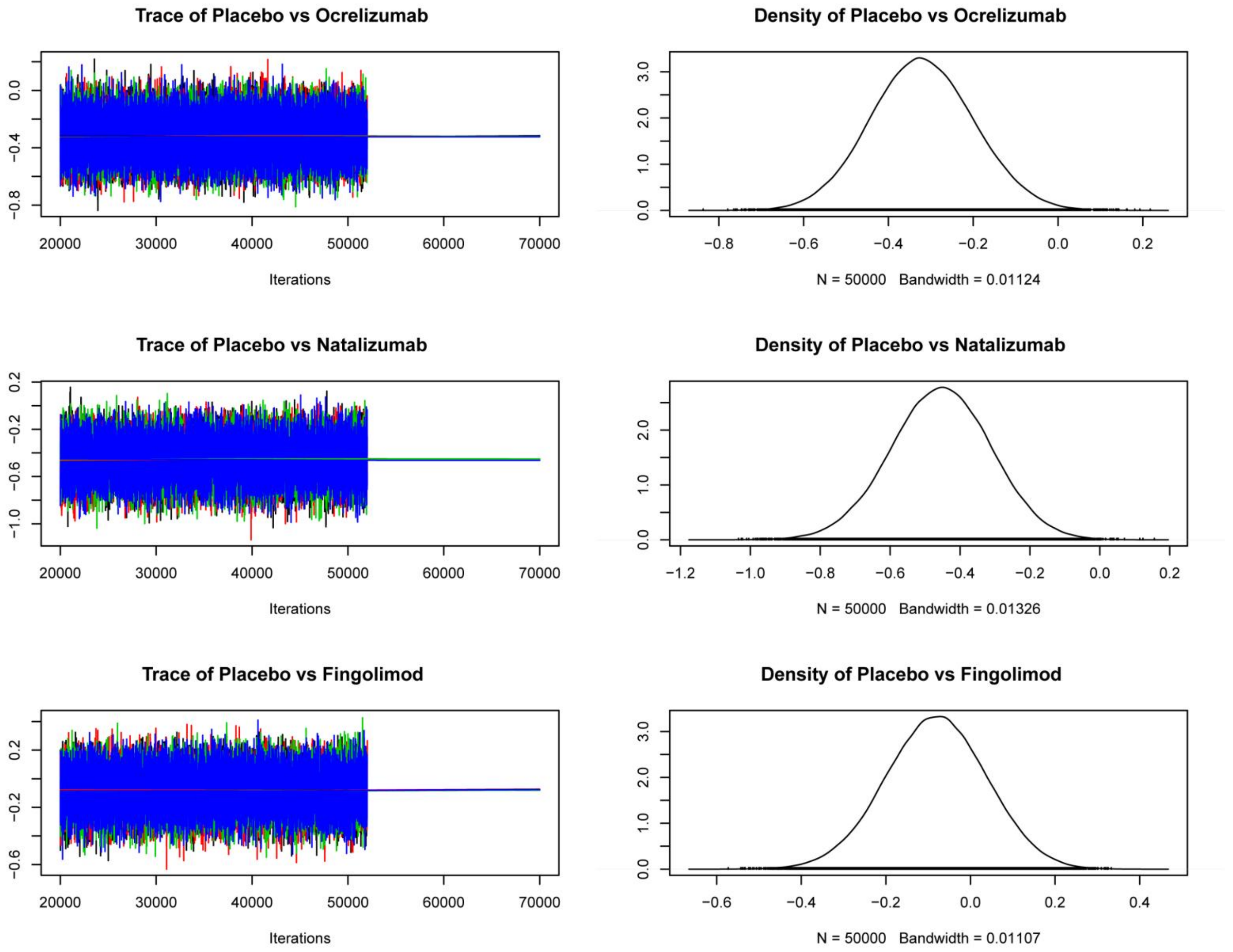

Figure S14: Trace and density of the network meta-analysis: New or enlarging T2 lesions.

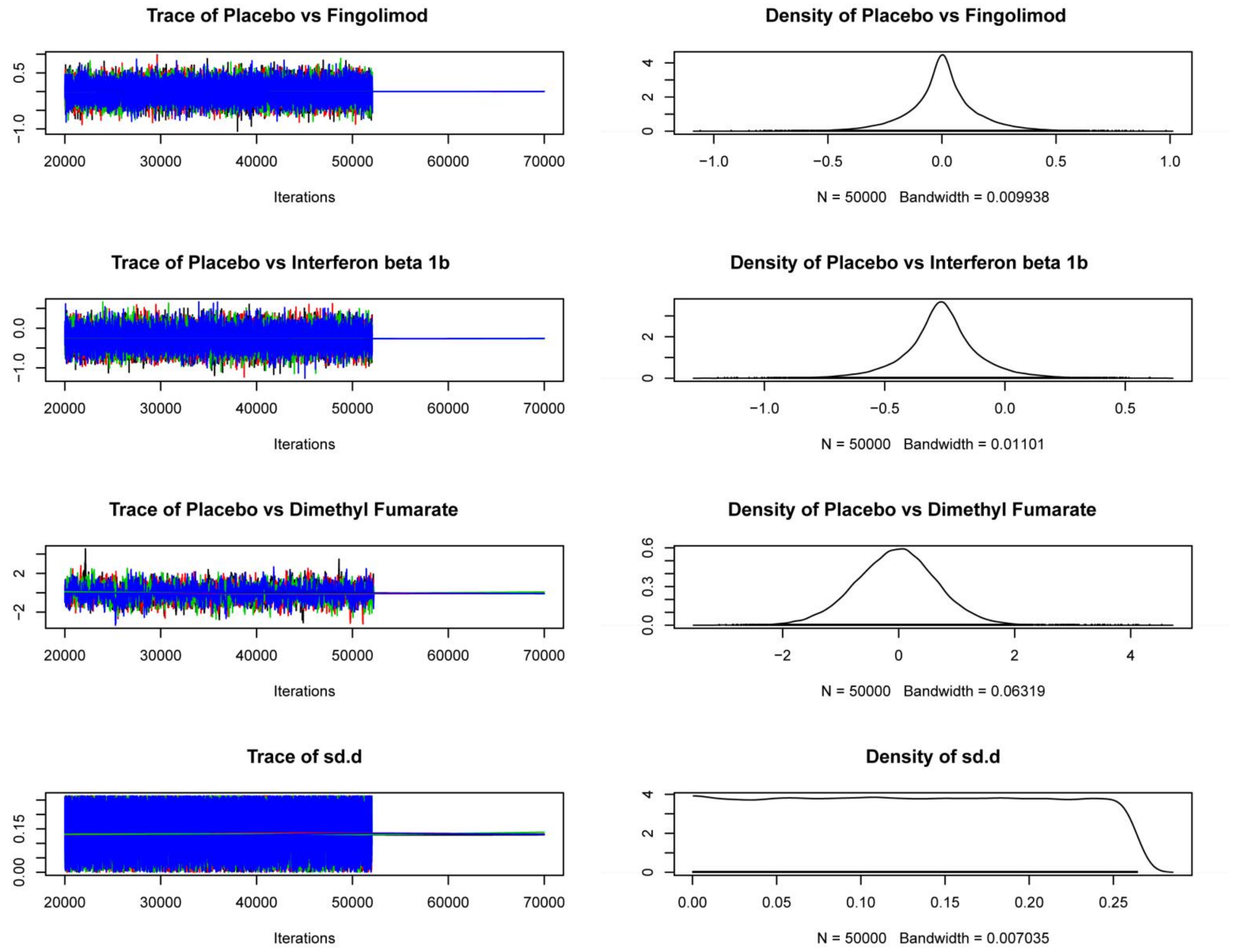

Figure S15: Trace and density of the network meta-analysis: Change from baseline in total volume of lesions on T2-weighted images (mm³).

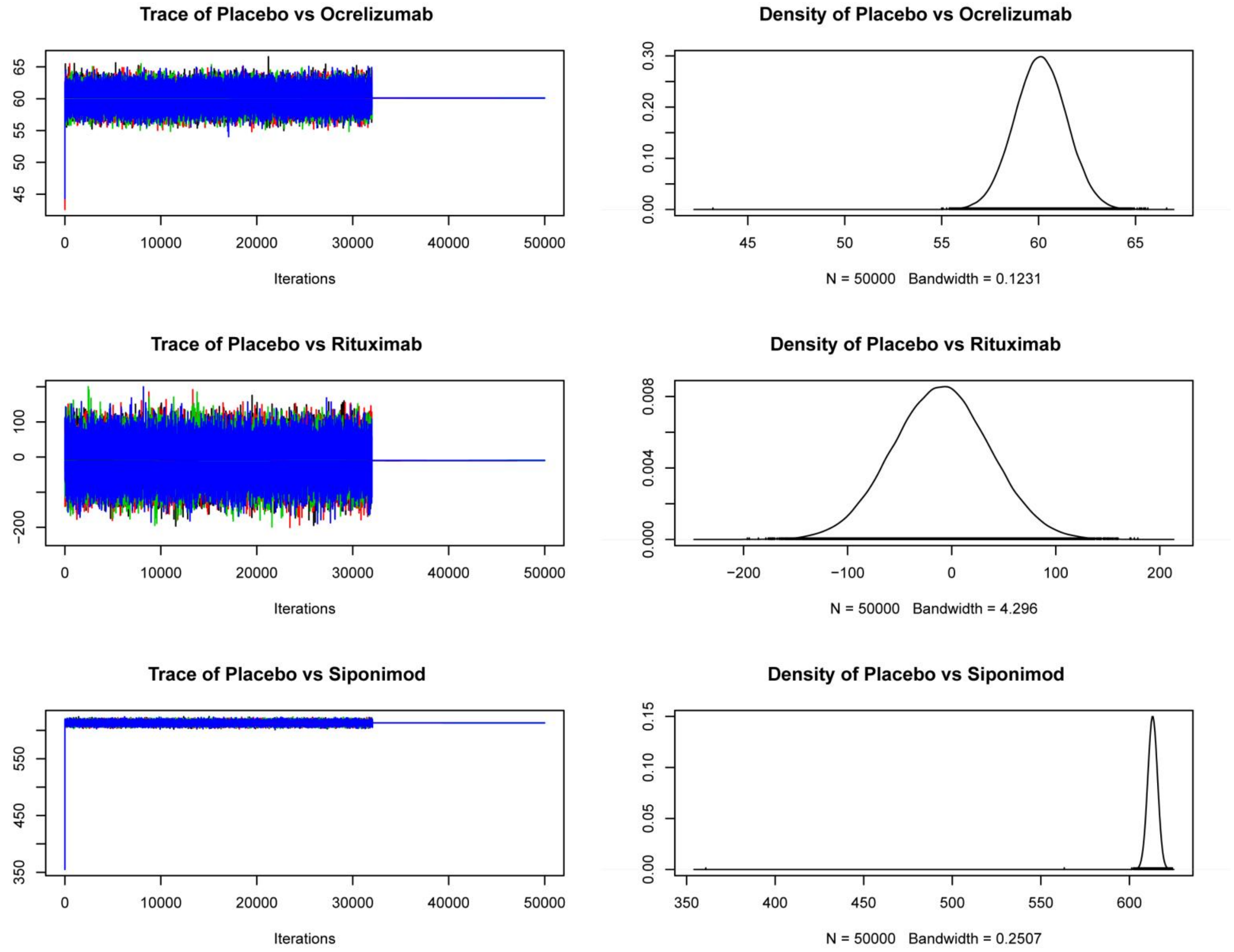

Figure S16: Trace and density of the network meta-analysis: AEs.

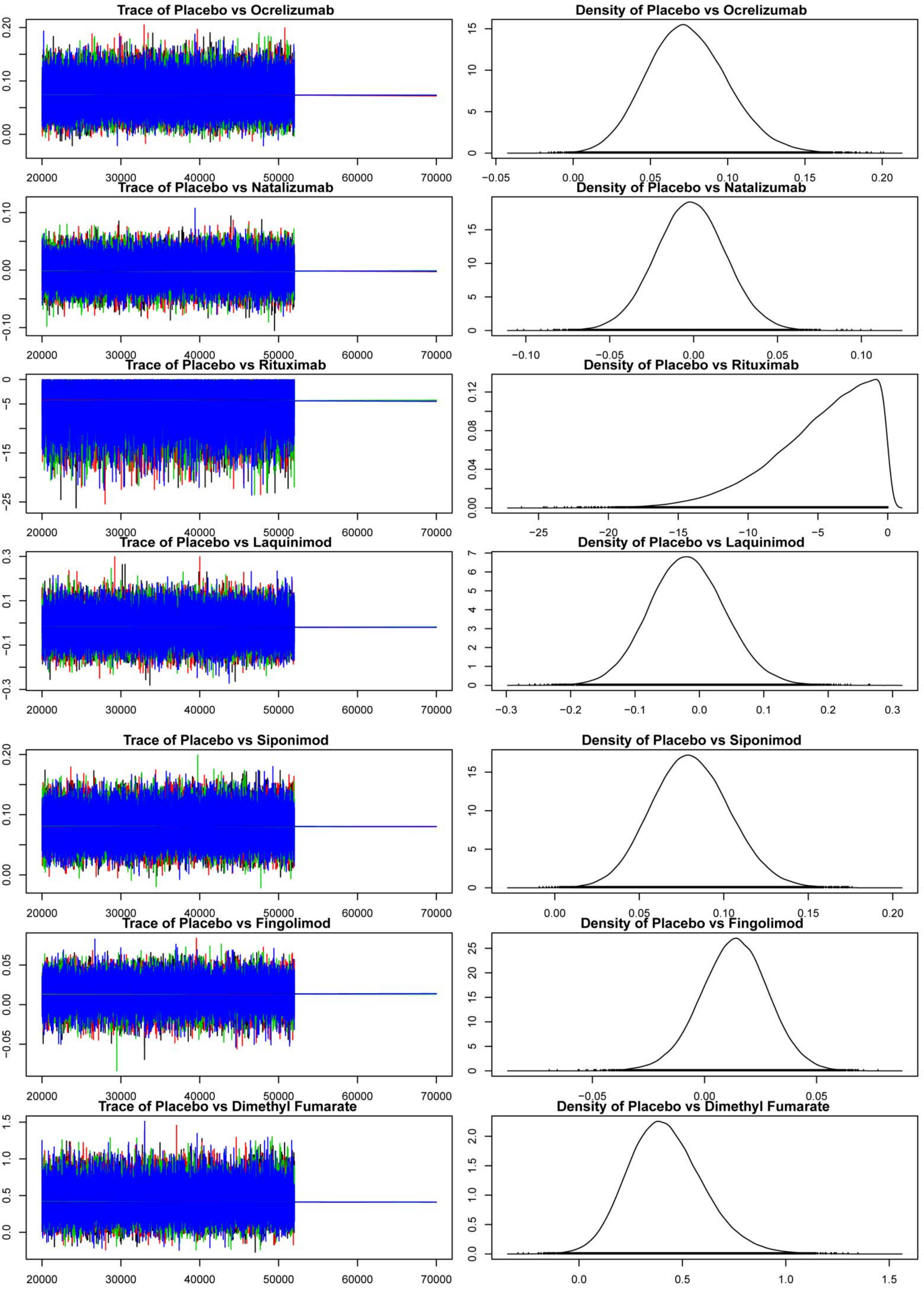

Figure S17: Trace and density of the network meta-analysis: SAEs.

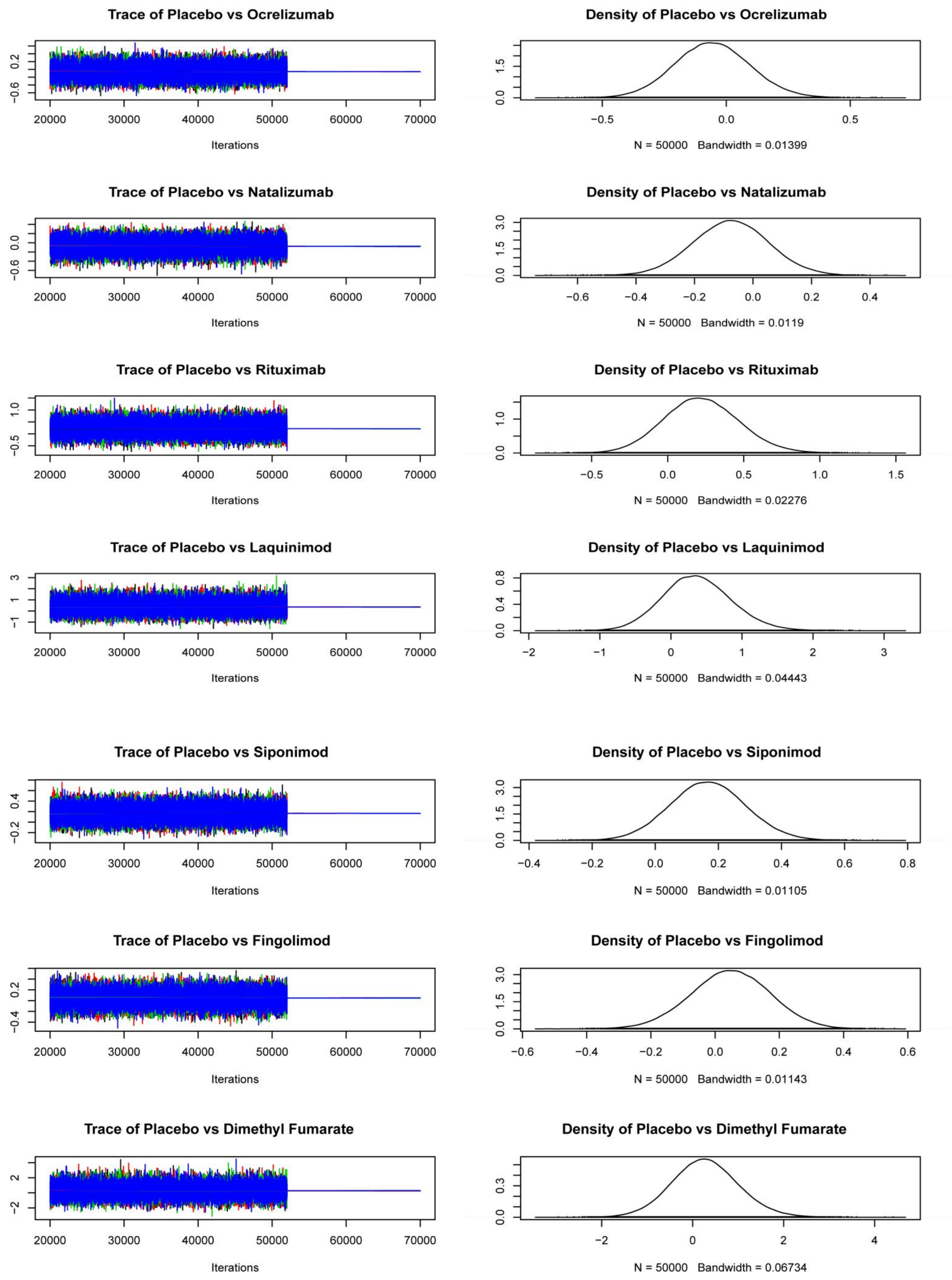

Figure S18: Forest plots for the heterogeneity: EDSS.

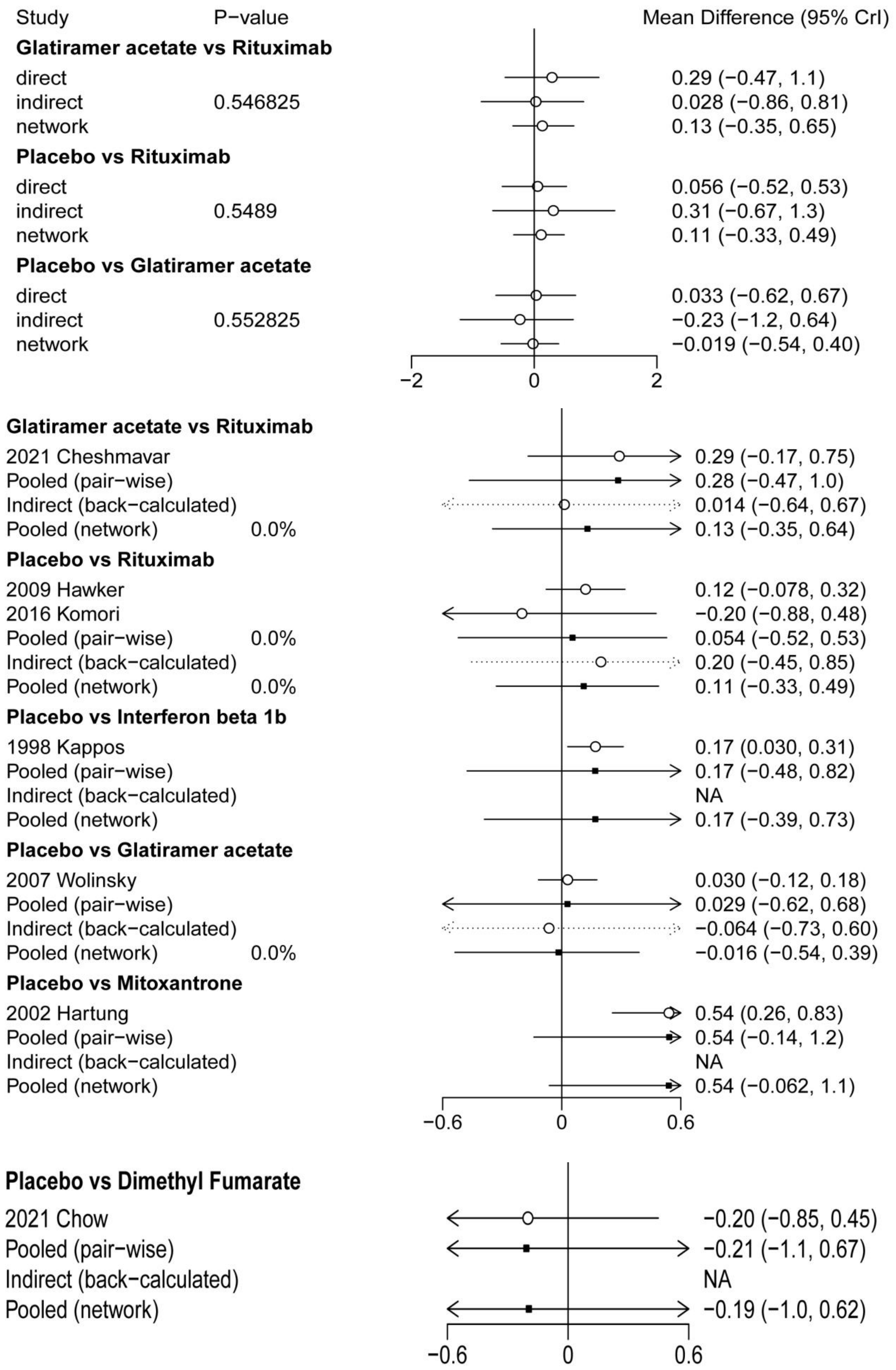

Figure S19: Forest plots for the heterogeneity: CDP.

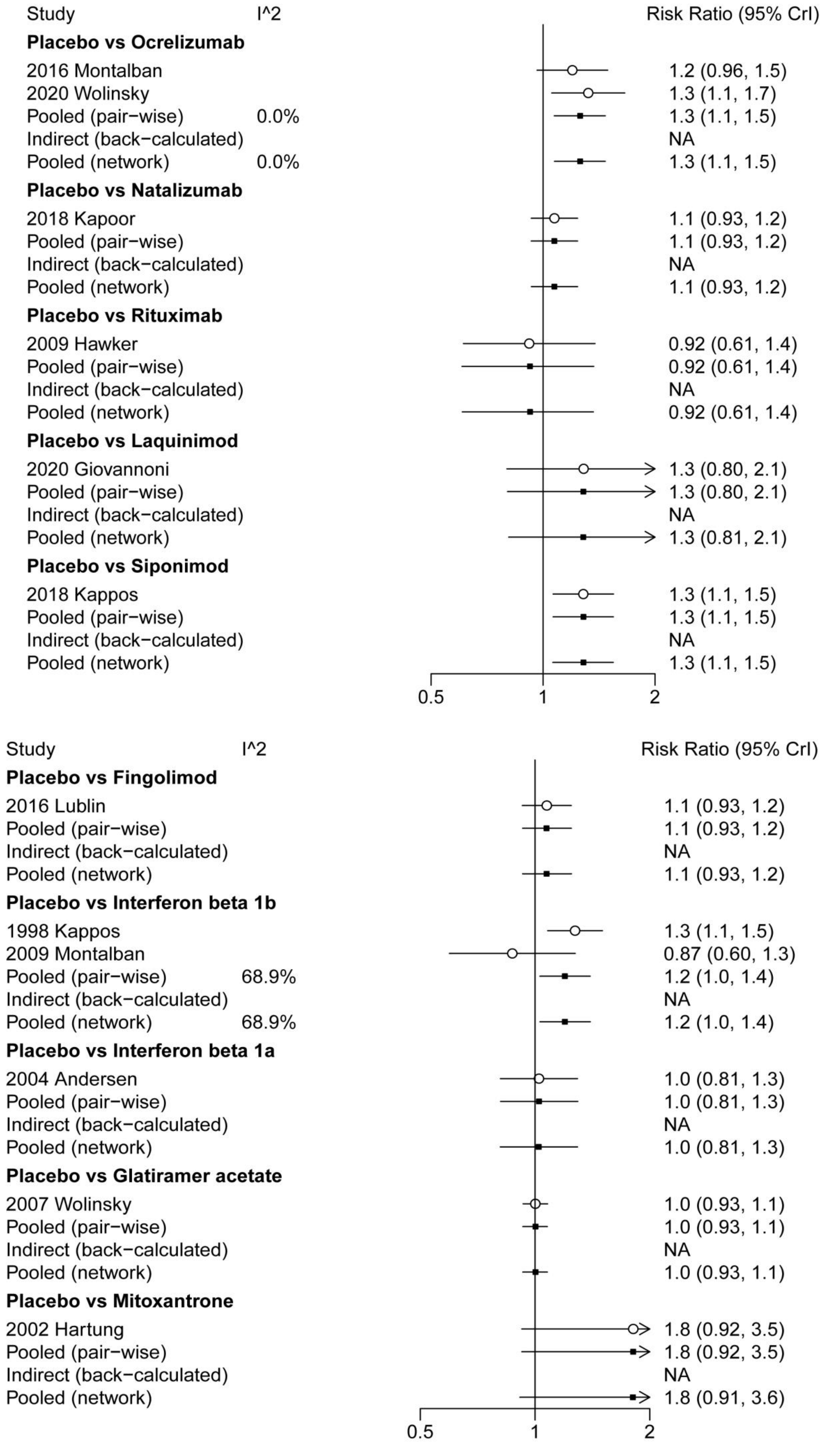

Figure S20: Forest plots for the inconsistency: EDSS.

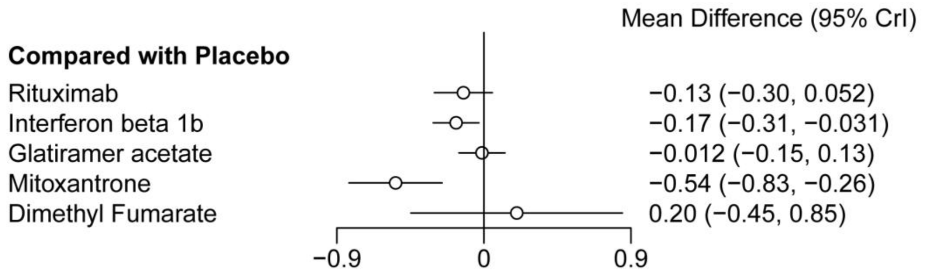

Figure S21: Forest plots for the inconsistency: CDP.

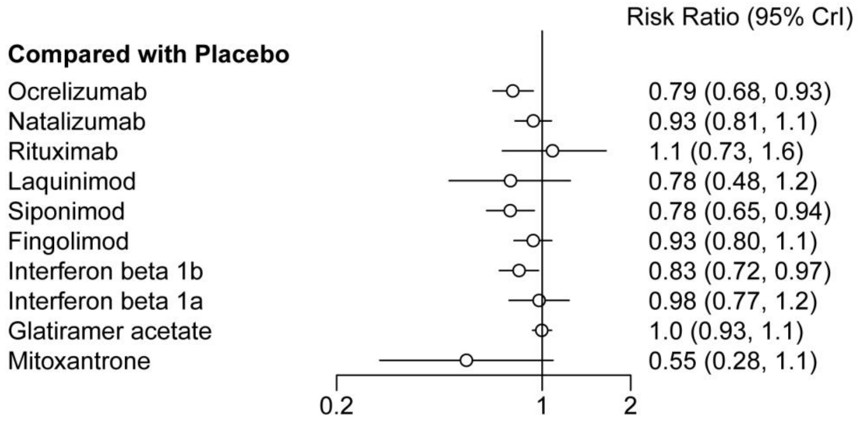

Figure S22: Forest plots for the inconsistency: T25FW.

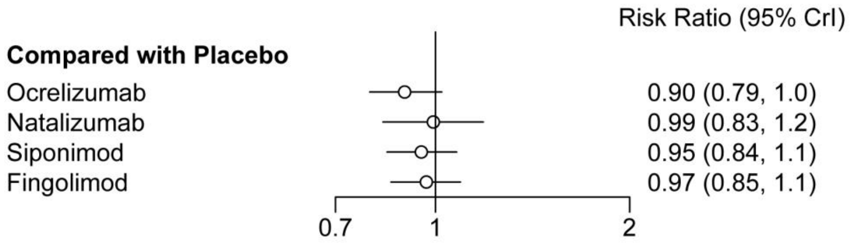

Figure S23: Forest plots for the inconsistency: 9HPT.

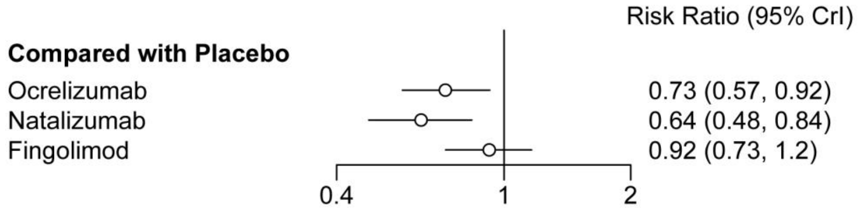

Figure S24: Forest plots for the inconsistency: New or enlarging T2 lesions.

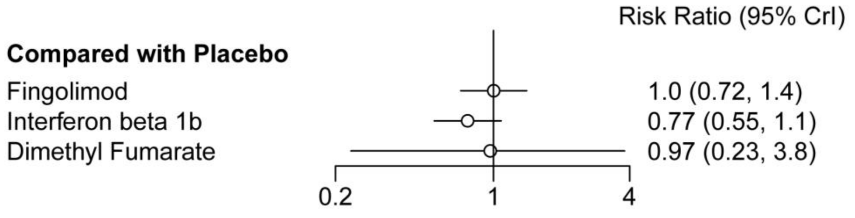

Figure S25: Forest plots for the inconsistency: Change from baseline in total volume of lesions on T2-weighted images (mm^3).

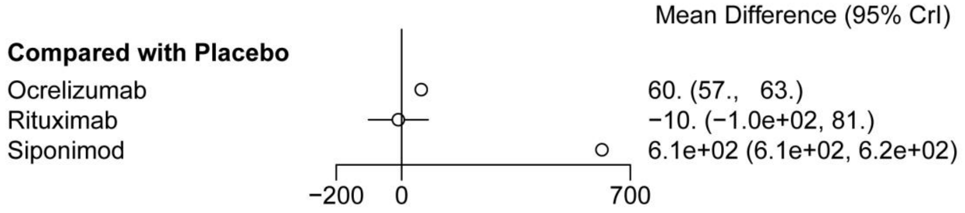

Figure S26: Forest plots for the inconsistency: AEs.

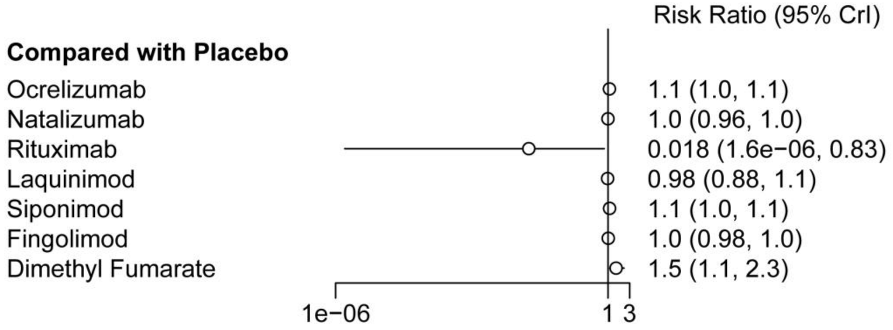

Figure S27: Forest plots for the inconsistency: SAEs.

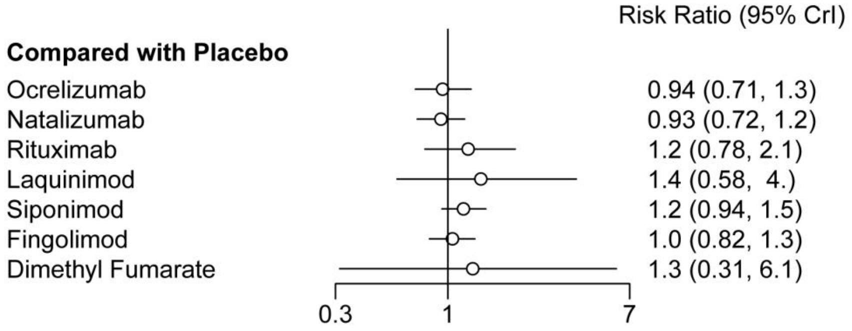

Supplement: Supplementary file 1 [file Data_Sheet_1.PDF]
